# Disparities in blood cancer survival in the UK 2009–2019: national cohort studies

**DOI:** 10.1038/s44276-026-00222-0

**Published:** 2026-04-15

**Authors:** Janice Hoang, Joshua Allen, Rebecca Capel, Rebecca Thomas, Stephanie Smits, Rubina Ahmed, Hilary Webb, Aziz Sheikh, Sally Cox, Ceri Bygrave, Julia Hippisley-Cox

**Affiliations:** 1 Cancer Division, Leeds Institute of Clinical Trials Research, University of Leeds, Leeds, UK; 2 Digital Health and Care Wales, Cardiff, UK; 3 Observatory and Cancer Analytical Team, Public Health Wales, Cardiff, UK; 4 Welsh Cancer Intelligence and Surveillance Unit, Public Health Wales, Cardiff, UK; 5 Blood Cancer Wales, Cardiff, UK; 6 Primary Care Epidemiology Group, Nuffield Department of Primary Care Health Sciences, University of Oxford, Oxford, UK; 7 University Hospital of Wales, Cardiff, UK; 8 Queen Mary University of London, London, UK

## Abstract

**Background:**

Blood cancers are among the most common and deadliest in the UK, affecting over 40,000 people annually. Survival varies by subtype, but this is not routinely reported for the UK. This study estimated survival for haematological malignancies in the UK stratified by time period, age, sex, ethnicity, deprivation, and rurality.

**Methods:**

Four retrospective cohorts of patients aged 15–99 diagnosed with haematological malignancies (2009–2019) across UK cancer registries were used to estimate 1-, 5-, and 10-year net survival by subtype.

**Results:**

We identified 413,286 blood cancer cases. Survival in all blood cancer combined significantly improved in England (3.8%), Northern Ireland (5.1%), and Wales (3%), but not Scotland. Men had ≥3% lower survival than women in many subtypes. Older age and higher deprivation were significantly linked to lower survival. In England, white ethnic groups had ≥3% lower survival than non-white groups for myelodysplastic syndrome, myeloid malignancies, plasma cell neoplasms, myeloproliferative neoplasms. In Wales, rural areas showed ≥3% higher survival than urban and mixed regions for lymphoid malignancies, myeloid malignancies, plasma cell neoplasms, Hodgkin lymphoma, mature B-cell neoplasms, acute myeloid leukaemia.

**Conclusion:**

Net survival varied markedly by subtype and demographic factors across the UK. Given possible differences in case ascertainment, findings are descriptive and hypothesis‑generating.

## Introduction

Blood cancers are among the most commonly diagnosed cancer types and rank as one of the leading causes of cancer-related deaths in the UK [1], affecting over 40,000 people each year [2]. Blood cancer survival varies significantly by subtype. For example, population‑based European registry studies report substantial variation in 5‑year survival, with estimates around 50–55% for myeloma and leukaemia, and over 80% for Hodgkin lymphoma [3]. Prior research has focused on reporting survival for specific groups, namely lymphoid neoplasms [4] and myeloid malignancies [5]. Additionally, routine national reporting typically only focuses on high-level categories i.e. HL, myeloid leukaemia, non-HL, limiting insights into more granular patterns [6]. Although survival for blood cancers has improved over recent decades [3], there have been limited analyses of differences in survival from blood cancers by factors such as age, sex, ethnicity, region and deprivation within the UK. Evidence from other countries and cancer types suggests that disparities likely exist [3, 7–9]. Consistent with this, evidence from the UK also demonstrates socioeconomic and disease-specific differences in cancer survival, including in haematological malignancies [10–13].

Large-scale, population-based information on the survival of patients from all hematologic malignancies (HM) according to the World Health Organization (WHO) Classification, the International Classification of Diseases for Oncology (ICD-O), or HAEMACARE groupings, remains scarce and is not routinely reported within the UK nations. The HAEMACARE groupings, a European initiative, grounded in the WHO classification/ICD-O-3, aimed to harmonise and improve the comparability of population-based data on HM across registries [14]. These groupings align with efforts by the International Lymphoma Epidemiology Consortium for lymphoid neoplasms and remain consistent with WHO principles [15].

Although the HAEMACARE classification is based on earlier WHO and ICD-O-3 standards, several updates have since been published, including the 4th edition (2008) [16], its revision (2016) [17], and the 5th edition (2022) [18], introducing new entities and refined criteria. However, during the period (2009–2019), many registries lacked access to the detailed pathology data needed to apply these newer systems [19]. HAEMACARE offered a practical, prognosis-based framework aligned with WHO and ICD-O-3, making it a suitable choice for consistent population-level analysis across European registries [19].

Moreover, examining survival of individual subtypes is crucial for both clinicians, researchers, policymakers and patients, including (1): developing personalised treatment plans (2); setting expectations and planning long-term care (3); identifying areas where more research and development of treatments needs to be prioritized (4); and informing deliberations on allocating healthcare resources.

We sought to examine survival patterns in the general population of patients diagnosed with all HM between 2009 and 2019 in the UK to identify disparities in survival between socio-demographic groups. To the best of our knowledge, this is the first time that population-based survival patterns for all HM classified into the 25 HEAMACARE groupings in all UK nations have been reported.

Survival estimates for HM are sensitive to the completeness of case ascertainment and underlying incidence patterns. In particular, myeloproliferative neoplasms (MPN) and myelodysplastic syndromes (MDS) have historically been under-registered due to legacy ICD-10 D-code categorisation, which can result in incomplete capture of these entities in population cancer registries. Epidemiologic best practice recommends interpreting survival alongside completeness assessments; however, given the scope and data permissions of this study, we present descriptive survival estimates and explicitly outline where potential incompleteness may influence interpretation.

## Methods

### Study design

Four retrospective cohort studies were undertaken using routinely collected data, including all patients aged 15–99 years old with HM diagnosed between Jan 1, 2009, and Dec 31, 2019.

### Participants

We included patients with an ICD-10 tumour code for HM (S1 Table) between Jan 1, 2009, and Dec 31, 2019 in the National Cancer Registration and Analysis Service (NCRAS), Scottish and Welsh cancer registry data. For Northern Ireland, cases were included based on ICD-O-3 histology codes for the internationally agreed 25 HEAMACARE groups (S2 Table) for adults belonging to ICD-10 tumour sites for blood cancers.

Once a nation’s cohort was selected, we grouped all HM by ICD-O-3 histology codes according to the HAEMACARE classification [14]. In some cases, histology codes from the cancer registry were not included in the HAEMACARE classification. We then mapped the codes to ICD-O-3 codes using the Surveillance, Epidemiology and End Results Hematopoietic and Lymphoid neoplasm database. In nations, we restricted analyses to HM with non-missing histology codes. Patients with non-specific histology codes (e.g. 8000, 8010, or 8800) comprised a small minority of cases in England (0.1%), NI (0%), Scotland (0.06%) and Wales (0.02%) and were excluded. Approximately 2% of patients had multiple haematological malignancy diagnoses. Because our analyses were diagnosis‑based, each diagnosis was counted independently.

### Variables of interest

Socio-demographic factors were included in our analysis, including five age groups (15–44; 45–54; 55–64; 65–74; 75–99), sex, time period (2009–2014 and 2015–2019), deprivation, ethnicity and rurality where possible.

#### Deprivation

Deprivation was measured using the Index of Multiple Deprivation (IMD) although its precise definition varied by each country. England, NI, Scotland and Wales each use their own IMD [20–23]. The specific domains and data sources used to develop the IMD vary between UK nations, making direct comparisons based on these indices impossible, as they omit the deprivation gradients between nations [24]. Specifically, the English IMD comprises seven domains with the following weightings: income (22.5%), employment (22.5%), education (13.5%), health and disability (13.5%), crime (9.3%), barriers to housing and services (9.3%), and living environment (9.3%). Health indicators are incorporated within the health and disability domain. In comparison, the Scottish IMD assigns greater weight to income and employment (28% each) and uses different health indicators, while Wales and Northern Ireland apply their own weighting schemes and data sources for domains such as housing, access to services, and community safety. Whilst there were some variations in how the quintiles were calculated, these have been extensively used to study deprivation gradients across the UK. We stratified by deprivation using quintiles: 1 represents the 20% most deprived areas, and 5 represents the 20% least deprived areas within a given nation. Due to the differences in how each nation calculates IMD, any one quintile group should not be considered equivalent *between* nations. That is to say that the 20% most deprived areas in the individual nations should not be compared to one another, but to the other quintiles within that nation.

#### Ethnicity

In England, the cancer registry uses the 16 + 1 ethnic data categories defined in the 2001 census. To facilitate analyses in less common cancers, we collapsed ethnicity into six categories (white, mixed, Asian, black, others and unknown) and four categories (white, non-white, others & unknown) consistent with previous publications [25, 26]. No ethnicity data were available for Northern Ireland. In Scotland, ethnicity data were provided but these were of insufficient quality to report (32.8% classified as unknown, and <1% classified as non-missing and non-white). Similarly, in Wales, there were ethnicity data available, and only 4.8% of ethnicities were unknown. However, a further 93% were white. This meant that the statistical stability criteria were never satisfied for non-white persons due to very small sample sizes, making the data unsuitable for analysis.

#### Rurality

In England, only aggregated national-level data were available within the linked datasets, in accordance with strict data governance and confidentiality requirements. In Scotland, data were available on rurality according to eight urban-rural classification codes, which we collapsed to align with the ‘UR 3-fold class’ used by the Scottish government [27]. In Northern Ireland, we stratified by rurality, defined as urban, mixed, or rural, and based on the 2015 Super Output Area [28]. In Wales, we used the 2011 Rural-Urban Classification based on the Lower Super Output Area (2011) with six urban-rural classification codes, which we collapsed into three groups (urban, mixed, and rural).

### Data sources

In England, we accessed cancer registry data through QResearch, which includes NCRAS dataset, Office for National Statistics (ONS) mortality, and a linked primary care database, among other data sources. Linkage of NCRAS and ONS data was performed at a patient level by the QResearch team. QResearch is an in-house version of the latest NCRAS dataset, which includes all cancer cases registered in England. Importantly, QResearch provides timely access to rich, linked data across cancer registries, mortality records and primary care records, enabling detailed analysis within the study timeframe. We used the Scottish Cancer Registry, the NICR and WCISU for the three remaining nations. All Welsh cancer registry data was accessed and analysed through the Secure Anonymised Information Linkage (SAIL) databank (Project ID 1642) [29–33], with linkage to ONS mortality and the Akbari et al. multi-source ethnicity spline carried out by the research team within SAIL [34].

### Statistical analyses

We estimated 1-, 5- and 10-year net survival using the Pohar-Perme estimator to account for informative censoring bias [35]. Net survival estimates were calculated by comparing the survival of cancer patients with that expected based on the background mortality of the general population with the same distribution of factors. In this study, background mortality was derived from population life tables publicly available for each individual nation. All life tables accounted for age, sex, nation, and calendar time. Life tables were adjusted for deprivation for England, but not for NI, Scotland, and Wales. The features and sources of the life tables are provided in S3 Table [36–38].

Follow-up time was defined as the time from when a patient was diagnosed with blood cancer until the first of their date of death, the end of follow-up, or the date of last known vital status according to the cancer registry. The end of follow-up was the Dec 31, 2022 for England, Scotland and Wales, and Dec 31, 2021 for NI.

Net survival estimates were age-standardised according to the International Cancer Survival Standard weights [39]. In order to produce statistically stable estimates, all age groups had to pass the statistical tests published by the NCRAS (S4 Table) [40].

Analysis was conducted for each nation separately due to their cancer registry data being stored on four different research servers as required by data providers. All analyses were performed in Stata version 18.0 by using the strs function [41]. Only outputs from groups with numbers ≥5 were accepted to report for England, Northern Ireland, and Scotland, ≥10 for Wales. A ‘significant’ difference was defined as non-overlapping net-survival confidence intervals.

### Assessment of completeness and denominators

Interpretation of survival estimates depends on the completeness of case registration and the accuracy of population denominators. For this analysis, we were unable to conduct subgrouped incidence analyses or formal completeness assessments across the four nations. Consistent with national cancer registration practice, certain myeloid subtypes (e.g. MPN and MDS) have historically been at higher risk of under‑ascertainment, partly due to legacy ICD‑10 D‑code usage. Survival estimates for these subtypes are therefore presented descriptively, and potential differential completeness by age, sex, deprivation, or ethnicity should be considered when interpreting subgroup comparisons.

## Results

We identified 342,169 blood cancer cases in England, 10,440 in Northern Ireland, 38,127 in Scotland, and 22,550 in Wales diagnosed between Jan 1, 2009, and Dec 31, 2019, aged 15–99. Numbers of cases and 5-year age-standardised net survival estimates for the 25 HAEMACARE groups and higher subtypes are shown in Table 1. The distribution of cases for all blood cancer combined by socio-demographic characteristics is presented in Table 2. The estimates for each subtype are presented in supplementary materials (S5 to S11 Tables).

**Table 1:** Cases (%) and Age-standardised 5-year net survival of HM by HAEMACARE categories for adults diagnosed in the period 2009 to 2019 in four nations

Blood cancer subtype				England				Northern Ireland				Scotland					Wales			
				*n*	%	5-yr survival	95%CI	*n*	%	5-year survival	95%CI	*n*	%	5-year survival	95%CI	*n*		%	5-year survival	95%CI
**All blood cancer**				342,169	100.0	62.3	(62.1, 62.5)	10,440	100	63.6	(62.4, 64.7)	38,127	100.0	65.0	(64.4, 65.6)	22,550		100.0	60.2	(59.4, 61.2)
**All lymphoid malignancies**				244,666	71.5	67.7	(67.5, 67.9)	7335	70.2	70.0	(68.5, 71.3)	23,292	61.1	67.3	(66.6, 68.0)	13,920		61.7	65.4	(64.4, 66.4)
Composite Hodgkin and non-Hodgkin Lymphoma				215	0.1	62.8	(53.6, 70.6)	6	0.1							<20				
**Hodgkin lymphoma (HL)**				18,011	5.3	85.4	(84.9, 85.8)	628	6.0	85.8	(82.8, 88.4)	1822	4.8	84.0	(82.3, 85.5)	980		4.3	82.7	(80.4, 84.7)
Hodgkin lymphoma, nodular lymphocyte predominance				1756	0.5	95.4	(93.6, 96.7)	84	0.8	92.6	(84.5, 96.6)	164	0.4	96.1	(75.1, 99.4)	100		0.4	93.4	(81.8, 97.7)
Classical HL				16,255	4.8	84.4	(83.8, 84.9)	544	5.2	84.9	(81.6, 87.7)	1658	4.3	82.7	(81.0, 84.3)	880		3.9	81.3	(79.0, 83.4)
**Mature B-cell neoplasms (MBN)**				193,702	56.6	69.5	(69.2, 69.7)	5659	54.2	72.5	(70.9, 74.0)	18,669	49.0	69.9	(69.1, 70.7)	11,110		49.3	67.6	(66.5, 68.7)
Chronic lymphocytic leukaemia/Small lymphocytic lymphoma				40,900	12.0	82.1	(81.5, 82.6)	1226	11.8	87.5	(83.9, 90.4)	3641	9.5	84.9	(83.0, 86.6)	2610		11.6	82.4	(80.0, 84.5)
Immunoproliferative diseases				7419	2.2	82.5	(81.2, 83.7)	162	1.6			702	1.8	78.2	(73.6, 82.1)	370		1.6	75.0	(69.0, 80.0)
Mantle cell/ centrocytic				5558	1.6	57.6	(56.1, 59.2)	143	1.4	54.2	(44.1, 63.3)	545	1.4	58.9	(54.1, 63.5)	350		1.6	55.5	(49.1, 61.5)
Follicular lymphoma				23,322	6.8	84.3	(83.6, 85.0)	843	8.1	84.4	(80.0, 87.9)	2485	6.5	85.5	(83.1, 87.6)	1240		5.5	80.4	(76.8, 83.5)
Diffuse large B-cell lymphoma (DLBCL)				47,963	14.0	61.0	(60.5, 61.5)	1394	13.3	64.3	(61.1, 67.3)	4869	12.8	63.6	(62.0, 65.3)	2840		12.6	60.0	(57.8, 62.1)
Burkitt’s				1647	0.5	44.1	(41.0, 47.1)	33	0.3			208	0.5	52.6	(44.5, 60.0)	80		0.4		
Marginal zone lymphoma				12,412	3.6	85.9	(84.9, 86.8)	260	2.5	92.2	(82.7, 96.6)	1102	2.9	86.8	(83.5, 89.5)	600		2.7	82.5	(76.8, 86.9)
Plasma cells neoplasms (Myeloma)				52,272	15.3	56.1	(55.6, 56.6)	1534	14.7	57.8	(54.6, 60.9)	4981	13.1	54.0	(52.3, 55.7)	2920		12.9	54.9	(52.7, 56.9)
Mature B cell leukaemia				72	0.0	50.7	(36.0, 63.7)									<20				
Mature B cell leukaemia, hairy cell				2137	0.6	89.4	(86.6, 91.6)	56	0.5							110		0.5	79.3	(65.9, 87.9)
**Mature T-cell and NK-cell neoplasms (MTNKN)**				10,980	3.2	53.1	(51.9, 54.3)	320	3.1	43.9	(37.4, 50.2)	1189	3.1	50.1	(46.7, 53.4)	720		3.2	48.1	(43.6, 52.4)
T lymphoma cutaneous				3783	1.1	77.8	(75.7, 79.8)	71	0.7	77.3	(58.9, 88.3)	372	1.0	72.8	(65.0, 79.1)	170		0.8	75.3	(65.7, 82.6)
Other T cell lymphomas				7197	2.1	40.5	(39.2, 41.8)	249	2.4	35.8	(29.0, 42.6)	817	2.1	39.9	(36.1, 43.6)	340		1.5	39.6	(33.7, 45.4)
**Lymphoblastic lymphoma/Acute (precursor cell) lymphatic leukaemia (ALL)**				3761	1.1	51.7	(50.1, 53.2)	252	2.4			359	0.9	50.3	(45.1, 55.3)	210		0.9	58.1	(51.8, 63.8)
**Unknown lymphoid neoplasms**				17,997	5.3	59.4	(58.5, 60.2)	470	4.5	59.9	(54.3, 65.0)	1238	3.2	49.6	(46.2, 52.9)	1120		5.0	50.9	(47.1, 54.6)
Lymphoma, NOS				5013	1.5	49.8	(48.1, 51.4)	247	2.4	45.8	(38.0, 53.3)	473	1.2	25.8	(20.2, 31.8)	340		1.5		
NHL, NOS				12,340	3.6	62.9	(61.8, 63.9)	217	2.1	73.4	(64.8, 80.2)	751	2.0	60.4	(56.2, 64.3)	730		3.2	55.0	(50.5, 59.2)
Lymphatic leukaemia, NOS				644	0.2	65.2	(60.6, 69.5)	6	0.1							60		0.3		
**All Myeloid malignancies**				95,571	27.9	48.1	(47.7, 48.5)	2979	28.4	47.8	(45.6, 50.0)	11,827	31.0	56.4	(55.3, 57.4)	8240		36.5	50.5	(49.2, 51.8)
Acute myeloid leukaemia (AML)				27,882	8.1	22.5	(22.0, 23.0)	775	7.4	23.0	(19.9, 26.2)	2450	6.4	21.7	(20.0, 23.4)	1920		8.5	23.2	(20.2, 24.3)
Myeloproliferative neoplasms (MPN)				30,312	8.9	77.9	(77.2, 78.5)	985	9.4	74.5	(70.5, 78.0)	4779	12.5	82.5	(80.9, 84.0)	3390		15.0	73.4	(71.2, 75.4)
Myelodysplastic syndrome (MDS)				28,376	8.3	40.7	(39.8, 41.5)	980	9.4	40.0	(35.4, 44.5)	3661	9.6	44.8	(42.3, 47.3)	2440		10.8	38.0	(34.7, 41.2)
Myelodysplastic/Myeloproliferative neoplasms (MDN)				6830	2.0	44.1	(42.5, 45.6)	108	1.0			860	2.3	47.4	(42.8, 51.9)	360		1.6	24.5	(16.9, 32.9)
Unknown myeloid neoplasms				2171	0.6	38.4	(35.8, 41.0)	121	1.2	43.4	(30.5, 55.6)	77	0.2			130		0.6	50.9	(47.1, 54.6)
Leukaemia, NOS				1305	0.4	35.6	(32.4, 38.8)	91	0.9	48.2	(33.8, 61.2)					100		0.4		
Myeloid leukaemia, NOS				866	0.3	43.0	(38.7, 47.2)	30	0.3							30		0.1		
**Others**				1932	0.6	65.2	(62.3, 68.0)	140	1.3	70.0	(57.9, 79.2)	3008	7.9	80.1	(78.0, 82.0)	390		1.7	69.0	(62.7, 74.5)

**Table 2:** Cases and (%) of all blood cancer diagnosed in the period 2009 to 2019, by demographic characteristics in four nations

Demographic chacteristics	England		Northern Ireland		Scotland		Wales	
	*n* = 342,169		*n* = 10,440		*n* = 38,127		*n* = 22,550	
	n	%	*n*	%	*n*	%	*n*	%
Time period								
2009–2014	176,892	51.7	5246	50.2	20,592	54.0	12,745	56.5
2015–2019	165,277	48.3	5194	49.8	17,535	46.0	9805	43.5
Age groups								
15–44	28,816	8.4	1118	10.7	2860	7.5	1620	7.2
45–54	28,182	8.2	928	8.9	3142	8.2	1745	7.7
55–64	53,568	15.7	1689	16.2	6380	16.7	3615	16.0
66–74	91,519	26.7	2772	26.6	10,560	27.7	6280	27.8
75–99	140,084	40.9	3933	37.7	15,185	39.8	9290	41.2
Sex								
Male	194,206	56.8	5800	55.6	20,922	54.9	12,780	56.7
Female	147,963	43.2	4640	44.4	17,205	45.1	9770	43.3
Deprivation								
5-Least deprived	76,045	76,045.0	2216	21.2	7196	18.9	4710	20.9
4	74,970	21.9	2195	21.0	7917	20.8	4875	21.6
3	71,865	21.0	2115	20.3	8172	21.4	4725	21.0
2	63,297	18.5	2154	20.6	7452	19.5	4405	19.5
1-Most deprived	55,992	16.4	1759	16.8	7390	19.4	3835	17.0
Ethnicity								
White	302,772	88.5	–	–	–	–	–	–
Mixed	1648	0.5	–	–	–	–	–	–
Asian	11,490	3.4	–	–	–	–	–	–
Black	7507	2.2	–	–	–	–	–	–
Others	4300	1.3	–	–	–	–	–	–
Unknown	9721	2.8	–	–	–	–	–	–
Missing	4731	1.4	–	–	–	–	–	–
Rurality								
Urban	–	–	6063	58.1	30,554	80.1	14,575	64.6
Mixed	–	–	935	9.0	4869	12.8	4125	18.3
Rural	–	–	3441	33.0	2704	7.1	3850	17.1

### Overall

**Fig. 1:**
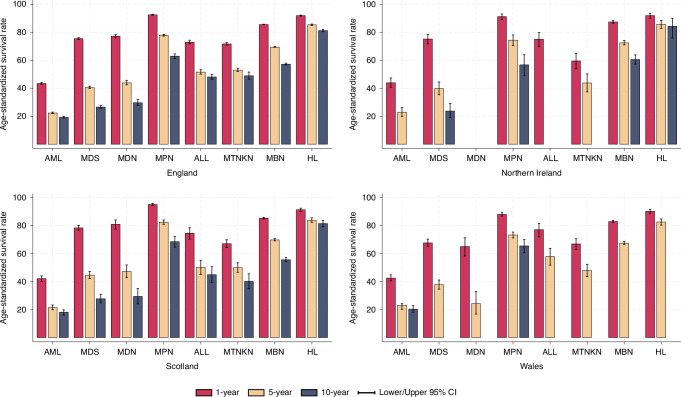
Age-standardised 1-year, 5-year and 10-year net survival (%) for adults diagnosed in the period 2009 to 2019 in four nations, by 8 higher HAEMACARE groups.

In England, age-standardised net survival for all blood cancers was 80.1%, 95% CI:80.0–80.3 (1-year), 62.3%, 62.1–62.5 (5-year), and 51.3%, 50.8–51.7 (10-year). In Northern Ireland, it was 80.9% (80.1–81.7), 63.6% (62.4–64.7), and 52.5% (50.4–54.7), respectively. In Scotland, 1-, 5- and 10- year net survival was 82.3% (81.9–82.7), 65.0% (64.4–65.6), and 51.8% (50.8–52.8), respectively. In Wales, it was 76.8% (72.2–77.4) and 60.2% (59.4–61.2); 10-year survival did not pass the statistical stability criteria (see Table S4). The highest 5-year survival was for Hodgkin lymphoma with nodular lymphocyte predominance (England: 95.4% (93.6–96.7); Northern Ireland: 92.6% (84.5–96.6); Scotland: 97.0% (87.2–99.3); Wales: 93.4% (81.8–97.7)). The lowest 5-year survival was for AML (England: 22.5% (22.0–23.0); NI: 23.0% (19.9–26.2); Scotland: 21.7% (20.0–23.4); Wales: 23.2% (20.2–24.3)). Figure 1 shows 1-year, 5-year and 10-year survival patterns for eight higher HAEMACARE groups, including four lymphoid malignancies (HL, MBN, MTNKN and ALL) and four myeloid malignancies (MPN, MDN, MDS, AML) (supplementary S5 Table for all groups). Overall, 1-year, 5-year and 10-year net survival is similar and consistent across all nations for AML, MDS, ALL, MBN and HL.

### Time period

HM survival increased over the study period in the ‘all blood cancer’ group and in most specific subtypes and groups across all nations (supplementary S6 Table for all groups). The increased survival in all blood cancer combined was statistically significant in England (3.8%), Northern Ireland (5.1%) and Wales (3%), but not in Scotland. Noticeably, plasma cells neoplasms and NHL (non‑Hodgkin lymphoma) showed marked survival improvements with over 7% in all four nations. For plasma cell neoplasms specifically, survival increased between the two study periods from 52.8% to 59.8% in England, 52.3% to 63.0% in Northern Ireland, 50.4% to 58.1% in Scotland, and 51.6% to 58.6% in Wales. Similarly, for NHL, survival rose from 60.6% to 65.4% in England, 64.5% to 78.7% in Northern Ireland, 56.7% to 63.8% in Scotland, and 51.5% to 59.4% in Wales. Furthermore, a considerable increase in survival of mantle cell leukaemia and HL (all) was identified in England, Scotland and Wales. Survival declined by over 3% in some subtypes, though overlapping confidence intervals suggest these changes were not statistically significant and likely reflect evolving coding practices, where fewer cases are classified as ‘not otherwise specified (NOS)’ and those remaining tend to have poorer prognosis.

### Age groups

In general, there were significant negative relationships between age and net-survival across most HAEMACARE groups in all four nations (Supplementary S7 Table for all groups). In particular, the largest differences in net survival between ages 15–44 and 65–74 were for AML, ALL, all myeloid malignancies, classical HL, all lymphoid malignancies, and diffuse B lymphoma (Supplementary Fig. S1). The top five subtypes and broader groups showing the greatest variation in 5-year survival by age were broadly consistent across all four UK nations. For England, the largest distinction in 5-year survival between ages 15–44 and 65–74 was for MDN with a survival difference of 56%, and the smallest gradient was for hairy cell leukaemia (3.6%). For Northern Ireland, AML had a 5-year net survival of 65.7% (95%CI: 56.3–73.5) among 15–44-year-olds, but among 65–74-year-olds it was 12.3% (95%CI: 7.9–17.8). In Scotland, there was a difference in 5-year survival of 59% between the same age groups for HL. For Wales, the largest difference of 45% was for classical HL, while the smallest difference of 21.7% was for follicular lymphoma.

### Sex

Overall, age-standardised 5-year survival rates were generally higher in females than males for blood cancers; subtypes and higher groups with ≥3% difference in net survival (Supplementary Fig. S2). In England, survival difference by sex was consistent across nearly all subtypes, but the magnitude of it varied (see Supplementary S8 Table for all groups). Notably, significant sex-based survival disparities (≥3%) were found in 15 HAEMACARE groups. In England, the greatest survival disadvantages for males were for MDN (10%), MPN (9.2%), all myeloid malignancies (8.3%) and mantle cell lymphomas (6.2%). Statistically significant differences for Northern Ireland, Scotland and Wales were identified for MPN and all myeloid malignancies with survival disadvantages for males.

### Deprivation

**Fig. 2:**
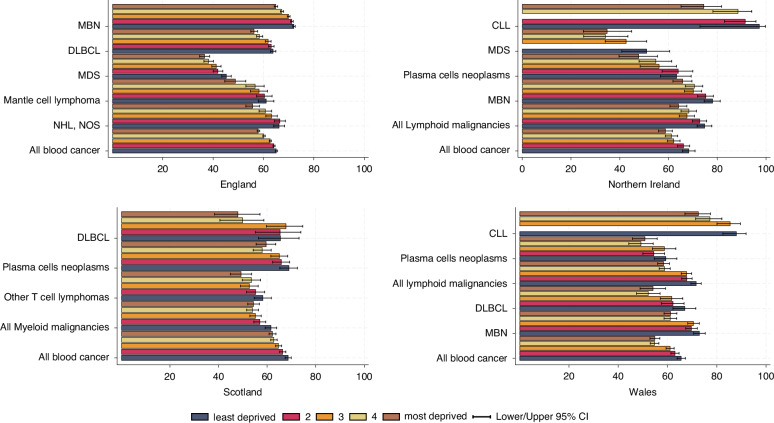
Age-standardised 5-year net survival (%) for adults diagnosed in the period 2009 to 2019 in the UK, and the site-specific variation in survival difference by deprivation for HAEMACARE groups.

Five-year age-standardised net survival for all blood cancers combined was consistently higher in the least deprived quintile compared to the most deprived in all 4 nations: England (65.2% [95%CI: 64.8–65.5] vs. 58.0% [57.5–58.5]), NI (68.4% [65.7–70.9] vs. 58.8% [55.9–61.6]), Scotland (68.7% [67.4–69.9] vs. 62.3% [60.8–63.7]), and Wales (65.6% [63.9–67.3] vs. 54.9% [52.9–56.9]). HAEMACARE groups with the most significant survival disparities between the most and least deprived quintiles were MBN, DLBCL, and all lymphoid malignancies. Figure 2 shows subtypes with survival variation ≥ 7% between the least and the most derived quintile, which were generally consistent across nations. A gradient in net survival from least to most deprived was presented for most HAEMACARE groups in England, but usually only statistically significant when comparing the 20% least and 20% most deprived quintiles. In Northern Ireland, survival differences by deprivation quintile were statistically significant for all lymphoid malignancies combined (74.9% [95%CI: 71.7–77.8] vs. 64.2% [60.6–67.5]), MBN (78.1% [74.6–81.2] vs. 65.9% [61.8–69.6]), and plasma cell neoplasms (63.4% [56.7–69.3] vs. 47.8% [39.7–55.5]). In Scotland, survival varied by deprivation for both lymphoid and myeloid malignancies: lymphoid malignancies combined (70.7% [69.0–72.2] vs. 64.2% [62.3–66.0]) and myeloid malignancies (61.6% [59.2–64.0] vs. 54.5% [52.0–56.8]). Overall, survival in the least deprived group was approximately 1.1 times higher than in the most deprived group In Wales, significant differences were observed for CLL (88.0% [82.5–91.9] vs. 72.6% [67.1–77.4]), all lymphoid malignancies combined (71.7% [69.5–73.7] vs. 58.5% [56.0–60.8]), and marginal zone lymphoma (91.3% [75.8–97.1] vs. 78.1% [64.4–87.1]). Detailed results for all groups are provided in Supplementary Table S9.

### Ethnicity

Ethnicity estimated net survival analysis was carried out exclusively on English data (see Methods). Findings are presented as exploratory analyses and detailed in the supplementary S10a and S10b Tables for full results. Overall, white people had poorer age-standardised 5-year survival than other ethnic groups in the majority of subtypes. We identified subgroups with over 3% of difference between non-white/white, mixed/white, Asian/white and black/white and present them in Supplementary Fig. S3. Specifically, survival disparities of ≥ 3% between white and non-white were found in follicular lymphoma, MDS, all myeloid malignancies, plasma cells neoplasms, MPN and MDN. The mixed ethnic group was 5.4% higher than white for survival of all blood cancer combined. Additionally, Asian and black people had statistically significantly better survival than white in MPN, plasma cells neoplasms, all myeloid malignancies.

### Rurality

Overall, there were no statistically significant differences in age-standardised net 5-year survival by rurality in NI and Scotland. However, significant survival distinctions by rurality were identified in Wales for all blood cancers combined, plasma cells neoplasms, Hodgkin lymphoma (all), MBN, all lymphoid malignancies, all myeloid malignancies, and AML, with higher survival in rural vs urban and mixed regions. Findings are presented as exploratory analyses and detailed in the Supplementary Figs. S4 and S11 Tables for full results.

## Discussion

To our knowledge, this is the first study to report on disparities in net-survival for HAEMACARE groups by age, sex, ethnicity, deprivation and rurality between the four UK nations. We report significant disparities with poorer survival in men compared with women, in white compared with non-white ethic groups, for patients from deprived areas compared with those from less deprived areas. Whilst blood cancer survival improved modestly over the study period in three of the four UK nations, it was difficult to identify statistically significant changes for rare subtypes. Increases were observed for plasma cell neoplasms and mantle cell lymphomas. These changes are consistent with recent improvements in treatment linked to approvals of new treatments via NICE and improved classification of HM. Furthermore, our findings align with other UK studies that had access to treatment data and reported similar increases in survival for these subtypes [42, 43]. However, the relative increase remains lower than in similarly developed nations according to international comparison studies such as International Cancer Benchmarking Partnership (ICBP) [44], EUROCARE-5(3) and CONCORD-3 International Cancer [45]. In our study, several subtypes showed little to no survival change, which might be down to various factors, such as delayed access to diagnosis and treatment, aging populations, increasingly prevalent socioeconomic challenges, and changes in coding. Therefore, further investigation into what is driving increased survival in some subtypes over time but not others is needed. There are clearly steep age gradients in survival from HM, and these have also been observed in prior research [3, 46]. These differences in survival likely stem from variation in the underlying biology of the disease by age. Regarding sex, higher survival rates among females compared to males were observed for most subtypes. This is consistent with prior research [46, 47] and hypothesised to be at least in part due to differences in comorbidities, behavioural factors, and biological factors [48, 49]. With regards to deprivation, statistically significant differences between the most and least deprived quintiles were determined for most HM, but the gradients through the quintiles were not always consistent. Statistically significant differences were difficult to discern at the subtype-level, especially in NI, Scotland and Wales. In Wales, the most notable survival differences by rurality were observed for all blood cancers combined, plasma cell neoplasms, HL, MBN, all lymphoid malignancies, all myeloid malignancies, and AML. However, there were no significant differences in 5-year net survival by rurality in NI and Scotland.

Ethnicity‑stratified survival estimates are presented as exploratory, descriptive findings. Non-white people had higher survival estimates than white in the majority of subtypes in which over 3% of survival disparities were found in follicular lymphoma, MDS, all myeloid malignancies, plasma cells neoplasms, MPN and MDN. Additionally, Asian and black people had higher survival than white in MPN, MDS, plasma cells neoplasms, all myeloid malignancies. These are novel findings rarely seen in malignancy, suggestive of disease heterogeneity by ethnicity, and are not routinely reported. Additionally, prior research shows higher multiple myeloma survival rates in black adults compared to white adults, with no data on other hematologic malignancies [50]. This may be in part due to the life tables which are used to estimate net survival and do not account for ethnicity in the background (expected) mortality. However, this might be expected to bias the results toward overestimating survival among white persons, which would not explain these findings [51]. Furthermore, ethnic associations in blood cancer outcomes are likely multifactorial, potentially influenced by factors such as age at diagnosis, disease subtype, access to care, and treatment pathways, highlighting the value of holistic data collection and analysis. For instance, non-white individuals diagnosed with myeloma tend to be younger [52] and are more likely to present with smoldering myeloma [53], which can significantly impact survival. These complexities highlight the need for caution when interpreting ethnic differences. In particular, the universal and systematic collection of genomic data through national reporting could support deeper investigation into these patterns, while acknowledging that genomic information alone may not fully explain observed disparities. Importantly, ethnic differences in incidence rates remain central to understanding ethnic disparities [54, 55]. UK and international studies consistently report higher myeloma incidence among individuals of African ancestry, which may reflect underlying biological differences, disease presentation, and treatment response [56, 57]. The absence of staging and treatment data in our study limits our ability to fully account for clinical factors that may influence survival outcomes across ethnic groups.

This study has several methodological strengths. It is population-based, drawing data from cancer registries with national coverage, and stratifies by a broad set of demographic factors. Although we were able to present net survival estimates for the majority of factors across the majority of nations, the authors recommend against comparing findings across nations due to differences in data sources, stratification variables, follow-up times, and life tables. Small case numbers for some subtypes lead to unstable estimates. Additionally, variations in cancer registry coding may affect survival comparisons between nations.

Interpretation of subgroup survival depends on accurate denominators and complete case registration. Certain myeloid subtypes, particularly MPN and MDS, have historically been susceptible to under‑registration in population datasets, including legacy ICD‑10 D‑code usage. We were unable to undertake incidence‑based completeness assessments for this revision. Observed survival differences especially by ethnicity, sex, age, and deprivation, should therefore be viewed as descriptive and potentially sensitive to differential completeness. We indicate where data availability or statistical‑stability thresholds constrained stratification, and these limitations should be considered when interpreting comparisons. Future work should prioritise incidence and completeness assessments to support more robust survival comparisons.

These findings informed Blood Cancer UK’s 2024 Action Plan [58], presented to the UK and devolved parliaments between September and October 2024 to promote policies for better blood cancer survival and to reduce disparities. The work also highlights the difficulty in obtaining comprehensive data of this kind from existing systems due to fragile IT infrastructure and poor interoperability between systems. Therefore, Blood Cancer UK’s report also included a call for national blood cancer data collected, analysed, and reported by UK cancer registries to be consistent and comparable, and also a call for blood cancer to be routinely recorded and reported as a distinct category alongside solid tumour. Furthermore, this evidence is crucial for monitoring current and shaping future national blood cancer plans and initiatives in England, NI, Scotland and Wales, and informing the NHS Outcomes Framework, enhancing NICE guidance for haematological cancers, and highlighting survival patterns by demographic factors to identify areas for improvement in early diagnosis.

Our population-based analyses of survival patterns in haematological malignancies offer useful insights for clinicians, patients, researchers, and policymakers. However, interpretation should be cautious because the absence of data on incidence, staging, and treatment limits the scope of inference. In addition, ethnicity shows marked regional patterning and is not comparable to age, sex, or deprivation; averaging data across the country may obscure important heterogeneity. The available ethnicity data are crude and self-reported, so a more nuanced approach is required to examine these patterns directly. Nevertheless, stratifying survival by ethnicity remains statistically meaningful for several reasons. Ethnicity captures biological, social, and access-related factors that influence survival, including genetic predispositions (e.g. certain myeloma subtypes more common in Black populations), socioeconomic conditions, healthcare access, and comorbidity profiles. The descriptive survival patterns may help prioritise future investigations and data improvements (e.g. completeness audits, linkage for stage/treatment), which are prerequisites to designing and evaluating targeted interventions. Other limitations include potential variation in diagnostic practices, incomplete registry data, and lack of information on comorbidities and socioeconomic factors that may influence survival.

In summary, we report national, population‑based descriptive net‑survival estimates across haematological malignancy subtypes and sociodemographic groups. Because completeness may vary particularly for some myeloid entities (e.g. MPN, MDS) and because stage, treatment, and comorbidity were unavailable, these findings should be interpreted with caution. We did not conduct incidence‑based completeness assessments across strata; observed subgroup differences (including by ethnicity, sex, age, deprivation, and rurality) are therefore exploratory and hypothesis‑generating rather than confirmatory.

We identified marked variation by subtype and across demographic and geographic groups. However, given data and methodological constraints, we refrain from causal interpretation and from drawing policy directives. Future work should first evaluate incidence and completeness and, where feasible, incorporate stage, treatment, and comorbidity data to determine whether observed patterns persist and to support more robust inference. Delayed or reduced access to appropriate therapies could plausibly have contributed to the observed geographical disparities between nations. Population-based survival by morphological subtype is important for measuring outcomes of HM continuing management. To better inform quality of care research, the collection of detailed clinical information at the population level should be prioritised.

## Supplementary information

Supplementary information

Appendix G - England cancer survival summary tables_v1.0

Appendix H - Wales cancer survival summary tables

Appendix I - Scotland cancer survival summary tables

Appendix K - N Ireland cancer survival summary tables

## Data Availability

Data cannot be shared publicly as they are analysed under licence. All data used in this study are available from QResearch (www.qresearch.org), the Northern Ireland, Scottish and Welsh cancer registries. Data sharing agreements for each nation was obtained.
