# Supplementary material for: Disparities in blood cancer survival in the UK 2009–2019: national cohort studies
Source: BJC Rep. 2026 Apr 15;4:19. doi: 10.1038/s44276-026-00222-0 (PMC13083837; doi:10.1038/s44276-026-00222-0)

A List of Supporting information

S1 Table: QResearch ICD10 code list for haematological malignancies

S2 Table: HAEMACARE group: the International Classification of Haematological malignancies-based ICD-O-3 histology codes

S3 Table: Summary of life table components, features, and sources

S4 Table: Statistical criteria for survival estimates by NCAS

S5 Table: 1-year, 5-year and 10-year age-standardised survival rates and 95% CI in four nations for Figure 1

S6 Table: 5-year age-standardised survival rates and 95% CI by periods in four nations

S7 Table: 5-year survival rates and 95% CI by age groups in four nations

S8 Table: 5-year age-standardised survival rates and 95% CI by sex in four nations

S9 Table: 5-year age-standardised survival rates and 95% CI by deprivation in four nations for Figure 2

S10a and S10b Table: 5-year age-standardised survival rates and 95% CI by ethnicity in England for Figure 3

S11 Table: 5-year age-standardised survival rates and 95% CI by rurality in Wales for Figure 4

Figure S1: 5-year net survival (%) for adults diagnosed in the period 2009 to 2019, and the site-specific variation in survival difference by age groups for HAEMACARE groups.

Figure S2: Age-standardised 5-year net survival (%) for adults diagnosed in the period 2009 to 2019, and the site-specific variation in survival difference by sex for HAEMACARE groups.

Figure S3: Age-standardised 5-year net survival (%) for adults diagnosed in the period 2009 to 2019 in England, and the site-specific variation in survival difference by ethnicity for HAEMACARE groups.

Figure S4: Age-standardised 5-year net survival (%) for adults diagnosed in the period 2009 to 2019 in Wales, and the site-specific variation in survival difference by rurality for HAEMACARE groups.

Appendix G: England cancer summary tables

Appendix K: Northern Ireland cancer survival summary tables

Appendix I: Scotland cancer survival summary tables

Appendix H: Wales cancer survival summary tables

S1 Table: QResearch ICD10 code list for haematological malignancies

ICD-10 code	ICD-10 code, labelled
B211	B211 - HIV disease resulting in Burkitt lymphoma
B212	B212 - HIV disease resulting in other types of non-Hodgkin lymphoma
B213	B213 - HIV disease resulting in other malignant neoplasms of lymphoid, haematopoietic and related tissue
C81	C81 - Hodgkin lymphoma
C810	C810 - Nodular lymphocyte predominant Hodgkin lymphoma
C811	C811 - Nodular sclerosis (classical) Hodgkin lymphoma
C812	C812 - Mixed cellularity (classical) Hodgkin lymphoma
C813	C813 - Lymphocyte depleted (classical) Hodgkin lymphoma
C814	C814 - Lymphocyte-rich (classical) Hodgkin lymphoma
C817	C817 - Other (classical) Hodgkin lymphoma
C819	C819 - Hodgkin lymphoma, unspecified
C82	C82 - Follicular lymphoma
C820	C820 - Follicular lymphoma grade I
C821	C821 - Follicular lymphoma grade II
C822	C822 - Follicular lymphoma grade III, unspecified
C823	C823 - Follicular lymphoma grade IIIa
C824	C824 - Follicular lymphoma grade IIIb
C825	C825 - Diffuse follicle centre lymphoma
C826	C826 - Cutaneous follicle centre lymphoma
C827	C827 - Other types of follicular lymphoma
C829	C829 - Follicular lymphoma, unspecified
C83	C83 - Non-follicular lymphoma
C830	C830 - Small cell B-cell lymphoma
C831	C831 - Mantle cell lymphoma
C833	C833 - Diffuse large B-cell lymphoma
C835	C835 - Lymphoblastic (diffuse) lymphoma
C837	C837 - Burkitt lymphoma
C838	C838 - Other non-follicular lymphoma
C839	C839 - Non-follicular (diffuse) lymphoma, unspecified
C84	C84 - Mature T/NK-cell lymphomas
C840	C840 - Mycosis fungoides
C841	C841 - SÃ©zary disease
C844	C844 - Peripheral T-cell lymphoma, not elsewhere classified
C845	C845 - Other mature T/NK-cell lymphomas
C846	C846 - Anaplastic large cell lymphoma, ALK-positive
C847	C847 - Anaplastic large cell lymphoma, ALK-negative
C848	C848 - Cutaneous T-cell lymphoma, unspecified
C849	C849 - Mature T/NK-cell lymphoma, unspecified
C85	C85 - Other and unspecified types of non-Hodgkin lymphoma

C851	C851 - B-cell lymphoma, unspecified
C852	C852 - Mediastinal (thymic) large B-cell lymphoma
C857	C857 - Other specified types of non-Hodgkin lymphoma
C859	C859 - Non-Hodgkin lymphoma, unspecified
C86	C86 - Other specified types of T/NK-cell lymphoma
C860	C860 - Extranodal NK/T-cell lymphoma, nasal type
C861	C861 - Hepatosplenic T-cell lymphoma
C862	C862 - Enteropathy-type (intestinal) T-cell lymphoma
C863	C863 - Subcutaneous panniculitis-like T-cell lymphoma
C864	C864 - Blastic NK-cell lymphoma
C865	C865 - Angioimmunoblastic T-cell lymphoma
C866	C866 - Primary cutaneous CD30-positive T-cell proliferations
C88	C88 - Malignant immunoproliferative diseases
C880	C880 - WaldenstrÃ¶m macroglobulinaemia
C882	C882 - Other heavy chain disease
C883	C883 - Immunoproliferative small intestinal disease
C884	C884 - Extranodal marginal zone B-cell lymphoma of mucosa-associated lymphoid tissue [MALT-lymphoma]
C887	C887 - Other malignant immunoproliferative diseases
C889	C889 - Malignant immunoproliferative disease, unspecified
C90	C90 - Multiple myeloma and malignant plasma cell neoplasms
C900	C900 - Multiple myeloma
C901	C901 - Plasma cell leukaemia
C902	C902 - Extramedullary plasmacytoma
C903	C903 - Solitary plasmacytoma
C91	C91 - Lymphoid leukaemia
C910	C910 - Acute lymphoblastic leukaemia [ALL]
C911	C911 - Chronic lymphocytic leukaemia of B-cell type
C913	C913 - Prolymphocytic leukaemia of B-cell type
C914	C914 - Hairy-cell leukaemia
C915	C915 - Adult T-cell lymphoma/leukaemia [HTLV-1-associated]
C916	C916 - Prolymphocytic leukaemia of T-cell type
C917	C917 - Other lymphoid leukaemia
C918	C918 - Mature B-cell leukaemia Burkitt-type
C919	C919 - Lymphoid leukaemia, unspecified
C92	C92 - Myeloid leukaemia
C920	C920 - Acute myeloblastic leukaemia [AML]
C921	C921 - Chronic myeloid leukaemia [CML], BCR/ABL-positive
C922	C922 - Atypical chronic myeloid leukaemia, BCR/ABL-negative
C923	C923 - Myeloid sarcoma
C924	C924 - Acute promyelocytic leukaemia [PML]
C925	C925 - Acute myelomonocytic leukaemia
C926	C926 - Acute myeloid leukaemia with 11q23-abnormality
C927	C927 - Other myeloid leukaemia
C928	C928 - Acute myeloid leukaemia with multilineage dysplasia

C929	C929 - Myeloid leukaemia, unspecified
C93	C93 - Monocytic leukaemia
C930	C930 - Acute monoblastic/monocytic leukaemia
C931	C931 - Chronic myelomonocytic leukaemia
C933	C933 - Juvenile myelomonocytic leukaemia
C937	C937 - Other monocytic leukaemia
C939	C939 - Monocytic leukaemia, unspecified
C94	C94 - Other leukaemias of specified cell type
C940	C940 - Acute erythroid leukaemia
C942	C942 - Acute megakaryoblastic leukaemia
C943	C943 - Mast cell leukaemia
C944	C944 - Acute panmyelosis with myelofibrosis
C946	C946 - Myelodysplastic and myeloproliferative disease, not elsewhere classified
C947	C947 - Other specified leukaemias
C95	C95 - Leukaemia of unspecified cell type
C950	C950 - Acute leukaemia of unspecified cell type
C951	C951 - Chronic leukaemia of unspecified cell type
C957	C957 - Other leukaemia of unspecified cell type
C959	C959 - Leukaemia, unspecified
C96	C96 - Other and unspecified malignant neoplasms of lymphoid, haematopoietic and related tissue
C960	C960 - Multifocal and multisystemic (disseminated) Langerhans-cell histiocytosis [Letterer-Siwe disease]
C962	C962 - Malignant mast cell tumour
C964	C964 - Sarcoma of dendritic cells (accessory cells)
C965	C965 - Multifocal and unisystemic Langerhans-cell histiocytosis
C966	C966 - Unifocal Langerhans-cell histiocytosis
C967	C967 - Other specified malignant neoplasms of lymphoid, haematopoietic and related tissue
C968	C968 - Histiocytic sarcoma
C969	C969 - Malignant neoplasm of lymphoid, haematopoietic and related tissue, unspecified
D45X	D45X - Polycythaemia vera
D46	D46 - Myelodysplastic syndromes
D460	D460 - Refractory anaemia without ring sideroblasts, so stated
D461	D461 - Refractory anaemia with ring sideroblasts
D462	D462 - Refractory anaemia with excess of blasts [RAEB]
D464	D464 - Refractory anaemia, unspecified
D465	D465 - Refractory anaemia with multi-lineage dysplasia
D466	D466 - Myelodysplastic syndrome with isolated del(5q) chromosomal abnormality
D467	D467 - Other myelodysplastic syndromes
D469	D469 - Myelodysplastic syndrome, unspecified
D47	D47 - Other neoplasms of uncertain or unknown behaviour of lymphoid, haematopoietic and related tissue

D471	D471 - Chronic myeloproliferative disease
D473	D473 - Essential (haemorrhagic) thrombocythaemia
D475	D475 - Chronic eosinophilic leukaemia [hypereosinophilic syndrome]
D477	D477 - Other specified neoplasms of uncertain or unknown behaviour of lymphoid, haematopoietic and related tissue
D479	D479 - Neoplasm of uncertain or unknown behaviour of lymphoid, haematopoietic and related tissue, unspecified
L412	L412 - Lymphomatoid papulosis
M820	M820 - Osteoporosis in multiple myelomatosis
Q822	Q822 - Mastocytosis

S2 Table: HAEMACARE group: the International Classification of Haematological malignancies-based ICD-O-3 histology codes

Cancer	HAEMACARE group number	ICD-O-3 histology codes	
		From CONCORD[1]	Mapped using SEER database
**All blood cancers**			
**All lymphoid malignancies**			
Composite Hodgkin and non-Hodgkin lymphoma		3	
Hodgkin lymphoma			
Hodgkin lymphoma, nodular lymphocyte predominance	4	9659	
Classical HL	5	9650, 9651, 9652, 9653, 9654, 9655, 9661, 9662, 9663, 9664, 9665, 9667	9656, 9657, 9658, 9666
Mature B-cell neoplasms			
Chronic lymphocytic leukaemia/Small lymphocytic lymphoma	6	9670, 9823	9620
Immunoproliferative diseases	7	9760, 9671, 9761, 9762	9611
Mantle cell/ centrocytic	8	9673	9622, 9674
Follicular lymphoma	9	9690, 9691, 9695, 9698	9614, 9615, 9676, 9692, 9693, 9697
Diffuse Large B-cell Lymphoma	10	9675, 9678, 9679, 9680, 9684, 9688, 9712, 9735, 9737, 9738	9632, 9681, 9682, 9635, 9683
Burkitt's	11	9687, 9826	
Marginal zone lymphoma	12	9689, 9699, 9764	9711, 9715, 9722
Mature B cell leukaemia	17	9833	
Mature B cell leukaemia, hairy cell	18	9940	
Plasma cells neoplasms (myeloma)	16	9731, 9734, 9732, 9733	9730, 9830
Mature T-cell and NK-cell neoplasms			
T lymphoma cutaneous	13	9700, 9701, 9709, 9718, 9708, 9726	
Other T cell lymphomas	14	9702, 9705, 9714, 9716, 9717, 9719, 9725, 9827, 9831, 9834, 9948,	9706, 9713

Lymphoblastic lymphoma/Acute (precursor cell) lymphatic leukaemia	15	9727, 9728, 9729, 9835, 9836, 9837, 9811, 9812, 9813, 9814, 9815, 9816, 9818	9602, 9685, 9821, 9819
Unknown lymphoid neoplasms			
Lymphoma, NOS	1	9590	
NH lymphoma, NOS	2	9591, 9597	9595, 9630, 9640, 9672, 9686, 9694, 9868, 9968
Lymphatic leukaemia, NOS	19	9820, 9832	9825, 9824, 9850
**All myeloid malignancies**			
Acute myeloid leukaemia	22	9840, 9861, 9865, 9866, 9867, 9869, 9870, 9871, 9872, 9873, 9874, 9891, 9895, 9896, 9897, 9910, 9911, 9920, 9930, 9931, 9984, 9987	9841, 9864, 9877, 9879, 9912
Myeloproliferative neoplasms	23	9740, 9741, 9742, 9863, 9875, 9950, 9961, 9962, 9963, 9964, 9960	
Myelodysplastic syndrome	24	9980, 9982, 9983, 9985, 9986, 9989, 9992, 9991	9981, 9993
Myelodysplastic/Myeloproliferative neoplasms	25	9945, 9876, 9946, 9975	9868, 9968
Unknown myeloid neoplasms			
Leukaemia, NOS	20	9800, 9801, 9805, 9806, 9807, 9808, 9809	9803, 9804
Myeloid leukaemia	21	9860, 9898	9862, 9880, 9890, 9893
**Other**			9724, 9750, 9751, 9752, 9753, 9754, 9755, 9756, 9757, 9758, 9759, 9765, 9766, 9767, 9768, 9769, 9965, 9966, 9967, 9970, 9971

[1]From Webtable 1 of the Supplement of Allemani, C., et al., Global surveillance of trends in cancer survival 2000–14 (CONCORD-3): analysis of individual records for 37 513 025 patients diagnosed with one of 18 cancers from 322 population-based registries in 71 countries. The Lancet, 2018. 391(10125): p. 1023-1075.

S3 Table: Summary of life table components, features, and sources

Nation	Life table component	Source	Latest year available	Adjusted for deprivation
England	Remaining life expectancy	Office of National Statistics(18)	2017	Yes
	Conditional survival probability	Office of National Statistics(18)	2017	Yes
Northern Ireland	Remaining life expectancy	Office of National Statistics(19)	2022	No
	Conditional survival probability	Office of National Statistics(19)	2022	No
Scotland	Remaining life expectancy	Human mortality database(20)	2021	No
	Conditional survival probability	CONCORD study(21)	2017	Yes
Wales	Remaining life expectancy	Office of National Statistics(18)	2017	No
	Conditional survival probability	Office of National Statistics(18)	2017	No

S4 Table: Statistical criteria for survival estimates by NCAS

In order to produce estimates that were statistically stable, all age groups had to pass the statistical tests below. These criteria were taken directly from the methodology published by the National Cancer Registration and Analysis Service (NCRAS) in England.

- a minimum of 10 patients should be alive at the beginning of the survival period being estimated (for example, first year of follow-up for a 1-year estimate, fifth year of follow-up for a 5-year estimate and tenth year of follow up for a 10-year estimate)
- at least two deaths registered in the years before or after the duration(s) being estimated
- the level of the survival estimates should not increase with duration (for example, the survival estimated at five years following diagnosis should be lower than the survival estimated at one year following diagnosis) the standard error of the survival estimates should be lower than 20%.

If any of these criteria were not met, the age-groupings were reduced to 4 age-groups with a single pair of adjacent age-groups being combined and the same tests were performed. If the 4-age group method did not meet the criteria, estimates were omitted altogether. All analyses run on Stata software using the strs function.(25)

On completion of data preparation and analysis, consistency checks were applied to ensure the results were valid and ready for publication. These include checking outputs against previous years data from prior publications. Most national reporting uses blood cancers categorised according to ICD-10 rather than ICD-O-3 codes, where the latter have been used in this report. Therefore, comparisons were restricted to comparable disease groups and/or used adapted survival estimates. In England, we compared myeloid leukaemia, myeloma, follicular NHL, Hodgkin lymphoma, non-Hodgkin lymphoma and leukaemia using estimates published for 2016 to 2020.(26) In Scotland and N. Ireland, we compared against published statistics for Hodgkin lymphoma and myeloma published by Public Health Scotland and the Northern Ireland Cancer Registry.(27, 28)

S5 Table: 1-year, 5-year and 10-year age-standardised survival rates and 95% CI in three nations for Figure 1

HAEMACARE group number	Blood Cancer subtype	n	1-year survival (%)	Lower 95% CI	Upper 95% CI	5-year survival (%)	Lower 95% CI	Upper 95% CI	10-year survival (%)	Lower 95% CI	Upper 95% CI	country
0	All blood cancer	342,169	80.1%	80.0%	80.3%	62.3%	62.1%	62.5%	51.3%	50.8%	51.7%	England
1	Lymphoma, NOS	5,013	58.8%	57.3%	60.3%	49.8%	48.1%	51.4%	43.8%	41.5%	46.2%	England
2	NH lymphoma, NOS	12,340	74.6%	73.8%	75.4%	62.9%	61.8%	63.9%	55.6%	53.7%	57.4%	England
3	Composite Hodgkin and non-HL	215	80.1%	73.4%	85.3%	62.8%	53.6%	70.6%	64.0%	48.9%	75.8%	England
4	HL, nodular lymphocyte predominance	1,756	98.8%	97.9%	99.3%	95.4%	93.6%	96.7%	94.6%	87.9%	97.6%	England
5	Classical HL	16,255	91.2%	90.8%	91.6%	84.4%	83.8%	84.9%	80.0%	79.1%	80.8%	England
6	Chronic lymphocytic leukaemia	40,900	94.3%	94.0%	94.5%	82.1%	81.5%	82.6%	67.5%	66.3%	68.7%	England
7	Immunoproliferative diseases	7,419	94.0%	93.3%	94.5%	82.5%	81.2%	83.7%	67.7%	64.8%	70.4%	England
8	Mantle cell/ centrocytic	5,558	82.6%	81.5%	83.6%	57.6%	56.1%	59.2%	41.4%	38.9%	44.0%	England
9	Follicular lymphoma	23,322	94.9%	94.5%	95.2%	84.3%	83.6%	85.0%	76.8%	74.8%	78.6%	England
10	Diffuse Large B-cell Lymphoma	47,963	73.6%	73.2%	74.0%	61.0%	60.5%	61.5%	55.5%	54.6%	56.4%	England
11	Burkitt's	1,647	50.0%	47.1%	52.7%	44.1%	41.0%	47.1%	41.7%	37.0%	46.4%	England
12	Marginal zone lymphoma	12,412	95.7%	95.2%	96.1%	85.9%	84.9%	86.8%	74.4%	71.5%	77.1%	England
13	T lymphoma cutaneous	3,783	91.6%	90.5%	92.6%	77.8%	75.7%	79.8%	76.9%	70.6%	82.0%	England
14	Other T cell lymphomas	7,197	61.8%	60.6%	62.9%	40.5%	39.2%	41.8%	34.7%	32.2%	37.2%	England
15	Lymphoblastic lymphoma/Acute (precursor cell) lymphatic leukaemia	3,761	73.0%	71.7%	74.3%	51.7%	50.1%	53.2%	48.2%	46.4%	49.9%	England
16	Plasma cells neoplasms	52,272	83.1%	82.7%	83.4%	56.1%	55.6%	56.6%	37.0%	35.3%	38.7%	England
17	Mature B cell leukaemia	72	77.7%	64.6%	86.4%	50.7%	36.0%	63.7%				England
18	Mature B cell leukaemia, hairy cell	2,137	94.4%	93.0%	95.6%	89.4%	86.6%	91.6%	86.2%	80.1%	90.5%	England
19	Lymphatic leukaemia,NOS	644	82.9%	79.7%	85.6%	65.2%	60.6%	69.5%	57.7%	47.9%	66.4%	England
20	Leukaemia,NOS	1,305	51.7%	48.7%	54.7%	35.6%	32.4%	38.8%	30.3%	26.7%	34.0%	England
21	Myeloid leukaemia, NOS	866	66.8%	62.9%	70.3%	43.0%	38.7%	47.2%	29.8%	24.5%	35.2%	England
22	Acute myeloid leukaemia	27,882	43.5%	42.9%	44.1%	22.5%	22.0%	23.0%	19.4%	18.8%	20.0%	England

**23**	**Myeloproliferative neoplasms**	**30,312**	**92.5%**	**92.2%**	**92.8%**	**77.9%**	**77.2%**	**78.5%**	**63.0%**	**61.4%**	**64.5%**
**24**	**Myelodysplastic syndrome**	**28,376**	**75.5%**	**74.8%**	**76.1%**	**40.7%**	**39.8%**	**41.5%**	**26.7%**	**25.7%**	**27.8%**
**25**	**Myelodysplastic/Myeloproliferative neoplasms**	**6,830**	**77.2%**	**76.0%**	**78.3%**	**44.1%**	**42.5%**	**45.6%**	**29.8%**	**27.7%**	**31.9%**
**26**	**Others**	**1,932**	**80.2%**	**78.2%**	**82.1%**	**65.2%**	**62.3%**	**68.0%**	**54.5%**	**49.6%**	**59.1%**
**1**	**All lymphoid malignancies**	**244,451**	**83.5%**	**83.3%**	**83.6%**	**67.7%**	**67.5%**	**67.9%**	**56.5%**	**55.9%**	**57.1%**
**2**	**All Myeloid malignancies**	**95,571**	**71.6%**	**71.3%**	**71.9%**	**48.1%**	**47.7%**	**48.5%**	**37.3%**	**36.8%**	**37.9%**
**1.1**	**Hodgkin lymphoma (all)**	**18,011**	**91.9%**	**91.5%**	**92.2%**	**85.4%**	**84.9%**	**85.8%**	**81.2%**	**80.4%**	**82.0%**
**1.2**	**Mature B-cell neoplasms**	**193,702**	**85.6%**	**85.4%**	**85.7%**	**69.5%**	**69.2%**	**69.7%**	**57.3%**	**56.6%**	**58.0%**
**1.3**	**Mature T-cell and NK-cell neoplasms**	**10,980**	**71.8%**	**70.9%**	**72.6%**	**53.1%**	**51.9%**	**54.3%**	**49.0%**	**46.5%**	**51.6%**
**1.4**	**Unknown lymphoid neoplasms**	**17,997**	**70.6%**	**69.9%**	**71.2%**	**59.4%**	**58.5%**	**60.2%**	**52.5%**	**50.9%**	**54.0%**
**2.5**	**Unknown myeloid neoplasms**	**2,171**	**57.9%**	**55.5%**	**60.2%**	**38.4%**	**35.8%**	**41.0%**	**29.9%**	**26.9%**	**33.0%**
**0**	**All blood cancer**	**38,127**	**82.3%**	**81.9%**	**82.7%**	**65.0%**	**64.4%**	**65.6%**	**51.8%**	**50.8%**	**52.8%**
**1**	**Lymphoma, NOS**	**473**	**37.5%**	**31.7%**	**43.3%**	**25.8%**	**20.2%**	**31.8%**	**23.5%**	**17.5%**	**30.0%**
**2**	**NH lymphoma, NOS**	**751**	**73.6%**	**70.3%**	**76.7%**	**60.4%**	**56.2%**	**64.3%**	**51.9%**	**46.3%**	**57.3%**
**3**	**Composite Hodgkin and non-HL**										
**4**	**HL, nodular lymphocyte predominance**	**164**				**97.0%**	**87.2%**	**99.0%**			
**5**	**Classical HL**	**1,658**	**90.8%**	**89.5%**	**91.8%**	**82.7%**	**81.0%**	**84.3%**	**80.2%**	**77.7%**	**82.5%**
**6**	**Chronic lymphocytic leukaemia**	**3,641**	**95.4%**	**94.5%**	**96.1%**	**84.9%**	**83.0%**	**86.6%**	**67.2%**	**63.5%**	**70.6%**
**7**	**Immunoproliferative diseases**	**702**	**94.3%**	**91.9%**	**96.0%**	**78.2%**	**73.6%**	**82.1%**	**71.0%**	**62.4%**	**77.9%**
**8**	**Mantle cell/ centrocytic**	**545**	**84.2%**	**80.7%**	**87.1%**	**58.9%**	**54.1%**	**63.5%**	**45.8%**	**38.5%**	**52.7%**
**9**	**Follicular lymphoma**	**2,485**	**95.1%**	**93.8%**	**96.1%**	**85.5%**	**83.1%**	**87.6%**	**73.5%**	**68.8%**	**77.5%**
**10**	**Diffuse Large B-cell Lymphoma**	**4,869**	**75.1%**	**73.9%**	**76.3%**	**63.6%**	**62.0%**	**65.3%**	**53.7%**	**51.0%**	**56.3%**
**11**	**Burkitt's**	**208**	**55.8%**	**48.4%**	**62.7%**	**52.6%**	**44.5%**	**60.0%**			
**12**	**Marginal zone lymphoma**	**1,102**	**96.6%**	**94.9%**	**97.7%**	**86.8%**	**83.5%**	**89.5%**	**73.3%**	**66.5%**	**79.0%**
**13**	**T lymphoma cutaneous**	**372**	**89.7%**	**85.1%**	**92.9%**	**72.8%**	**65.0%**	**79.1%**	**60.5%**	**50.7%**	**69.0%**
**14**	**Other T cell lymphomas**	**817**	**57.6%**	**54.1%**	**60.9%**	**39.9%**	**36.1%**	**43.6%**	**30.3%**	**24.2%**	**36.5%**
**15**	**Lymphoblastic lymphoma/Acute (precursor cell) lymphatic leukaemia**	**359**	**74.7%**	**70.4%**	**78.4%**	**50.3%**	**45.1%**	**55.3%**	**45.1%**	**39.3%**	**50.7%**

16	Plasma cells neoplasms	4,981	80.9%	79.8%	82.0%	54.0%	52.3%	55.7%	34.2%	31.9%	36.6%	Scotland
17	Mature B cell leukaemia											Scotland
18	Mature B cell leukaemia, hairy cell											Scotland
19	Lymphatic leukaemia,NOS											Scotland
20	Leukaemia,NOS											Scotland
21	Myeloid leukaemia, NOS											Scotland
22	Acute myeloid leukaemia	2,450	42.3%	40.3%	44.2%	21.7%	20.0%	23.4%	18.3%	16.2%	19.9%	Scotland
23	Myeloproliferative neoplasms	4,779	95.2%	94.4%	95.9%	82.5%	80.9%	84.0%	68.6%	64.6%	72.2%	Scotland
24	Myelodysplastic syndrome	3,661	78.5%	76.7%	80.2%	44.8%	42.3%	47.3%	29.6%	24.2%	35.1%	Scotland
25	Myelodysplastic/Myeloproliferative neoplasms	860	81.0%	77.5%	84.0%	47.4%	42.8%	51.9%	63.9%	60.1 %	67.4%	Scotland
26	Others	3,008	93.3%	92.1%	94.3%	80.1%	78.0%	82.0%	63.9%	60.1%	67.4%	Scotland
1	All lymphoid malignancies	23,292	82.8%	82.2%	83.2%	67.3%	66.6%	68.0%	54.4%	53.1%	55.6%	Scotland
2	All Myeloid malignancies	11,827	78.0%	77.2%	78.8%	56.4%	55.3%	57.4%	43.4%	41.8%	45.1%	Scotland
1.1	Hodgkin lymphoma (all)	1,822	91.4%	90.3%	92.4%	84.0%	82.3%	85.5%	81.5%	79.1%	83.7%	Scotland
1.2	Mature B-cell neoplasms	18,669	85.4%	84.9%	85.9%	69.9%	69.1%	70.7%	55.8%	54.3%	57.2%	Scotland
1.3	Mature T-cell and NK-cell neoplasms	1,189	67.2%	64.4%	69.9%	50.1%	46.7%	53.4%	40.4%	35.1%	45.6%	Scotland
1.4	Unknown lymphoid neoplasms	1,238	61.4%	58.5%	64.2%	49.6%	46.2%	52.9%	44.0%	39.8%	48.2%	Scotland
2.5	Unknown myeloid neoplasms	77	42.7%	30.8%	54.1%							Scotland
0	All blood cancer	10,440	80.9%	80.1%	81.7%	63.6%	62.4%	64.7%	52.5%	50.4%	54.7%	Northern Ireland
1	Lymphoma, NOS	247	52.7%	45.2%	59.6%	45.8%	38.0%	53.3%	40.4%	30.9%	49.7%	Northern Ireland
2	NH lymphoma, NOS	217	81.8%	75.8%	86.4%	73.4%	64.8%	80.2%				Northern Ireland
3	Composite Hodgkin and non-HL	6										Northern Ireland
4	HL, nodular lymphocyte predominance	84	95.4%	88.9%	98.2%	92.6%	84.5%	96.6%				Northern Ireland
5	Classical HL	544	91.5%	89.3%	93.2%	84.9%	81.6%	87.7%	84.9%	74.0%	91.4%	Northern Ireland
6	Chronic lymphocytic leukaemia	1,226	96.2%	94.5%	97.4%	87.5%	83.9%	90.4%	72.0%	64.4%	78.2%	Northern Ireland

7	Immunoproliferative diseases	162	97.7%	91.4%	99.4%							Northern Ireland
8	Mantle cell/ centrocytic	143	88.6%	82.4%	92.7%	54.2%	44.1%	63.3%				Northern Ireland
9	Follicular lymphoma	843	93.6%	91.2%	95.3%	84.4%	80.0%	87.9%	74.4%	63.5%	82.4%	Northern Ireland
10	Diffuse Large B-cell Lymphoma	1,394	73.9%	71.5%	76.1%	64.3%	61.1%	67.3%	58.0%	50.8%	64.6%	Northern Ireland
11	Burkitt's	33										Northern Ireland
12	Marginal zone lymphoma	260	97.3%	92.9%	99.0%	92.2%	82.7%	96.6%	117.6%			Northern Ireland
13	T lymphoma cutaneous	71	89.3%	77.2%	95.2%	77.3%	58.9%	88.3%				Northern Ireland
14	Other T cell lymphomas	249	52.1%	45.6%	58.1%	35.8%	29.0%	42.6%	35.9%	25.7%	46.3%	Northern Ireland
15	Lymphoblastic lymphoma/Acute (precursor cell) lymphatic leukaemia	252	75.1%	69.6%	79.7%							Northern Ireland
16	Plasma cells neoplasms	1,534	87.0%	85.3%	88.6%	57.8%	54.6%	60.9%	35.2%	30.6%	39.9%	Northern Ireland
17	Mature B cell leukaemia		78.9%	35.7%	94.7%							Northern Ireland
18	Mature B cell leukaemia, hairy cell	56	102.4%			91.7%	54.8%	98.8%	95.1%	0.0%	100.0 %	Northern Ireland
19	Lymphatic leukaemia,NOS	6	85.5%	41.7%	97.2%	70.9%	27.1%	91.4%	95.1%	0.0%	100.0 %	Northern Ireland
20	Leukaemia,NOS	91	62.2%	48.4%	73.4%	48.2%	33.8%	61.2%				Northern Ireland
21	Myeloid leukaemia, NOS	30	48.0%	25.0%	67.9%							Northern Ireland
22	Acute myeloid leukaemia	775	44.1%	40.7%	47.4%	23.0%	19.9%	26.2%				Northern Ireland
23	Myeloproliferative neoplasms	985	91.2%	89.0%	93.0%	74.5%	70.5%	78.0%	56.8%	49.0%	63.9%	Northern Ireland
24	Myelodysplastic syndrome	980	75.2%	71.7%	78.4%	40.0%	35.4%	44.5%	23.9%	18.9%	29.2%	Northern Ireland
25	Myelodysplastic/Myeloproliferative neoplasms	108										Northern Ireland
26	Others	140	87.6%	80.4%	92.2%	70.0%	57.9%	79.2%				Northern Ireland
1	All lymphoid malignancies	7,331	84.2%	83.3%	85.1%	70.0%	68.5%	71.3%	59.7%	56.8%	62.5%	Northern Ireland

2	All Myeloid malignancies	2,969	72.7%	70.9%	74.3%	47.8%	45.6%	50.0%	33.8%	30.5%	37.0%	Northern Ireland
1.1	Hodgkin lymphoma (all)	628	91.9%	89.9%	93.5%	85.8%	82.8%	88.4%	84.3%	75.9%	90.0%	Northern Ireland
1.2	Mature B-cell neoplasms	5,655	87.4%	86.5%	88.3%	72.5%	70.9%	74.0%	60.6%	57.3%	63.8%	Northern Ireland
1.3	Mature T-cell and NK-cell neoplasms	320	59.6%	53.9%	64.8%	43.9%	37.4%	50.2%	44.8%	34.9%	54.1%	Northern Ireland
1.4	Unknown lymphoid neoplasms	470	67.7%	62.9%	72.0%	59.9%	54.3%	65.0%	54.8%	46.4%	62.4%	Northern Ireland
2.5	Unknown myeloid neoplasms	121	59.6%	46.2%	70.7%	43.4%	30.5%	55.6%				Northern Ireland
All	All blood cancer	22,550	76.80	76.20	77.40	60.20	59.40	61.20				Wales
1	Lymphoma, NOS	340	46.00 %	40.20 %	51.50 %							Wales
2	NH lymphoma, NOS	730	68.10 %	64.40 %	71.40 %	55.00%	50.50 %	59.20 %				Wales
3	Composite Hodgkin and non-HL	<20										Wales
4	HL, nodular lymphocyte predominance	100	99.60%	45.90 %	100.00 %	93.40 %	81.80 %	97.70 %				Wales
5	Classical HL	880	89.20	87.50 %	90.80 %	81.30 %	79.00 %	83.40 %				Wales
6	Chronic lymphocytic leukaemia	2,610	92.10 %	90.90 %	93.10 %	82.40 %	80.00 %	84.50 %				Wales
7	Immunoproliferative diseases	370	91.60 %	87.80 %	94.20 %	75.00 %	69.00 %	80.00 %	56.90 %	46.10 %	66.40 %	Wales
8	Mantle cell/ centrocytic	350	81.30 %	77.20 %	84.70 %	55.50 %	49.10 %	61.50 %				Wales
9	Follicular lymphoma	1,240	93.60 %	91.70 %	95.10 %	80.40 %	76.80 %	83.50 %	76.20 %	68.20 %	82.50 %	Wales
10	Diffuse Large B-cell Lymphoma	2,840	71.60 %	69.80 %	73.20 %	60.00 %	57.80 %	62.10 %				Wales
11	Burkitt's	80										Wales
12	Marginal zone lymphoma	600	95.10 %	92.60 %	96.70 %	82.50 %	76.80 %	86.90 %	70.80 %	58.50 %	80.00 %	Wales
13	T lymphoma cutaneous	170	91.10 %	84.80 %	94.90 %	75.30%	65.70 %	82.60 %				Wales
14	Other T cell lymphomas	340	58.00 %	52.40 %	63.20 %	39.60 %	33.70 %	45.40 %	34.74 %	27.60 %	41.90 %	Wales
15	Lymphoblastic lymphoma/Acute (precursor cell) lymphatic leukaemia	210	77.20 %	71.90 %	81.60 %	58.10 %	51.80 %	63.80 %				Wales

16	Plasma cells neoplasms	2,920	79.20 %	77.70 %	80.60 %	54.90 %	52.70 %	56.90 %	39.70 %	36.00 %	43.40 %	Wales
17	Mature B cell leukaemia	<20										Wales
18	Mature B cell leukaemia, hairy cell	110	93.10 %	83.70 %	97.20 %	79.30 %	65.90 %	87.90 %				Wales
19	Lymphatic leukaemia,NOS	60	83.80 %	70.70 %	91.40 %							Wales
20	Leukaemia,NOS	100	54.50 %	44.30 %	63.60 %							Wales
21	Myeloid leukaemia, NOS	30										Wales
22	Acute myeloid leukaemia	1,920	42.70 %	40.50 %	44.90 %	23.20 %	20.20 %	24.30 %	20.50 %	18.20 %	22.90 %	Wales
23	Myeloproliferative neoplasms	3,390	88.10 %	86.80 %	89.20 %	73.40 %	71.20 %	75.40 %	65.60 %	60.70 %	70.00 %	Wales
24	Myelodysplastic syndrome	2,440	67.80 %	65.20 %	70.40 %	38.00 %	34.70 %	41.20 %				Wales
25	Myelodysplastic/Myeloproliferative neoplasms	360	65.20 %	58.30 %	71.30 %	24.50 %	16.90 %	32.90 %				Wales
26	Others	390	81.60 %	77.20 %	85.30 %	69.00 %	62.70 %	74.50 %	63.20 %	54.10 %	70.90 %	Wales
1	All lymphoid malignancies	13,920	80.40 %	79.70 %	81.10 %	65.40 %	64.40 %	66.40 %				Wales
2	All Myeloid malignancies	8,240	70.50 %	69.40 %	71.50 %	50.50 %	49.20 %	51.80 %				Wales
1.1	Hodgkin lymphoma (all)	980	90.30 %	88.70 %	91.70 %	82.70 %	80.40 %	84.70 %				Wales
1.2	Mature B-cell neoplasms	11,110	83.00 %	82.30 %	83.70 %	67.60 %	66.50 %	68.70 %				Wales
1.3	Mature T-cell and NK-cell neoplasms	720	67.00 %	63.00 %	70.60 %	48.10 %	43.60 %	52.40 %				Wales
1.4	Unknown lymphoid neoplasms	1,120	61.30 %	58.10 %	64.30 %	50.90 %	47.10 %	54.60 %				Wales
2.5	Unknown myeloid neoplasms	130	61.30 %	58.10 %	64.30 %	50.90 %	47.10 %	54.60 %				Wales

S6 Table: 5-year age-standardised survival rates and 95% CI by periods in three nations

	Blood Cancer subtype	2009-2014	2015-2019	Country

HAEMACARE group number		5-year-survival	Lower 95% CI	Upper 95% CI	5-year-survival	Lower 95% CI	Upper 95% CI	
All	All blood cancer	60.5%	60.3%	60.8%	64.3%	64.0%	64.5%	England
1	Lymphoma, NOS	53.0%	51.0%	55.0%	42.6%	39.5%	45.7%	England
2	NH lymphoma, NOS	60.6%	59.2%	62.0%	65.4%	63.7%	66.9%	England
3	Composite Hodgkin and non-HL	67.9%	52.2%	79.3%	60.3%	48.6%	70.1%	England
4	HL, nodular lymphocyte predominance	95.1%	91.9%	97.1%	95.9%	93.4%	97.5%	England
5	Classical HL	83.1%	82.4%	83.8%	85.8%	85.1%	86.5%	England
6	Chronic lymphocytic leukaemia	80.4%	79.7%	81.1%	83.9%	83.1%	84.7%	England
7	Immunoproliferative diseases	81.3%	79.5%	82.9%	83.7%	81.7%	85.4%	England
8	Mantle cell/ centrocytic	54.6%	52.5%	56.6%	61.3%	59.0%	63.5%	England
9	Follicular lymphoma	83.6%	82.6%	84.6%	85.0%	83.9%	86.1%	England
10	Diffuse Large B-cell Lymphoma	59.9%	59.2%	60.6%	62.4%	61.6%	63.1%	England
11	Burkitt's	41.9%	37.8%	45.9%	46.5%	41.7%	51.1%	England
12	Marginal zone lymphoma	84.5%	83.1%	85.8%	87.3%	85.8%	88.6%	England
13	T lymphoma cutaneous	78.9%	76.2%	81.4%	76.1%	72.6%	79.2%	England
14	Other T cell lymphomas	39.2%	37.4%	41.0%	41.9%	39.9%	43.8%	England
15	Lymphoblastic lymphoma/Acute (precursor cell) lymphatic leukaemia	50.4%	48.2%	52.6%	53.2%	50.9%	55.4%	England
16	Plasma cells neoplasms	52.8%	52.1%	53.5%	59.8%	59.0%	60.6%	England
17	Mature B cell leukaemia	52.0%	29.6%	70.3%				England
18	Mature B cell leukaemia, hairy cell	87.1%	83.5%	90.0%	92.3%	87.3%	95.4%	England
19	Lymphatic leukaemia,NOS	64.4%	58.9%	69.4%	66.8%	58.0%	74.2%	England
20	Leukaemia,NOS	39.5%	35.7%	43.4%	26.8%	21.5%	32.3%	England
21	Myeloid leukaemia, NOS	41.3%	35.9%	46.6%	45.4%	38.2%	52.4%	England
22	Acute myeloid leukaemia	21.0%	20.3%	21.7%	24.4%	23.5%	25.2%	England
23	Myeloproliferative neoplasms	75.2%	74.2%	76.1%	80.4%	79.4%	81.2%	England
24	Myelodysplastic syndrome	42.2%	41.1%	43.4%	38.6%	37.3%	39.9%	England
25	Myelodysplastic/Myeloproliferative neoplasms	41.7%	39.3%	44.2%	45.6%	43.6%	47.6%	England

26	Others	63.6%	59.5%	67.3%	67.5%	63.3%	71.3%	England
1	All lymphoid malignancies	65.9%	65.6%	66.2%	69.6%	69.3%	70.0%	England
2	All Myeloid malignancies	45.8%	45.3%	46.3%	50.4%	49.9%	51.0%	England
1.1	Hodgkin lymphoma (all)	83.9%	83.2%	84.6%	87.0%	86.3%	87.7%	England
1.2	Mature B-cell neoplasms	67.6%	67.2%	67.9%	71.5%	71.2%	71.9%	England
1.3	Mature T-cell and NK-cell neoplasms	53.6%	52.0%	55.1%	52.5%	50.8%	54.3%	England
1.4	Unknown lymphoid neoplasms	58.5%	57.3%	59.6%	60.6%	59.1%	61.9%	England
2.5	Unknown myeloid neoplasms	40.2%	37.0%	43.3%	34.5%	30.0%	38.9%	England
All	All blood cancer	64.7%	63.9%	65.4%	65.3%	64.4%	66.1%	Scotland
1	Lymphoma, NOS	26.8%	19.5%	34.7%	23.6%	16.5%	31.5%	Scotland
2	NH lymphoma, NOS	56.7%	51.2%	61.8%	63.8%	56.7%	70.1%	Scotland
3	Composite Hodgkin and non-HL							Scotland
4	HL, nodular lymphocyte predominance	96.8%	74.0%	99.6%	98.0%	79.0%	99.8%	Scotland
5	Classical HL	80.8%	78.3%	83.0%	85.3%	82.7%	87.5%	Scotland
6	Chronic lymphocytic leukaemia	85.7%	83.3%	87.8%	82.9%	79.7%	85.6%	Scotland
7	Immunoproliferative diseases	76.8%	70.6%	81.9%	80.1%	73.1%	85.5%	Scotland
8	Mantle cell/ centrocytic	55.3%	48.5%	61.5%	62.1%	54.9%	68.6%	Scotland
9	Follicular lymphoma	85.1%	81.6%	87.9%	86.0%	82.4%	89.0%	Scotland
10	Diffuse Large B-cell Lymphoma	61.8%	59.5%	64.0%	66.0%	63.6%	68.3%	Scotland
11	Burkitt's	52.1%	41.6%	61.6%	54.5%	41.2%	65.9%	Scotland
12	Marginal zone lymphoma	86.8%	82.0%	90.4%	86.3%	81.1%	90.2%	Scotland
13	T lymphoma cutaneous	76.2%	66.1%	83.7%	68.2%	54.5%	78.5%	Scotland
14	Other T cell lymphomas	39.0%	33.8%	44.1%	41.3%	36.0%	46.6%	Scotland
15	Lymphoblastic lymphoma/Acute (precursor cell) lymphatic leukaemia	49.7%	42.9%	56.2%	51.6%	43.4%	59.2%	Scotland
16	Plasma cells neoplasms	50.4%	48.1%	52.6%	58.1%	55.5%	60.6%	Scotland
17	Mature B cell leukaemia							Scotland
18	Mature B cell leukaemia, hairy cell							Scotland
19	Lymphatic leukaemia,NOS							Scotland

20	Leukaemia,NOS							Scotland
21	Myeloid leukaemia, NOS							Scotland
22	Acute myeloid leukaemia	20.5%	18.3%	22.7%	23.0%	20.5%	25.5%	Scotland
23	Myeloproliferative neoplasms	83.1%	80.9%	85.1%	81.8%	79.2%	84.0%	Scotland
24	Myelodysplastic syndrome	46.2%	42.8%	49.6%	42.9%	39.1%	46.7%	Scotland
25	Myelodysplastic/Myeloproliferative neoplasms	47.1%	40.9%	52.9%	47.2%	39.9%	54.1%	Scotland
26	Others	84.2%	81.3%	86.7%	75.2%	71.9%	78.2%	Scotland
1	All lymphoid malignancies	66.1%	65.1%	67.1%	68.6%	67.5%	69.7%	Scotland
2	All Myeloid malignancies	56.9%	55.5%	58.4%	55.6%	53.9%	57.2%	Scotland
1.1	Hodgkin lymphoma (all)	81.8%	79.5%	83.9%	86.7%	84.3%	88.7%	Scotland
1.2	Mature B-cell neoplasms	68.8%	67.7%	69.9%	71.1%	69.8%	72.3%	Scotland
1.3	Mature T-cell and NK-cell neoplasms	51.7%	47.0%	56.2%	48.4%	43.3%	53.3%	Scotland
1.4	Unknown lymphoid neoplasms	47.6%	43.2%	51.9%	51.2%	45.8%	56.4%	Scotland
2.5	Unknown myeloid neoplasms							Scotland
All	All blood cancer	61.3%	59.7%	62.8%	66.4%	64.6%	68.1%	Northern Ireland
1	Lymphoma, NOS	58.77%	49.04%	67.26%				Northern Ireland
2	NH lymphoma, NOS	64.51%	53.52%	73.53%	78.70%	64.92%	87.57%	Northern Ireland
3	Composite Hodgkin and non-HL							Northern Ireland
4	HL, nodular lymphocyte predominance	95.28%	72.62%	99.27%				Northern Ireland
5	Classical HL	84.28%	79.58%	87.98%	84.83%	79.37%	88.95%	Northern Ireland
6	Chronic lymphocytic leukaemia	86.72%	81.45%	90.58%	89.20%	83.68%	92.93%	Northern Ireland
7	Immunoproliferative diseases	86.45%	68.17%	94.62%				Northern Ireland
8	Mantle cell/ centrocytic	57.74%	42.45%	70.32%				Northern Ireland
9	Follicular lymphoma	83.88%	77.93%	88.35%	85.03%	77.55%	90.17%	Northern Ireland
10	Diffuse Large B-cell Lymphoma	62.13%	57.69%	66.25%	67.32%	62.70%	71.51%	Northern Ireland
11	Burkitt's							Northern Ireland
12	Marginal zone lymphoma	90.20%	73.12%	96.66%	92.85%	76.75%	97.94%	Northern Ireland
13	T lymphoma cutaneous	72.18%	49.14%	86.11%				Northern Ireland
14	Other T cell lymphomas	31.63%	23.62%	39.91%				Northern Ireland

15	Lymphoblastic lymphoma/Acute (precursor cell) lymphatic leukaemia							Northern Ireland
16	Plasma cells neoplasms	52.31%	47.56%	56.84%	62.98%	58.17%	67.40%	Northern Ireland
17	Mature B cell leukaemia							Northern Ireland
18	Mature B cell leukaemia, hairy cell	73.28%	41.13%	89.70%	109.24%			Northern Ireland
19	Lymphatic leukaemia,NOS	53.72%	17.91%	79.89%	124.74%			Northern Ireland
20	Leukaemia,NOS	45.49%	25.43%	63.57%	48.22%	33.06%	61.84%	Northern Ireland
21	Myeloid leukaemia, NOS							Northern Ireland
22	Acute myeloid leukaemia	22.49%	18.47%	26.75%				Northern Ireland
23	Myeloproliferative neoplasms	73.62%	68.42%	78.10%	74.63%	67.68%	80.30%	Northern Ireland
24	Myelodysplastic syndrome	39.24%	33.56%	44.87%	41.57%	34.14%	48.84%	Northern Ireland
25	Myelodysplastic/Myeloproliferative neoplasms							Northern Ireland
26	Others	68.27%	53.09%	79.45%	74.69%	54.43%	86.93%	Northern Ireland
1	All lymphoid malignancies	67.8%	65.9%	69.7%	72.4%	70.3%	74.5%	Northern Ireland
2	All Myeloid malignancies	0	0	0	0	0	1	Northern Ireland
1.1	Hodgkin lymphoma (all)	85.3%	80.9%	88.7%	85.63%	80.78%	89.35%	Northern Ireland
1.2	Mature B-cell neoplasms	70.4%	68.2%	72.4%	75.23%	72.85%	77.44%	Northern Ireland
1.3	Mature T-cell and NK-cell neoplasms	39.7%	31.7%	47.6%	48.15%	37.15%	58.31%	Northern Ireland
1.4	Unknown lymphoid neoplasms	60.4%	53.2%	66.9%	58.72%	49.93%	66.49%	Northern Ireland
2.5	Unknown myeloid neoplasms	37.4%	22.5%	52.3%	47.91%	33.21%	61.19%	Northern Ireland
All	All blood cancer	58.8%	57.8%	59.8%	61.8%	60.6%	63.1%	Wales
1	Lymphoma, NOS							Wales
2	NH lymphoma, NOS	51.5%	45.5%	57.2%	59.4%	52.8%	65.4%	Wales
3	Composite Hodgkin and non-HL							Wales
4	HL, nodular lymphocyte predominance							Wales
5	Classical HL	78.1%	74.5%	81.3%	84.8%	81.8%	87.4%	Wales
6	Chronic lymphocytic leukaemia	82.9%	79.9%	85.5%	81.3%	77.3%	84.7%	Wales
7	Immunoproliferative diseases	72.5%	65.2%	78.5%	79.4%	68.5%	86.9%	Wales

8	Mantle cell/ centrocytic	54%	45.5%	61.7%	58.2%	49.4%	65.9%	Wales
9	Follicular lymphoma	80.6%	75.7%	84.7%	80.5%	74.7%	85.1%	Wales
10	Diffuse Large B-cell Lymphoma	59.2%	56.2%	62%	61.1%	57.9%	64.2%	Wales
11	Burkitt's							Wales
12	Marginal zone lymphoma	81.6%	73.8%	87.3%	83.7%	73.8%	90.1%	Wales
13	T lymphoma cutaneous	70.4%	58.1%	79.7%				Wales
14	Other T cell lymphomas	35.5%	27.2%	43.8%				Wales
15	Lymphoblastic lymphoma/Acute (precursor cell) lymphatic leukaemia							Wales
16	Plasma cells neoplasms	51.6%	48.7%	54.4%	58.6%	55.3%	61.7%	Wales
17	Mature B cell leukaemia							Wales
18	Mature B cell leukaemia, hairy cell	86.9%	62.1%	96%	70.9%	52.8%	83.1%	Wales
19	Lymphatic leukaemia,NOS							Wales
20	Leukaemia,NOS							Wales
21	Myeloid leukaemia, NOS							Wales
22	Acute myeloid leukaemia	22.2%	19.7%	24.9%	24.8%	21.6%	28.2%	Wales
23	Myeloproliferative neoplasms	72.3%	69.6%	74.9%	74.9%	71.2%	78.2%	Wales
24	Myelodysplastic syndrome	36.5%	32.4%	40.5%	40.1%	34.8%	45.4%	Wales
25	Myelodysplastic/Myeloproliferative neoplasms							Wales
26	Others	67.9%	61.3%	73.6%				Wales
1	All lymphoid malignancies	64.1%	62.8%	65.4%	66.9%	65.3%	68.4%	Wales
2	All Myeloid malignancies	49.7%	48.1%	51.4%	51.7%	49.5%	53.8%	Wales
1.1	Hodgkin lymphoma (all)	78.6%	75.1%	81.6%	86.8%	84%	89.2%	Wales
1.2	Mature B-cell neoplasms	66.7%	65.2%	68.1%	68.8%	67%	70.4%	Wales
1.3	Mature T-cell and NK-cell neoplasms	45.4%	39.3%	51.3%				Wales
1.4	Unknown lymphoid neoplasms	49.3%	44.3%	54.1%	52.9%	47%	58.4%	Wales
2.5	Unknown myeloid neoplasms							

S7 Table: 5-year survival rates and 95% CI by age groups in three nations for Figure S1

HAEMACARE group number	Blood Cancer subtype	15-44 years old			45-54 years old			55-64 years old			65-74 years old			75-99 years old			Country
		5-year-survival	Lower 95% CI	Upper 95% CI	5-year-survival	Lower 95% CI	Upper 95% CI	5-year-survival	Lower 95% CI	Upper 95% CI	5-year-survival	Lower 95% CI	Upper 95% CI	5-year-survival	Lower 95% CI	Upper 95% CI	
All	All blood cancer	86.0%	85.5%	86.4%	79.9%	79.4%	80.4%	72.7%	72.3%	73.1%	61.8%	61.4%	62.1%	41.6%	41.2%	42.0%	England
1	Lymphoma, NOS	77.0%	71.6%	81.4%	66.0%	60.5%	71.0%	60.4%	56.2%	64.3%	53.3%	49.9%	56.6%	24.5%	22.1%	26.9%	England
2	NH lymphoma, NOS	81.9%	78.5%	84.8%	75.4%	72.2%	78.3%	74.6%	72.3%	76.8%	64.2%	62.2%	66.1%	42.5%	40.5%	44.4%	England
3	Composite Hodgkin and non-HL	77.0%	60.1%	87.5%	70.2%	45.5%	85.3%	67.6%	46.7%	81.8%	63.4%	44.4%	77.5%	51.7%	33.1%	67.5%	England
4	HL, nodular lymphocyte predominance	99.2%	97.7%	99.7%	95.6%	91.9%	97.6%	92.7%	87.7%	95.7%	90.2%	82.9%	94.5%	80.8%	64.1%	90.3%	England
5	Classical HL	95.7%	95.2%	96.2%	90.6%	89.1%	92.0%	79.4%	77.3%	81.3%	60.3%	57.8%	62.6%	39.0%	36.2%	41.7%	England
6	Chronic lymphocytic leukaemia	93.1%	90.6%	95.0%	93.9%	92.8%	94.9%	90.6%	89.7%	91.4%	84.9%	84.0%	85.7%	64.9%	63.5%	66.2%	England
7	Immunoproliferative diseases	95.4%	88.1%	98.3%	92.4%	88.7%	94.9%	89.1%	86.7%	91.1%	83.2%	81.0%	85.1%	69.4%	66.5%	72.2%	England
8	Mantle cell/ centrocytic	84.4%	75.7%	90.2%	79.5%	74.7%	83.4%	66.8%	63.5%	69.8%	55.7%	53.0%	58.3%	36.8%	33.9%	39.7%	England
9	Follicular lymphoma	95.8%	94.7%	96.8%	93.2%	92.2%	94.1%	89.8%	88.8%	90.8%	85.8%	84.7%	86.9%	71.9%	69.8%	73.9%	England
10	Diffuse Large B-cell Lymphoma	83.4%	82.1%	84.6%	74.2%	72.8%	75.6%	69.2%	68.1%	70.3%	60.1%	59.2%	61.1%	44.5%	43.5%	45.6%	England
11	Burkitt's	74.2%	70.3%	77.7%	65.0%	58.4%	70.7%	48.9%	42.1%	55.4%	43.5%	37.1%	49.6%	25.0%	19.2%	31.3%	England
12	Marginal zone lymphoma	96.4%	94.5%	97.7%	94.5%	92.7%	95.9%	90.2%	88.5%	91.6%	85.3%	83.7%	86.8%	77.1%	74.4%	79.5%	England
13	T lymphoma cutaneous	94.1%	91.7%	95.8%	90.1%	87.0%	92.4%	83.5%	80.1%	86.4%	77.9%	74.3%	81.0%	64.3%	58.4%	69.6%	England
14	Other T cell lymphomas	64.3%	60.9%	67.4%	55.9%	52.2%	59.3%	43.5%	40.7%	46.2%	39.4%	37.0%	41.8%	27.1%	24.4%	30.0%	England
15	Lymphoblastic lymphoma/Acute (precursor cell) lymphatic leukaemia	68.0%	65.7%	70.1%	45.0%	40.1%	49.8%	30.0%	26.0%	34.1%	24.1%	20.4%	28.1%	9.9%	6.9%	13.5%	England

16	Plasma cells neoplasms	79.4%	76.8%	81.7%	74.0%	72.5%	75.5%	67.2%	66.1%	68.2%	55.3%	54.4%	56.2%	35.0%	34.1%	35.9%	England
17	Mature B cell leukaemia	100.6%	100.6%	100.6%	51.4%	6.0%	85.5%	52.7%	11.4%	82.8%	65.3%	38.3%	82.8%	22.3%	6.9%	43.1%	England
18	Mature B cell leukaemia, hairy cell	98.0%	94.2%	99.3%	98.3%	95.0%	99.4%	95.3%	91.9%	97.3%	94.4%	89.7%	97.0%	73.9%	65.5%	80.6%	England
19	Lymphatic leukaemia,NOS	77.3%	55.8%	89.3%	75.7%	60.9%	85.6%	80.7%	69.9%	88.0%	66.2%	57.5%	73.5%	44.8%	35.8%	53.4%	England
20	Leukaemia,NOS	60.6%	51.8%	68.4%	60.3%	48.1%	70.4%	51.0%	42.4%	58.9%	29.7%	23.7%	35.9%	13.1%	9.7%	17.0%	England
21	Myeloid leukaemia, NOS	68.2%	49.5%	81.2%	70.0%	53.8%	81.4%	57.7%	45.7%	67.9%	32.4%	25.1%	39.9%	24.7%	18.7%	31.1%	England
22	Acute myeloid leukaemia	60.5%	58.6%	62.3%	50.3%	48.2%	52.4%	32.7%	31.2%	34.3%	13.3%	12.5%	14.2%	3.0%	2.6%	3.4%	England
23	Myeloproliferative neoplasms	96.4%	95.6%	97.0%	93.6%	92.6%	94.5%	87.1%	85.9%	88.2%	77.9%	76.7%	79.0%	59.6%	58.0%	61.2%	England
24	Myelodysplastic syndrome	72.3%	68.2%	76.0%	61.1%	57.5%	64.6%	47.2%	45.0%	49.4%	37.1%	35.8%	38.4%	22.9%	22.0%	23.8%	England
25	Myelodysplastic/Myeloproliferative neoplasms	91.7%	85.6%	95.3%	68.4%	61.5%	74.3%	52.7%	48.6%	56.6%	35.4%	32.9%	37.9%	24.3%	22.2%	26.4%	England
26	Others	80.0%	73.6%	85.0%	74.6%	66.9%	80.8%	74.0%	67.5%	79.4%	68.4%	63.1%	73.1%	47.6%	41.3%	53.6%	England
1	All lymphoid malignancies	88.1%	87.7%	88.5%	82.0%	81.5%	82.6%	76.6%	76.2%	77.1%	68.3%	67.9%	68.7%	49.1%	48.6%	49.6%	England
2	All Myeloid malignancies	79.3%	78.3%	80.3%	73.2%	72.1%	74.3%	59.0%	58.0%	59.9%	42.8%	42.1%	43.5%	26.8%	26.2%	27.4%	England
1.1	Hodgkin lymphoma (all)	96.0%	95.6%	96.4%	91.4%	90.0%	92.6%	81.3%	79.4%	83.0%	63.3%	61.0%	65.5%	41.6%	38.8%	44.3%	England
1.2	Mature B-cell neoplasms	86.8%	86.1%	87.5%	83.4%	82.8%	84.0%	78.6%	78.1%	79.1%	70.3%	69.8%	70.7%	51.5%	50.9%	52.0%	England
1.3	Mature T-cell and NK-cell neoplasms	76.7%	74.4%	78.8%	69.9%	67.3%	72.4%	57.2%	55.0%	59.5%	51.8%	49.7%	53.8%	38.5%	35.8%	41.1%	England
1.4	Unknown lymphoid neoplasms	80.2%	77.4%	82.7%	72.9%	70.2%	75.4%	71.2%	69.2%	73.0%	61.7%	60.0%	63.3%	37.2%	35.6%	38.7%	England
2.5	Unknown myeloid neoplasms	62.2%	54.3%	69.1%	63.8%	54.4%	71.9%	53.3%	46.4%	59.7%	30.8%	26.1%	35.6%	17.9%	14.7%	21.4%	England
All	All blood cancer	87.0%	85.7%	88.2%	81.7%	80.2%	83.1%	75.6%	74.4%	76.8%	64.3%	63.2%	65.4%	45.0%	43.7%	46.2%	Scotland
1	Lymphoma, NOS										26.5%	17.2%	36.6%	7.8%	4.1%	13.0%	Scotland
2	NH lymphoma, NOS				75.4%	62.3%	84.4%	76.0%	66.7%	83.1%	62.7%	54.0%	70.3%	33.5%	25.8%	41.3%	Scotland

3	Composite Hodgkin and non-HL																Scotland
4	HL, nodular lymphocyte predominance																Scotland
5	Classical HL	95.6%	93.7%	96.9%	86.2%	80.1%	90.5%	75.2%	67.7%	81.2%	56.0%	48.1%	63.1%	36.3%	28.3%	44.3%	Scotland
6	Chronic lymphocytic leukaemia	97.3%	83.1%	99.6%	94.6%	90.0%	97.1%	92.6%	89.4%	94.8%	85.0%	81.8%	87.7%	71.6%	66.7%	75.9%	Scotland
7	Immunoproliferative diseases				90.1%	70.5%	96.9%	83.8%	74.0%	90.2%	82.2%	74.1%	87.9%	62.9%	52.9%	71.4%	Scotland
8	Mantle cell/ centrocytic				96.9%	67.3%	99.8%	70.1%	59.4%	78.5%	49.6%	41.1%	57.6%	38.2%	28.8%	47.6%	Scotland
9	Follicular lymphoma	94.4%	89.4%	97.1%	94.9%	91.4%	97.0%	92.2%	88.8%	94.6%	87.3%	83.3%	90.4%	72.4%	65.3%	78.3%	Scotland
10	Diffuse Large B-cell Lymphoma	83.1%	78.7%	86.7%	72.8%	68.2%	76.9%	70.5%	67.1%	73.6%	67.0%	63.9%	69.8%	46.4%	42.9%	49.9%	Scotland
11	Burkitt's	71.7%	57.1%	82.0%				70.7%	53.2%	82.6%	61.9%	43.0%	76.2%	20.1%	7.5%	37.1%	Scotland
12	Marginal zone lymphoma	100.9%	100.9%	100.9%	97.3%	88.6%	99.4%	94.6%	88.3%	97.5%	86.4%	80.1%	90.9%	73.3%	64.1%	80.5%	Scotland
13	T lymphoma cutaneous	97.3%	85.4%	99.5%	95.6%	78.6%	99.2%	79.4%	67.1%	87.5%	73.0%	60.0%	82.4%	51.9%	31.0%	69.3%	Scotland
14	Other T cell lymphomas	63.1%	50.9%	73.0%	51.7%	41.0%	61.4%	51.5%	42.9%	59.4%	32.3%	25.7%	39.1%	27.8%	20.8%	35.3%	Scotland
15	Lymphoblastic lymphoma/Acute (precursor cell) lymphatic leukaemia	66.0%	58.2%	72.7%	46.1%	30.4%	60.5%	34.2%	18.9%	50.1%	17.2%	8.2%	29.0%	9.7%	2.9%	21.5%	Scotland
16	Plasma cells neoplasms	76.0%	65.6%	83.6%	71.7%	66.1%	76.5%	66.3%	62.6%	69.7%	53.6%	50.7%	56.5%	32.1%	29.3%	35.0%	Scotland
17	Mature B cell leukaemia																Scotland
18	Mature B cell leukaemia, hairy cell																Scotland
19	Lymphatic leukaemia,NOS																Scotland
20	Leukaemia,NOS																Scotland
21	Myeloid leukaemia, NOS																Scotland
22	Acute myeloid leukaemia	66.4%	60.2%	71.9%	47.1%	39.9%	53.9%	31.1%	26.4%	35.8%	12.4%	9.7%	15.3%	2.2%	1.2%	3.6%	Scotland

**23**	**Myeloproliferative neoplasms**	**97.1%**	**94.5%**	**98.5%**	**96.0%**	**93.0%**	**97.7%**	**92.2%**	**89.4%**	**94.3%**	**80.5%**	**77.5%**	**83.2%**	**67.7%**	**63.5%**	**71.6%**	**Scotland**
**24**	**Myelodysplastic syndrome**	**75.1%**	**59.8%**	**85.2%**	**62.3%**	**50.0%**	**72.4%**	**52.7%**	**46.5%**	**58.5%**	**40.3%**	**36.6%**	**44.1%**	**28.5%**	**25.6%**	**31.3%**	**Scotland**
**25**	**Myelodysplastic/Myeloproliferative neoplasms**							**50.3%**	**37.2%**	**62.0%**	**40.3%**	**33.3%**	**47.1%**	**26.8%**	**21.2%**	**32.7%**	**Scotland**
**26**	**Others**	**85.6%**	**75.4%**	**91.8%**	**89.8%**	**83.0%**	**93.9%**	**87.8%**	**83.3%**	**91.2%**	**83.0%**	**79.2%**	**86.2%**	**65.6%**	**61.1%**	**69.7%**	**Scotland**
**1**	**All lymphoid malignancies**	**87.9%**	**86.4%**	**89.3%**	**82.0%**	**80.2%**	**83.7%**	**77.6%**	**76.1%**	**78.9%**	**67.9%**	**66.5%**	**69.2%**	**47.5%**	**45.8%**	**49.2%**	**Scotland**
**2**	**All Myeloid malignancies**	**84.8%**	**81.9%**	**87.3%**	**79.0%**	**75.8%**	**81.9%**	**67.7%**	**65.2%**	**70.1%**	**51.8%**	**49.7%**	**53.8%**	**35.7%**	**33.8%**	**37.7%**	**Scotland**
**1.1**	**Hodgkin lymphoma (all)**	**96.0%**	**94.2%**	**97.2%**	**88.0%**	**82.5%**	**91.9%**	**78.5%**	**71.8%**	**83.8%**	**58.4%**	**50.8%**	**65.2%**	**39.0%**	**30.9%**	**46.9%**	**Scotland**
**1.2**	**Mature B-cell neoplasms**	**86.4%**	**83.8%**	**88.7%**	**84.1%**	**82.1%**	**85.9%**	**79.6%**	**78.1%**	**81.1%**	**71.0%**	**69.5%**	**72.4%**	**51.3%**	**49.4%**	**53.2%**	**Scotland**
**1.3**	**Mature T-cell and NK-cell neoplasms**	**78.4%**	**70.2%**	**84.5%**	**65.9%**	**57.2%**	**73.3%**	**60.9%**	**53.9%**	**67.2%**	**44.5%**	**38.3%**	**50.5%**	**33.8%**	**26.4%**	**41.2%**	**Scotland**
**1.4**	**Unknown lymphoid neoplasms**	**73.4%**	**57.5%**	**84.2%**	**70.2%**	**58.6%**	**79.1%**	**65.4%**	**57.1%**	**72.5%**	**51.1%**	**44.2%**	**57.5%**	**21.3%**	**16.9%**	**26.1%**	**Scotland**
**2.5**	**Unknown myeloid neoplasms**													**22.0%**	**7.9%**	**40.6%**	**Scotland**
**All**	**All blood cancer**	**87.4%**	**85.2%**	**89.2%**	**80.67%**	**77.77%**	**83.23%**	**75.09%**	**72.64%**	**77.35%**	**61.30%**	**59.06%**	**63.45%**	**43.95%**	**41.40%**	**46.46%**	**Northern Ireland**
**1**	**Lymphoma, NOS**	**83.9%**	**26.3%**	**97.7%**	**77.54%**	**52.16%**	**90.54%**	**47.60%**	**24.26%**	**67.76%**	**52.95%**	**37.41%**	**66.28%**	**14.98%**	**8.42%**	**23.30%**	**Northern Ireland**
**2**	**NH lymphoma, NOS**	**100.7%**	**100.7%**	**100.7%**	**83.53%**	**43.05%**	**96.23%**	**89.90%**	**65.73%**	**97.33%**	**70.64%**	**54.00%**	**82.20%**	**52.18%**	**33.99%**	**67.56%**	**Northern Ireland**
**3**	**Composite Hodgkin and non-HL**	**100.5%**	**100.5%**	**100.5%**	**101.84%**	**101.84%**	**101.84%**				**107.17%**	**107.17%**	**107.17%**				**Northern Ireland**
**4**	**HL, nodular lymphocyte predominance**	**100.5%**	**100.5%**	**100.5%**	**92.83%**	**43.84%**	**99.33%**	**88.48%**	**57.54%**	**97.32%**	**91.55%**	**12.70%**	**99.62%**	**50.25%**	**8.50%**	**82.52%**	**Northern Ireland**

**5**	**Classical HL**	**95.1%**	**91.3%**	**97.2%**	**90.54%**	**77.50%**	**96.20%**	**82.08%**	**69.34%**	**89.90%**	**57.92%**	**44.09%**	**69.47%**	**48.43%**	**30.60%**	**64.15%**	**Northern Ireland**
**6**	**Chronic lymphocytic leukaemia**	**92.1%**	**68.4%**	**98.2%**	**100.80%**	**100.80%**	**100.80%**	**91.90%**	**85.89%**	**95.42%**	**87.19%**	**81.29%**	**91.33%**	**77.81%**	**67.43%**	**85.24%**	**Northern Ireland**
**7**	**Immunoproliferative diseases**				**87.46%**	**27.82%**	**98.61%**	**91.47%**	**61.15%**	**98.40%**	**82.48%**	**60.35%**	**92.92%**	**93.22%**	**27.93%**	**99.61%**	**Northern Ireland**
**8**	**Mantle cell/ centrocytic**	**100.7%**	**100.7%**	**100.7%**	**64.72%**	**23.69%**	**87.68%**	**73.13%**	**47.27%**	**87.75%**	**45.77%**	**28.11%**	**61.80%**	**32.13%**	**14.85%**	**50.88%**	**Northern Ireland**
**9**	**Follicular lymphoma**	**97.3%**	**85.1%**	**99.5%**	**95.82%**	**88.50%**	**98.52%**	**92.68%**	**86.24%**	**96.17%**	**81.85%**	**74.05%**	**87.50%**	**72.42%**	**59.69%**	**81.72%**	**Northern Ireland**
**10**	**Diffuse Large B-cell Lymphoma**	**84.0%**	**74.4%**	**90.2%**	**72.44%**	**63.70%**	**79.41%**	**74.78%**	**68.07%**	**80.28%**	**59.13%**	**53.40%**	**64.41%**	**53.06%**	**46.00%**	**59.63%**	**Northern Ireland**
**11**	**Burkitt's**	**76.6%**	**48.8%**	**90.6%**	**51.56%**	**5.87%**	**85.66%**	**65.63%**	**22.47%**	**88.80%**							**Northern Ireland**
**12**	**Marginal zone lymphoma**	**91.4%**	**48.3%**	**98.9%**	**101.98%**	**101.98%**	**101.98%**	**85.40%**	**69.20%**	**93.46%**	**82.77%**	**67.56%**	**91.28%**	**103.19%**	**103.19%**	**103.19%**	**Northern Ireland**
**13**	**T lymphoma cutaneous**	**84.0%**	**47.6%**	**96.0%**	**70.53%**	**37.38%**	**88.35%**	**66.94%**	**29.28%**	**87.70%**	**85.00%**	**43.77%**	**96.85%**	**79.15%**	**21.00%**	**96.56%**	**Northern Ireland**
**14**	**Other T cell lymphomas**	**63.7%**	**43.9%**	**78.2%**	**64.13%**	**42.68%**	**79.31%**	**37.90%**	**22.54%**	**53.17%**	**25.71%**	**15.52%**	**37.15%**	**25.61%**	**12.98%**	**40.30%**	**Northern Ireland**
**15**	**Lymphoblastic lymphoma/Acute**	**86.7%**	**81.0%**	**90.8%**	**64.12%**	**22.79%**	**87.50%**	**42.95%**	**19.18%**	**64.88%**	**18.30%**	**1.50%**	**50.32%**				**Northern**

	(precursor cell) lymphatic leukaemia																Ireland
16	Plasma cells neoplasms	88.4%	66.1%	96.4%	71.05%	61.46%	78.67%	66.94%	59.61%	73.24%	58.82%	53.15%	64.05%	36.63%	30.69%	42.59%	Northern Ireland
17	Mature B cell leukaemia										117.34%	117.34%	117.34%				Northern Ireland
18	Mature B cell leukaemia, hairy cell	89.7%	40.6%	98.7%	93.55%	42.18%	99.49%	92.40%	11.55%	99.71%	93.91%	7.87%	99.84%	88.80%	0.07%	99.81%	Northern Ireland
19	Lymphatic leukaemia,NOS	100.4%	100.4%	100.4%										63.83%	0.90%	95.81%	Northern Ireland
20	Leukaemia,NOS	87.0%	56.1%	96.7%	51.86%	0.82%	91.41%	81.57%	32.59%	96.37%	41.83%	14.78%	67.21%	17.20%	6.30%	32.59%	Northern Ireland
21	Myeloid leukaemia, NOS	100.6%	100.6%	100.6%				52.93%	0.69%	92.20%				7.58%	0.52%	28.20%	Northern Ireland
22	Acute myeloid leukaemia	65.7%	56.3%	73.5%	45.93%	34.07%	57.00%	38.32%	28.41%	48.15%	12.33%	7.91%	17.78%	1.74%	0.50%	4.50%	Northern Ireland
23	Myeloproliferative neoplasms	95.5%	89.3%	98.2%	92.07%	84.03%	96.15%	84.08%	75.76%	89.73%	74.70%	66.84%	80.97%	54.28%	44.69%	62.90%	Northern Ireland
24	Myelodysplastic syndrome	67.0%	42.7%	82.8%	66.74%	44.32%	81.79%	45.04%	34.02%	55.43%	37.31%	29.55%	45.04%	20.95%	16.09%	26.26%	Northern Ireland
25	Myelodysplastic/Myeloproliferative neoplasms							52.56%	18.63%	78.18%	27.34%	10.83%	46.93%	12.61%	1.61%	35.39%	Northern Ireland

**26**	**Others**	**81.8%**	**61.1%**	**92.1%**	**86.51%**	**59.64%**	**96.02%**	**90.32%**	**48.61%**	**98.57%**	**63.17%**	**38.13%**	**80.34%**	**50.89%**	**24.97%**	**71.98%**	**Northern Ireland**
**1**	**All lymphoid malignancies**	**89.9%**	**87.4%**	**91.8%**	**83.2%**	**79.9%**	**86.0%**	**79.2%**	**76.4%**	**81.6%**	**67.6%**	**65.0%**	**70.0%**	**54.8%**	**51.4%**	**58.0%**	**Northern Ireland**
**2**	**All Myeloid malignancies**	**1**	**1**	**1**	**1**	**1**	**1**	**1**	**1**	**1**	**0**	**0**	**0**	**0**	**0**	**0**	**Northern Ireland**
**1.1**	**Hodgkin lymphoma (all)**	**95.67%**	**92.25%**	**97.60%**	**90.93%**	**79.56%**	**96.12%**	**83.14%**	**72.10%**	**90.10%**	**61.76%**	**48.64%**	**72.46%**	**48.36%**	**31.79%**	**63.10%**	**Northern Ireland**
**1.2**	**Mature B-cell neoplasms**	**88.83%**	**83.89%**	**92.32%**	**83.93%**	**80.19%**	**87.02%**	**81.40%**	**78.45%**	**83.98%**	**70.19%**	**67.38%**	**72.81%**	**59.16%**	**55.34%**	**62.77%**	**Northern Ireland**
**1.3**	**Mature T-cell and NK-cell neoplasms**	**69.37%**	**52.83%**	**81.09%**	**66.06%**	**48.68%**	**78.75%**	**44.63%**	**30.00%**	**58.24%**	**36.61%**	**25.88%**	**47.39%**	**35.25%**	**21.83%**	**48.96%**	**Northern Ireland**
**1.4**	**Unknown lymphoid neoplasms**	**92.89%**	**53.89%**	**99.12%**	**79.59%**	**60.09%**	**90.27%**	**72.96%**	**56.92%**	**83.83%**	**62.40%**	**51.28%**	**71.67%**	**30.86%**	**22.60%**	**39.49%**	**Northern Ireland**
**2.5**	**Unknown myeloid neoplasms**	**89.85%**	**63.65%**	**97.50%**	**51.86%**	**0.82%**	**91.41%**	**76.11%**	**35.92%**	**92.98%**	**31.99%**	**11.38%**	**55.00%**	**14.01%**	**5.57%**	**26.25%**	**Northern Ireland**
**All**	**All blood cancer**	**86.9**	**85.1**	**88.5**	**80.1**	**78.1**	**82**	**70.8**	**69.2**	**72.4**	**57.1**	**55.7**	**58.5**	**40**	**38.3**	**41.7**	**Wales**
**1**	**Lymphoma, NOS**							**54**	**37.5**	**68**	**29.3**	**18.6**	**40.9**				**Wales**
**2**	**NH lymphoma, NOS**				**72.8**	**55.9**	**84.1**	**64.6**	**53.8**	**73.5**	**57.2**	**49.3**	**64.3**	**31.7**	**24**	**39.7**	**Wales**
**3**	**Composite Hodgkin and non-HL**																**Wales**
**4**	**HL, nodular lymphocyte predominance**																**Wales**

5	Classical HL	95%	92.2%	96.8%				72.9%	63.5%	80.3%	50%	40.4%	58.9%	27.8%	17.6%	38.8%	Wales
6	Chronic lymphocytic leukaemia				93.7%	87.8%	96.8%	91.3%	87.5%	94.1%	83.2%	79.4%	86.4%	66.9%	60.8%	72.3%	Wales
7	Immunoproliferative diseases							71.1%	57.5%	81%	87%	65.2%	93.4%	53.3%	39.8%	65%	Wales
8	Mantle cell/ centrocytic							81%	66.8%	89.6%	50.8%	39.1%	61.4%	28.5%	19%	38.7%	Wales
9	Follicular lymphoma	92.8%	83.3%	97%	89.2%	82.6%	93.4%				79.2%	73.6%	83.8%	71.1%	60.6%	79.3%	Wales
10	Diffuse Large B-cell Lymphoma	87.4%	81.1%	91.7%	74.3%	68.1%	79.4%	66.7%	62%	71%	58.6%	54.8%	62.2%	43.4%	38.8%	48%	Wales
11	Burkitt's																Wales
12	Marginal zone lymphoma				94.3%	77.2%	98.7%	87.6%	77.3%	93.4%	85.7%	77%	91.3%	65.9%	49.6%	78%	Wales
13	T lymphoma cutaneous										72.1%	53.6%	84.2%				Wales
14	Other T cell lymphomas							42.3%	30.4%	53.7%	40.3%	30.1%	50.3%	30%	18.8%	42%	Wales
15	Lymphoblastic lymphoma/Acute (precursor cell) lymphatic leukaemia																Wales
16	Plasma cells neoplasms				75.8%	68.8%	81.5%	68.5%	63.7%	72.7%	50.4%	46.6%	54.1%	32%	28.3%	35.9%	Wales
17	Mature B cell leukaemia																Wales
18	Mature B cell leukaemia, hairy cell																Wales
19	Lymphatic leukaemia,NOS																Wales
20	Leukaemia,NOS																Wales
21	Myeloid leukaemia, NOS																Wales
22	Acute myeloid leukaemia	62.7%	54.6%	69.8%	48.4%	39.6%	56.6%	33.2%	27.6%	39%	14.7%	11.7%	18%	3.9%	2.38%	5.99%	Wales
23	Myeloproliferative neoplasms	94.1%	90.8%	96.3%	92.3%	88.6%	94.9%	80.5%	76.6%	83.8%	70.4%	66.5%	73.9%	58%	52.4%	63.1%	Wales
24	Myelodysplastic syndrome				52.6%	36.1%	66.8%	45.6%	37.8%	53.2%	31.8%	27.4%	36.2%	25.5%	22%	29.1%	Wales
25	Myelodysplastic/Myeloproliferative neoplasms										19.7%	12.1%	28.7%	19.3%	12.3%	27.4%	Wales

26	Others							73.8%	57.5%	84.7%				43.9%	31.7%	55.4%	Wales
1	All lymphoid malignancies	88.9%	86.7%	90.7%	81%	78.5%	83.3%	75.2%	73.2%	77%	64.3%	62.5%	66%	46.7%	44.4%	48.9%	Wales
2	All Myeloid malignancies	82.8%	79.3%	85.8%	77.8%	73.9%	81.1%	60.9%	57.7%	63.9%	42.5%	40.2%	44.9%	31.1%	28.7%	33.6%	Wales
1.1	Hodgkin lymphoma (all)	95.2%	92.5%	96.9%				76%	67.3%	82.7%	56.9%	47.7%	65%	29.9%	19.7%	40.8%	Wales
1.2	Mature B-cell neoplasms	88.4%	84.8%	91.2%	83.4%	80.7%	85.8%	77.5%	75.4%	79.5%	66.8%	64.9%	68.7%	49.1%	46.5%	51.6%	Wales
1.3	Mature T-cell and NK-cell neoplasms	75.9%	68%	82%	49.2%	36.4%	60.8%	55.1%	46.1%	63.2%				36.8%	27.4%	46.3%	Wales
1.4	Unknown lymphoid neoplasms				60.8%	47.7%	71.6%	61.4%	52.5%	69.2%	50.1%	43.5%	56.4%	32.6%	25.7%	39.7%	Wales
2.5	Unknown myeloid neoplasms																Wales

S8 Table: 5-year age-standardised survival rates and 95% CI by sex in three nations for Figure S2

HAEMACARE group number	Blood Cancer subtype	Male			Female			country
		5-year-survival	Lower 95% CI	Upper 95% CI	5-year-survival	Lower 95% CI	Upper 95% CI	
All	All blood cancer	60.5%	60.2%	60.8%	64.8%	64.5%	65.1%	England
1	Lymphoma, NOS	47.3%	45.1%	49.5%	53.0%	50.5%	55.5%	England
2	NH lymphoma, NOS	61.0%	59.5%	62.4%	65.3%	63.8%	66.8%	England
3	Composite Hodgkin and non-HL	63.9%	50.5%	74.5%	63.0%	50.2%	73.4%	England
4	HL, nodular lymphocyte predominance	93.8%	91.1%	95.7%	97.8%	94.7%	99.1%	England
5	Classical HL	83.4%	82.6%	84.1%	85.7%	84.9%	86.4%	England
6	Chronic lymphocytic leukaemia	80.6%	79.9%	81.3%	84.4%	83.6%	85.2%	England
7	Immunoproliferative diseases	81.0%	79.3%	82.6%	85.4%	83.5%	87.2%	England
8	Mantle cell/ centrocytic	56.1%	54.2%	57.9%	62.2%	59.4%	64.9%	England
9	Follicular lymphoma	83.2%	82.0%	84.3%	85.3%	84.3%	86.3%	England
10	Diffuse Large B-cell Lymphoma	59.9%	59.2%	60.6%	62.5%	61.8%	63.2%	England

11	Burkitt's	42.8%	39.2%	46.4%	46.3%	40.5%	51.8%	England
12	Marginal zone lymphoma	85.1%	83.6%	86.5%	86.7%	85.5%	87.9%	England
13	T lymphoma cutaneous	76.5%	73.8%	79.0%	79.8%	76.2%	83.0%	England
14	Other T cell lymphomas	38.6%	36.8%	40.4%	43.3%	41.3%	45.3%	England
15	Lymphoblastic lymphoma/Acute (precursor cell) lymphatic leukaemia	51.9%	49.9%	53.9%	51.7%	49.1%	54.3%	England
16	Plasma cells neoplasms	55.7%	55.0%	56.4%	56.7%	55.9%	57.4%	England
17	Mature B cell leukaemia	49.3%	29.1%	66.7%				England
18	Mature B cell leukaemia, hairy cell	91.6%	88.1%	94.2%	84.2%	79.2%	88.2%	England
19	Lymphatic leukaemia,NOS	64.1%	57.5%	70.0%	67.3%	60.5%	73.1%	England
20	Leukaemia,NOS	35.4%	31.2%	39.6%	36.3%	31.5%	41.2%	England
21	Myeloid leukaemia, NOS	40.9%	35.2%	46.5%	46.4%	39.9%	52.8%	England
22	Acute myeloid leukaemia	21.4%	20.7%	22.1%	23.9%	23.1%	24.8%	England
23	Myeloproliferative neoplasms	73.3%	72.3%	74.2%	82.5%	81.6%	83.3%	England
24	Myelodysplastic syndrome	38.6%	37.5%	39.7%	43.9%	42.6%	45.3%	England
25	Myelodysplastic/Myeloproliferative neoplasms	40.3%	38.2%	42.4%	50.3%	47.9%	52.8%	England
26	Others	64.0%	59.7%	67.9%	67.2%	63.3%	70.8%	England
1	All lymphoid malignancies	66.5%	66.2%	66.8%	69.3%	69.0%	69.6%	England
2	All Myeloid malignancies	44.5%	44.0%	45.0%	52.9%	52.3%	53.4%	England
1.1	Hodgkin lymphoma (all)	84.4%	83.7%	85.1%	86.6%	85.9%	87.3%	England
1.2	Mature B-cell neoplasms	68.4%	68.0%	68.7%	71.0%	70.6%	71.4%	England
1.3	Mature T-cell and NK-cell neoplasms	51.9%	50.4%	53.4%	54.9%	53.1%	56.6%	England
1.4	Unknown lymphoid neoplasms	57.4%	56.2%	58.6%	62.0%	60.7%	63.3%	England
2.5	Unknown myeloid neoplasms	37.6%	34.2%	41.0%	40.0%	36.1%	43.9%	England
All	All blood cancer	63.6%	62.8%	64.4%	66.7%	65.8%	67.5%	Scotland
1	Lymphoma, NOS	22.4%	15.6%	29.9%				Scotland
2	NH lymphoma, NOS	55.4%	49.2%	61.1%	66.5%	60.9%	71.6%	Scotland
3	Composite Hodgkin and non-HL							Scotland
4	HL, nodular lymphocyte predominance	97.0%	76.5%	99.7%	97.5%	68.2%	99.8%	Scotland

**5**	**Classical HL**	**82.3%**	**79.8%**	**84.5%**	**83.3%**	**80.8%**	**85.6%**	**Scotland**
**6**	**Chronic lymphocytic leukaemia**	**84.8%**	**82.2%**	**87.0%**	**85.1%**	**82.3%**	**87.5%**	**Scotland**
**7**	**Immunoproliferative diseases**	**77.8%**	**71.6%**	**82.7%**	**78.2%**	**70.5%**	**84.1%**	**Scotland**
**8**	**Mantle cell/ centrocytic**	**60.1%**	**54.4%**	**65.3%**				**Scotland**
**9**	**Follicular lymphoma**	**84.5%**	**80.6%**	**87.7%**	**86.2%**	**83.0%**	**88.9%**	**Scotland**
**10**	**Diffuse Large B-cell Lymphoma**	**64.5%**	**62.1%**	**66.8%**	**62.8%**	**60.4%**	**65.0%**	**Scotland**
**11**	**Burkitt's**	**53.0%**	**43.1%**	**62.0%**	**49.4%**	**34.6%**	**62.5%**	**Scotland**
**12**	**Marginal zone lymphoma**	**85.1%**	**79.3%**	**89.4%**	**88.1%**	**83.7%**	**91.3%**	**Scotland**
**13**	**T lymphoma cutaneous**	**75.9%**	**65.0%**	**83.9%**	**68.1%**	**56.5%**	**77.3%**	**Scotland**
**14**	**Other T cell lymphomas**	**40.0%**	**34.9%**	**45.1%**	**40.2%**	**34.5%**	**45.7%**	**Scotland**
**15**	**Lymphoblastic lymphoma/Acute (precursor cell) lymphatic leukaemia**	**49.5%**	**42.7%**	**55.9%**	**52.1%**	**43.8%**	**59.6%**	**Scotland**
**16**	**Plasma cells neoplasms**	**53.1%**	**50.8%**	**55.3%**	**55.4%**	**52.8%**	**57.9%**	**Scotland**
**17**	**Mature B cell leukaemia**							**Scotland**
**18**	**Mature B cell leukaemia, hairy cell**							**Scotland**
**19**	**Lymphatic leukaemia,NOS**							**Scotland**
**20**	**Leukaemia,NOS**							**Scotland**
**21**	**Myeloid leukaemia, NOS**							**Scotland**
**22**	**Acute myeloid leukaemia**	**21.1%**	**18.9%**	**23.4%**	**22.5%**	**20.0%**	**25.2%**	**Scotland**
**23**	**Myeloproliferative neoplasms**	**78.5%**	**75.9%**	**80.8%**	**86.4%**	**84.3%**	**88.3%**	**Scotland**
**24**	**Myelodysplastic syndrome**	**43.7%**	**40.4%**	**47.0%**	**46.5%**	**42.6%**	**50.3%**	**Scotland**
**25**	**Myelodysplastic/Myeloproliferative neoplasms**	**45.7%**	**39.8%**	**51.3%**	**48.6%**	**40.5%**	**56.1%**	**Scotland**
**26**	**Others**	**79.2%**	**76.1%**	**81.9%**	**80.9%**	**77.9%**	**83.6%**	**Scotland**
**1**	**All lymphoid malignancies**	**67.0%**	**66.0%**	**68.1%**	**67.8%**	**66.7%**	**68.8%**	**Scotland**
**2**	**All Myeloid malignancies**	**53.2%**	**51.7%**	**54.6%**	**60.4%**	**58.8%**	**62.0%**	**Scotland**
**1.1**	**Hodgkin lymphoma (all)**	**83.8%**	**81.5%**	**85.8%**	**84.3%**	**81.9%**	**86.5%**	**Scotland**
**1.2**	**Mature B-cell neoplasms**	**69.6%**	**68.4%**	**70.7%**	**70.4%**	**69.2%**	**71.5%**	**Scotland**
**1.3**	**Mature T-cell and NK-cell neoplasms**	**51.3%**	**46.5%**	**55.9%**	**49.2%**	**44.2%**	**53.9%**	**Scotland**
**1.4**	**Unknown lymphoid neoplasms**	**45.7%**	**40.8%**	**50.4%**	**54.9%**	**50.3%**	**59.2%**	**Scotland**

**2.5**	**Unknown myeloid neoplasms**							**Scotland**
**All**	**All blood cancer**	**61.71%**	**60.09%**	**63.29%**	**65.92%**	**64.19%**	**67.60%**	**Northern Ireland**
**1**	**Lymphoma, NOS**	**47.86%**	**36.98%**	**57.94%**	**44.77%**	**33.84%**	**55.11%**	**Northern Ireland**
**2**	**NH lymphoma, NOS**	**69.32%**	**58.57%**	**77.80%**	**78.61%**	**63.90%**	**87.87%**	**Northern Ireland**
**3**	**Composite Hodgkin and non-HL**							**Northern Ireland**
**4**	**HL, nodular lymphocyte predominance**	**92.55%**	**81.05%**	**97.19%**				**Northern Ireland**
**5**	**Classical HL**	**84.83%**	**80.13%**	**88.50%**	**84.99%**	**80.08%**	**88.77%**	**Northern Ireland**
**6**	**Chronic lymphocytic leukaemia**	**86.68%**	**81.41%**	**90.54%**	**88.14%**	**81.77%**	**92.39%**	**Northern Ireland**
**7**	**Immunoproliferative diseases**							**Northern Ireland**
**8**	**Mantle cell/ centrocytic**	**57.48%**	**44.55%**	**68.44%**				**Northern Ireland**
**9**	**Follicular lymphoma**	**86.20%**	**79.04%**	**91.04%**	**83.03%**	**77.11%**	**87.54%**	**Northern Ireland**
**10**	**Diffuse Large B-cell Lymphoma**	**65.08%**	**60.75%**	**69.05%**	**63.43%**	**58.57%**	**67.89%**	**Northern Ireland**
**11**	**Burkitt's**							**Northern Ireland**
**12**	**Marginal zone lymphoma**	**89.48%**	**71.79%**	**96.34%**	**95.38%**	**77.80%**	**99.11%**	**Northern Ireland**
**13**	**T lymphoma cutaneous**	**63.85%**	**42.26%**	**79.17%**	**99.66%**	**0.00%**	**100.00%**	**Northern Ireland**
**14**	**Other T cell lymphomas**	**33.61%**	**25.48%**	**41.93%**	**37.18%**	**26.66%**	**47.69%**	**Northern Ireland**
**15**	**Lymphoblastic lymphoma/Acute (precursor cell) lymphatic leukaemia**							**Northern Ireland**
**16**	**Plasma cells neoplasms**	**56.63%**	**52.49%**	**60.55%**	**58.97%**	**53.69%**	**63.86%**	**Northern Ireland**
**17**	**Mature B cell leukaemia**							**Northern Ireland**
**18**	**Mature B cell leukaemia, hairy cell**	**95.79%**	**10.51%**	**99.92%**				**Northern Ireland**
**19**	**Lymphatic leukaemia,NOS**	**74.12%**	**8.43%**	**96.44%**	**70.21%**	**15.27%**	**93.56%**	**Northern Ireland**
**20**	**Leukaemia,NOS**				**63.70%**	**48.29%**	**75.62%**	**Northern Ireland**
**21**	**Myeloid leukaemia, NOS**							**Northern Ireland**
**22**	**Acute myeloid leukaemia**	**21.86%**	**17.75%**	**26.27%**	**24.58%**	**20.00%**	**29.42%**	**Northern Ireland**
**23**	**Myeloproliferative neoplasms**	**67.73%**	**61.28%**	**73.34%**	**79.69%**	**74.52%**	**83.92%**	**Northern Ireland**
**24**	**Myelodysplastic syndrome**	**36.44%**	**30.63%**	**42.25%**	**44.79%**	**37.77%**	**51.55%**	**Northern Ireland**
**25**	**Myelodysplastic/Myeloproliferative neoplasms**							**Northern Ireland**
**26**	**Others**	**65.36%**	**49.10%**	**77.54%**	**70.95%**	**49.09%**	**84.75%**	**Northern Ireland**

1	All lymphoid malignancies	69.5%	67.5%	71.3%	70.6%	68.5%	72.6%	Northern Ireland
2	All Myeloid malignancies	0	0	0	1	1	1	Northern Ireland
1.1	Hodgkin lymphoma (all)	86.01%	81.85%	89.27%	85.40%	80.83%	88.96%	Northern Ireland
1.2	Mature B-cell neoplasms	72.16%	69.94%	74.24%	72.99%	70.62%	75.20%	Northern Ireland
1.3	Mature T-cell and NK-cell neoplasms	40.38%	32.22%	48.38%	48.43%	38.00%	58.08%	Northern Ireland
1.4	Unknown lymphoid neoplasms	59.26%	51.90%	65.88%	61.10%	52.61%	68.53%	Northern Ireland
2.5	Unknown myeloid neoplasms				62.60%	47.86%	74.25%	Northern Ireland
All	All blood cancer	57.9%	56.9%	58.9%	63.2%	62%	64.3%	Wales
1	Lymphoma, NOS	33.2%	23.7%	43%				Wales
2	NH lymphoma, NOS	53.9%	47.7%	59.6%	56.5%	49.8%	62.7%	Wales
3	Composite Hodgkin and non-HL							Wales
4	HL, nodular lymphocyte predominance							Wales
5	Classical HL	80.8%	77.4%	83.7%	81.1%	77.5%	84.1%	Wales
6	Chronic lymphocytic leukaemia	80.6%	77.4%	83.4%	85.7%	82.2%	88.6%	Wales
7	Immunoproliferative diseases	73.8%	65.8%	80.1%	76.7%	66.3%	84.2%	Wales
8	Mantle cell/ centrocytic	54.5%	48%	60.6%				Wales
9	Follicular lymphoma	76.7%	70.7%	81.7%	82.9%	78.3%	86.6%	Wales
10	Diffuse Large B-cell Lymphoma				62.4%	59.3%	65.4%	Wales
11	Burkitt's							Wales
12	Marginal zone lymphoma	82%	74.2%	87.6%	82.4%	73.6%	88.5%	Wales
13	T lymphoma cutaneous	75.9%	62.2%	85.2%	75.8%	60.2%	86%	Wales
14	Other T cell lymphomas	36.7%	29.5%	44%	44.6%	34.8%	53.9%	Wales
15	Lymphoblastic lymphoma/Acute (precursor cell) lymphatic leukaemia							Wales
16	Plasma cells neoplasms	54.2%	51.3%	57.1%	55.8%	52.7%	58.8%	Wales
17	Mature B cell leukaemia							Wales
18	Mature B cell leukaemia, hairy cell	80.3%	63.5%	89.9%				Wales
19	Lymphatic leukaemia,NOS							Wales

20	Leukaemia,NOS							Wales
21	Myeloid leukaemia, NOS							Wales
22	Acute myeloid leukaemia	22.6%	20%	25.4%	23.9%	20.8%	27.2%	Wales
23	Myeloproliferative neoplasms	69.8%	66.9%	72.4%	76.8%	73.5%	79.8%	Wales
24	Myelodysplastic syndrome	34.1%	29.9%	38.4%	43.4%	38.3%	48.3%	Wales
25	Myelodysplastic/Myeloproliferative neoplasms	21.5%	13.7%	30.4%				Wales
26	Others	66.5%	57.7%	73.8%	70.7%	60.8%	78.5%	Wales
1	All lymphoid malignancies	64.1%	62.7%	65.4%	67.2%	65.7%	68.6%	Wales
2	All Myeloid malignancies	47.3%	45.6%	48.9%	54.8%	52.8%	56.9%	Wales
1.1	Hodgkin lymphoma (all)	82.5%	79.3%	85.3%	82.3%	78.9%	85%	Wales
1.2	Mature B-cell neoplasms	66.5%	65%	67.9%	69.2%	67.5%	70.8%	Wales
1.3	Mature T-cell and NK-cell neoplasms	46.4%	40.5%	52.1%	50.6%	43.6%	57.1%	Wales
1.4	Unknown lymphoid neoplasms	48.4%	43.1%	53.4%	53.8%	48.2%	59.1%	Wales
2.5	Unknown myeloid neoplasms							Wales

S9 Table: 5-year age-standardised survival rates and 95% CI by deprivation in three nations for Figure 2

HAEMACARE group number	Blood Cancer subtype	Least deprived			4			3			2			Most deprived			Country
		5-year-survival	Lower 95% CI	Upper 95% CI	5-year-survival	Lower 95% CI	Upper 95% CI	5-year-survival	Lower 95% CI	Upper 95% CI	5-year-survival	Lower 95% CI	Upper 95% CI	5-year-survival	Lower 95% CI	Upper 95% CI	
All	All blood cancer	65.2%	64.8%	65.5%	64.2%	63.8%	64.6%	62.7%	62.3%	63.1%	60.2%	59.7%	60.7%	58.0%	57.5%	58.5%	England
1	Lymphoma, NOS	54.9%	51.3%	58.3%	55.6%	52.0%	59.1%	52.0%	48.2%	55.6%	46.0%	42.3%	49.6%	40.1%	36.0%	44.1%	England
2	NH lymphoma, NOS	66.2%	64.0%	68.3%	66.6%	64.4%	68.7%	63.3%	60.9%	65.5%	60.8%	58.2%	63.2%	55.7%	53.0%	58.4%	England
3	Composite Hodgkin and non-HL	74.5%	54.5%	86.7%	57.8%	35.6%	74.8%	68.0%	41.8%	84.3%	67.6%	48.7%	80.8%				England
4	HL, nodular lymphocyte predominance	95.9%	91.0%	98.1%	96.0%	91.2%	98.2%	95.2%	90.3%	97.7%	91.4%	87.2%	94.3%	99.2%	30.1%	100.0%	England

5	Classical HL	86.7%	85.7%	87.7%	85.6%	84.4%	86.6%	85.0%	83.8%	86.1%	83.5%	82.3%	84.6%	81.3%	79.9%	82.6%	England
6	Chronic lymphocytic leukaemia	84.3%	83.3%	85.3%	83.7%	82.6%	84.8%	82.2%	81.1%	83.4%	80.2%	78.9%	81.4%	78.4%	76.9%	79.9%	England
7	Immunoproliferative diseases	85.7%	83.4%	87.7%	83.7%	81.1%	86.0%	83.6%	80.9%	86.0%	78.7%	75.3%	81.7%	75.6%	70.8%	79.7%	England
8	Mantle cell/ centrocytic	61.1%	58.0%	64.1%	60.4%	57.3%	63.4%	58.3%	54.8%	61.6%	56.7%	53.0%	60.3%	48.9%	44.6%	52.9%	England
9	Follicular lymphoma	87.1%	85.6%	88.5%	85.5%	84.0%	86.9%	85.2%	83.5%	86.7%	81.4%	79.6%	83.1%	79.6%	77.5%	81.6%	England
10	Diffuse Large B-cell Lymphoma	63.9%	62.8%	64.9%	63.1%	62.0%	64.2%	61.9%	60.8%	63.0%	58.5%	57.2%	59.7%	56.3%	55.0%	57.6%	England
11	Burkitt's	51.0%	44.7%	56.9%	50.2%	43.7%	56.4%	43.9%	36.5%	51.0%	36.1%	29.4%	42.8%	35.7%	27.9%	43.6%	England
12	Marginal zone lymphoma	87.9%	85.9%	89.5%	88.6%	86.5%	90.4%	85.6%	83.5%	87.5%	84.2%	81.7%	86.4%	81.8%	79.0%	84.3%	England
13	T lymphoma cutaneous	81.4%	77.2%	85.0%	75.2%	70.7%	79.1%	79.5%	74.6%	83.6%	80.1%	74.4%	84.7%	71.9%	65.9%	76.9%	England
14	Other T cell lymphomas	43.4%	40.5%	46.2%	42.9%	39.9%	45.8%	41.4%	38.5%	44.4%	39.3%	36.4%	42.3%	34.8%	31.7%	38.0%	England
15	Lymphoblastic lymphoma/Acute (precursor cell) lymphatic leukaemia	56.8%	53.3%	60.1%	50.7%	46.9%	54.3%	53.4%	50.0%	56.8%	49.3%	45.6%	52.9%				England
16	Plasma cells neoplasms	58.3%	57.3%	59.4%	57.0%	55.9%	58.1%	56.6%	55.5%	57.7%	55.5%	54.4%	56.7%	51.9%	50.6%	53.2%	England
17	Mature B cell leukaemia																England
18	Mature B cell leukaemia, hairy cell	91.7%	86.0%	95.2%	90.6%	84.1%	94.5%	90.7%	84.0%	94.6%	83.3%	75.6%	88.8%	88.4%	76.8%	94.4%	England
19	Lymphatic leukaemia,NOS	68.3%	59.7%	75.5%	72.8%	62.7%	80.5%	64.9%	55.5%	72.7%	65.7%	52.6%	76.0%	53.9%	41.4%	64.8%	England
20	Leukaemia,NOS	38.9%	32.5%	45.3%	36.2%	29.1%	43.3%	36.7%	29.6%	43.8%	30.2%	22.7%	38.1%	35.7%	28.5%	43.0%	England
21	Myeloid leukaemia, NOS	45.0%	35.3%	54.1%	42.7%	33.5%	51.5%	42.1%	33.0%	50.9%	44.5%	34.9%	53.6%	40.7%	29.8%	51.3%	England
22	Acute myeloid leukaemia	24.9%	23.7%	26.1%	23.7%	22.5%	24.9%	22.0%	20.8%	23.1%	21.2%	20.0%	22.4%	20.9%	19.7%	22.2%	England
23	Myeloproliferative neoplasms	78.8%	77.5%	80.0%	79.2%	77.8%	80.5%	78.7%	77.2%	80.0%	75.7%	74.0%	77.2%	76.6%	74.8%	78.2%	England
24	Myelodysplastic syndrome	45.3%	43.4%	47.2%	41.9%	40.0%	43.9%	41.3%	39.5%	43.1%	38.2%	36.3%	40.1%	36.6%	34.6%	38.6%	England

25	Myelodysplastic/Myeloproliferative neoplasms	44.9%	41.3%	48.4%	44.0%	40.4%	47.5%	46.3%	42.9%	49.5%	43.1%	39.5%	46.6%	41.9%	38.3%	45.4%	England
26	Others	68.2%	61.4%	74.1%	62.0%	56.1%	67.4%	66.4%	60.0%	72.0%	68.6%	61.5%	74.7%	61.3%	54.1%	67.7%	England
1	All lymphoid malignancies	70.5%	70.0%	70.9%	69.7%	69.2%	70.2%	68.2%	67.7%	68.7%	65.6%	65.1%	66.1%	62.8%	62.2%	63.4%	England
2	All Myeloid malignancies	50.5%	49.7%	51.3%	49.2%	48.4%	50.0%	48.2%	47.4%	49.1%	46.1%	45.2%	47.0%	46.2%	45.2%	47.1%	England
1.1	Hodgkin lymphoma (all)	87.5%	86.4%	88.4%	86.5%	85.4%	87.5%	85.9%	84.8%	86.9%	84.3%	83.1%	85.4%	83.0%	81.7%	84.3%	England
1.2	Mature B-cell neoplasms	72.1%	71.6%	72.6%	71.3%	70.8%	71.8%	70.0%	69.4%	70.5%	67.3%	66.7%	67.9%	64.9%	64.3%	65.6%	England
1.3	Mature T-cell and NK-cell neoplasms	56.2%	53.8%	58.6%	54.9%	52.4%	57.3%	54.0%	51.4%	56.6%	52.8%	50.1%	55.4%	46.4%	43.6%	49.2%	England
1.4	Unknown lymphoid neoplasms	63.4%	61.5%	65.1%	63.7%	61.9%	65.5%	60.3%	58.4%	62.2%	56.7%	54.6%	58.7%	51.1%	48.9%	53.3%	England
2.5	Unknown myeloid neoplasms	41.4%	36.0%	46.6%	38.4%	32.8%	44.0%	39.0%	33.4%	44.5%	36.0%	29.9%	42.1%	37.6%	31.3%	43.9%	England
All	All blood cancer	68.7%	67.4%	69.9%	66.4%	65.1%	67.7%	64.7%	63.5%	66.0%	62.8%	61.5%	64.1%	62.3%	60.8%	63.7%	Scotland
1	Lymphoma, NOS				33.1%	21.0%	45.6%							27.0%	16.7%	38.5%	Scotland
2	NH lymphoma, NOS	65.5%	56.4%	73.2%	65.4%	55.1%	73.9%	67.9%	59.8%	74.7%	50.0%	40.6%	58.7%	48.0%	38.4%	57.1%	Scotland
3	Composite Hodgkin and non-HL																Scotland
4	HL, nodular lymphocyte predominance							89.8%	75.6%	96.0%	94.1%	63.0%	99.2%				Scotland
5	Classical HL	84.9%	80.9%	88.0%	86.5%	82.6%	89.6%	81.5%	77.5%	85.0%	80.8%	77.1%	84.0%	81.2%	76.5%	85.1%	Scotland
6	Chronic lymphocytic leukaemia	87.2%	82.7%	90.6%	85.0%	80.9%	88.3%	82.2%	78.4%	85.5%	82.6%	77.8%	86.5%	87.4%	82.3%	91.1%	Scotland
7	Immunoproliferative diseases	91.3%	82.6%	95.8%	83.6%	73.8%	90.0%							67.2%	52.4%	78.3%	Scotland
8	Mantle cell/ centrocytic	62.1%	53.8%	69.3%	60.6%	50.4%	69.4%	58.8%	48.1%	68.0%	57.8%	45.6%	68.2%	57.2%	44.2%	68.3%	Scotland
9	Follicular lymphoma	85.7%	79.7%	90.1%	85.1%	79.0%	89.6%	87.3%	82.0%	91.1%	87.0%	81.1%	91.1%	81.7%	74.9%	86.8%	Scotland
10	Diffuse Large B-cell Lymphoma	69.0%	65.3%	72.5%	65.9%	62.3%	69.2%	65.2%	61.6%	68.5%	58.1%	54.3%	61.7%	59.7%	55.5%	63.6%	Scotland

11	Burkitt's	54.1%	35.2%	69.6%							59.1%	40.6%	73.6%	56.8%	30.6%	76.3%	Scotland
12	Marginal zone lymphoma	84.9%	76.8%	90.3%	91.7%	81.8%	96.4%	87.3%	80.1%	92.0%	86.0%	76.8%	91.8%	84.8%	75.4%	90.8%	Scotland
13	T lymphoma cutaneous	72.1%	51.9%	85.0%	75.3%	57.2%	86.5%	77.7%	61.2%	87.8%	64.4%	48.4%	76.6%	69.6%	47.4%	83.8%	Scotland
14	Other T cell lymphomas	45.0%	36.1%	53.4%	36.6%	28.4%	44.9%	40.2%	31.8%	48.5%	41.9%	33.6%	50.0%	37.0%	29.0%	44.9%	Scotland
15	Lymphoblastic lymphoma/Acute (precursor cell) lymphatic leukaemia	49.6%	39.0%	59.4%	47.8%	33.9%	60.5%	52.2%	39.4%	63.5%							Scotland
16	Plasma cells neoplasms	58.3%	54.7%	61.7%	55.4%	51.5%	59.0%	52.8%	49.1%	56.3%	53.6%	49.8%	57.3%	49.3%	44.9%	53.6%	Scotland
17	Mature B cell leukaemia																Scotland
18	Mature B cell leukaemia, hairy cell																Scotland
19	Lymphatic leukaemia,NOS																Scotland
20	Leukaemia,NOS																Scotland
21	Myeloid leukaemia, NOS																Scotland
22	Acute myeloid leukaemia	24.0%	19.9%	28.3%	24.0%	20.3%	27.8%	21.7%	18.2%	25.4%	18.2%	14.7%	22.0%	20.9%	17.4%	24.5%	Scotland
23	Myeloproliferative neoplasms	87.0%	83.6%	89.7%	82.3%	78.5%	85.6%	82.7%	78.9%	85.8%	80.1%	76.1%	83.5%	80.6%	76.6%	84.1%	Scotland
24	Myelodysplastic syndrome	47.6%	40.9%	54.0%	46.6%	40.5%	52.4%	43.2%	37.7%	48.6%	40.9%	35.2%	46.5%	45.6%	40.7%	50.4%	Scotland
25	Myelodysplastic/Myeloproliferative neoplasms	51.0%	40.1%	60.9%	44.8%	36.4%	52.8%	43.7%	33.1%	53.8%	52.3%	44.0%	59.9%	46.3%	36.7%	55.3%	Scotland
26	Others	77.4%	72.0%	81.9%	82.9%	78.2%	86.7%	81.4%	76.7%	85.2%	78.7%	73.9%	82.8%	79.8%	74.6%	84.1%	Scotland
1	All lymphoid malignancies	70.7%	69.0%	72.2%	68.8%	67.2%	70.4%	67.4%	65.8%	68.9%	65.1%	63.4%	66.7%	64.2%	62.3%	66.0%	Scotland
2	All Myeloid malignancies	61.6%	59.2%	64.0%	57.1%	54.6%	59.5%	55.3%	52.9%	57.6%	54.0%	51.6%	56.4%	54.5%	52.0%	56.8%	Scotland
1.1	Hodgkin lymphoma (all)	85.6%	81.9%	88.5%	88.0%	84.2%	90.9%	82.4%	78.6%	85.6%	81.9%	78.3%	84.9%	83.0%	78.5%	86.6%	Scotland
1.2	Mature B-cell neoplasms	73.1%	71.3%	74.8%	71.1%	69.2%	72.8%	69.8%	68.0%	71.5%	68.0%	66.1%	69.8%	67.3%	65.2%	69.4%	Scotland

1.3	Mature T-cell and NK-cell neoplasms	56.2%	48.4%	63.3%	49.2%	41.8%	56.2%	52.4%	44.8%	59.5%	47.9%	40.3%	55.2%	45.2%	37.5%	52.7%	Scotland
1.4	Unknown lymphoid neoplasms	55.1%	48.1%	61.6%	55.0%	47.1%	62.2%	56.3%	49.5%	62.5%	40.8%	33.5%	47.9%	42.4%	34.8%	49.8%	Scotland
2.5	Unknown myeloid neoplasms																Scotland
All	All blood cancer	68.37%	65.73%	70.85%	66.22%	63.65%	68.66%	62.19%	59.55%	64.71%	61.27%	58.69%	63.75%	58.82%	55.92%	61.59%	Northern Ireland
1	Lymphoma, NOS	52.16%	30.50%	69.99%							45.88%	30.53%	59.94%				Northern Ireland
2	NH lymphoma, NOS										64.81%	44.23%	79.41%				Northern Ireland
3	Composite Hodgkin and non-HL																Northern Ireland
4	HL, nodular lymphocyte predominance										99.32%	0.00%	100.00%				Northern Ireland
5	Classical HL	87.84%	78.81%	93.19%	81.50%	74.49%	86.75%	84.00%	74.96%	89.99%	87.44%	80.58%	92.00%	85.34%	75.18%	91.56%	Northern Ireland
6	Chronic lymphocytic leukaemia	97.31%	72.93%	99.76%	91.50%	82.96%	95.86%				88.55%	78.21%	94.16%	74.55%	65.15%	81.77%	Northern Ireland
7	Immunoproliferative diseases																Northern Ireland
8	Mantle cell/ centrocytic													74.48%	46.90%	89.17%	Northern Ireland
9	Follicular lymphoma	88.19%	76.97%	94.14%	87.85%	77.08%	93.76%	79.66%	68.92%	87.02%	84.99%	71.56%	92.40%	77.16%	66.81%	84.65%	Northern Ireland
10	Diffuse Large B-cell Lymphoma	67.71%	60.57%	73.83%	65.81%	58.65%	72.03%	65.75%	58.39%	72.13%	57.99%	50.49%	64.76%	64.33%	56.22%	71.32%	Northern Ireland
11	Burkitt's																Northern Ireland

12	Marginal zone lymphoma	94.66%	56.81%	99.47%	88.22%	66.16%	96.27%	107.37%			90.41%	59.26%	98.08%	80.13%	51.43%	92.89%	Northern Ireland
13	T lymphoma cutaneous																Northern Ireland
14	Other T cell lymphomas	41.86%	24.94%	57.93%	34.66%	21.95%	47.69%	45.67%	30.63%	59.50%	35.25%	18.06%	52.99%				Northern Ireland
15	Lymphoblastic lymphoma/Acute (precursor cell) lymphatic leukaemia																Northern Ireland
16	Plasma cells neoplasms	63.35%	56.71%	69.26%	64.06%	57.39%	69.97%	56.19%	48.38%	63.27%	54.89%	47.93%	61.31%	47.82%	39.71%	55.47%	Northern Ireland
17	Mature B cell leukaemia																Northern Ireland
18	Mature B cell leukaemia, hairy cell	93.50%	2.65%	99.88%	84.86%	19.75%	98.35%				95.40%	0.00%	99.98%				Northern Ireland
19	Lymphatic leukaemia,NOS																Northern Ireland
20	Leukaemia,NOS																Northern Ireland
21	Myeloid leukaemia, NOS																Northern Ireland
22	Acute myeloid leukaemia	28.19%	21.54%	35.20%													Northern Ireland
23	Myeloproliferative neoplasms	77.92%	66.47%	85.87%	71.44%	62.65%	78.51%	71.65%	62.51%	78.93%	72.69%	63.17%	80.14%	77.60%	67.95%	84.67%	Northern Ireland
24	Myelodysplastic syndrome	51.10%	40.83%	60.46%				42.70%	34.05%	51.05%	34.16%	25.18%	43.33%	34.88%	25.06%	44.86%	Northern Ireland

25	Myelodysplastic/Myeloproliferative neoplasms																Northern Ireland
26	Others	63.18%	37.92%	80.46%	92.67%	44.45%	99.29%	82.41%	49.37%	94.83%							Northern Ireland
1	All lymphoid malignancies	74.9%	71.7%	77.8%	72.9%	69.8%	75.6%	67.6%	64.4%	70.6%	68.4%	65.1%	71.4%	64.2%	60.6%	67.5%	Northern Ireland
2	All Myeloid malignancies	1	0	1	0	0	1	0	0	1	0	0	1	0	0	1	Northern Ireland
1.1	Hodgkin lymphoma (all)	86.71%	79.11%	91.69%	84.05%	77.71%	88.73%	85.03%	76.69%	90.56%	88.72%	82.26%	92.92%	86.39%	76.35%	92.37%	Northern Ireland
1.2	Mature B-cell neoplasms	78.13%	74.63%	81.21%	75.36%	71.95%	78.43%	70.24%	66.55%	73.60%	70.66%	66.96%	74.03%	65.88%	61.83%	69.61%	Northern Ireland
1.3	Mature T-cell and NK-cell neoplasms	47.13%	30.70%	61.92%	46.73%	33.24%	59.13%	46.24%	32.03%	59.30%	42.58%	28.37%	56.07%				Northern Ireland
1.4	Unknown lymphoid neoplasms	66.26%	53.78%	76.09%	59.25%	44.00%	71.63%				54.48%	42.67%	64.84%	59.03%	43.22%	71.80%	Northern Ireland
2.5	Unknown myeloid neoplasms																Northern Ireland
All	All blood cancer	65.6%	63.9%	67.3%	63.1%	61.4%	64.7%	61.1%	59.4%	62.7%	54.8%	53%	56.5%	54.9%	52.9%	56.9%	Wales
1	Lymphoma, NOS										37%	21.8%	52.3%				Wales
2	NH lymphoma, NOS				61.3%	52.6%	68.8%	52.6%	43.4%	61.6%	48.7%	37.7%	58.8%				Wales
3	Composite Hodgkin and non-HL																Wales
4	HL, nodular lymphocyte predominance																Wales
5	Classical HL	85.8%	79.8%	90.2%	82.3%	77.2%	86.5%	82%	77.1%	85.9%				79%	73.5%	83.5%	Wales

6	Chronic lymphocytic leukaemia	88%	82.5%	91.9%				85.5%	80.1%	89.6%	77.3%	71.4%	82.1%	72.6%	67.1%	77.4%	Wales
7	Immunoproliferative diseases	76.7%	61.2%	86.7%				75.5%	61.9%	84.8%	74.1%	58.9%	84.4%	64.3%	48.7%	76.2%	Wales
8	Mantle cell/ centrocytic				59.7%	45.9%	71.1%	63.8%	52.4%	73.2%							Wales
9	Follicular lymphoma	84.4%	76.2%	90%	84.4%	75.8%	90.1%	78.1%	69.8%	84.4%	73.2%	64.3%	80.2%	79.5%	69.3%	86.6%	Wales
10	Diffuse Large B-cell Lymphoma	67.1%	62.2%	71.5%	62.3%	57.5%	66.7%	61.8%	57.1%	66.2%	52.3%	47.4%	57%	54.2%	48.9%	59.2%	Wales
11	Burkitt's																Wales
12	Marginal zone lymphoma	91.3%	75.8%	97.1%				82.6%	65.9%	91.6%	73.9%	62%	82.6%	78.1%	64.4%	87.1%	Wales
13	T lymphoma cutaneous	68.3%	47.6%	82.2%													Wales
14	Other T cell lymphomas							30.2%	18.4%	42.9%	40.4%	28.4%	52.1%	37.7%	24.1%	51.2%	Wales
15	Lymphoblastic lymphoma/Acute (precursor cell) lymphatic leukaemia																Wales
16	Plasma cells neoplasms	59.4%	54.7%	63.8%	54.5%	50%	58.8%	58.8%	53.9%	63.4%	49.3%	44.3%	54.2%	50.9%	45.8%	55.8%	Wales
17	Mature B cell leukaemia																Wales
18	Mature B cell leukaemia, hairy cell																Wales
19	Lymphatic leukaemia,NOS																Wales
20	Leukaemia,NOS																Wales
21	Myeloid leukaemia, NOS																Wales
22	Acute myeloid leukaemia	29.4%	24.6%	34.3%	22.6%	18%	27.5%	19%	15.1%	23.2%				25.7%	21.3%	30.4%	Wales
23	Myeloproliferative neoplasms	77.1%	72.3%	81.1%	74.9%	70.2%	79%	75.3%	70.5%	79.4%	72.2%	67.6%	76.3%	67.5%	61.2%	73.1%	Wales
24	Myelodysplastic syndrome	41.8%	34.5%	49%	43.8%	36.7%	50.7%	36%	28.3%	43.7%				37.4%	30.1%	44.7%	Wales

25	Myelodysplastic/Myeloproliferative neoplasms										30.7%	21.8%	40.1%				Wales
26	Others							65.2%	51.8%	75.8%				67.8%	54.3%	78.1%	Wales
1	All lymphoid malignancies	71.7%	69.5%	73.7%	67.9%	65.8%	70%	67.8%	65.6%	69.8%	58.9%	56.6%	61.1%	58.5%	56%	60.8%	Wales
2	All Myeloid malignancies	54.2%	51.3%	57%	53.3%	50.3%	56.2%	48.7%	45.9%	51.4%	47.7%	44.8%	50.5%	48.6%	45.2%	51.8%	Wales
1.1	Hodgkin lymphoma (all)	87%	81.5%	91%	84.4%	79.3%	88.4%	82.3%	78.1%	86.4%	78.2%	72.3%	82.9%	79.9%	74.6%	84.2%	Wales
1.2	Mature B-cell neoplasms	73%	70.6%	75.3%	69.8%	67.4%	72.1%	70.6%	68.2%	72.9%	61.2%	58.6%	63.7%	61.3%	58.6%	63.8%	Wales
1.3	Mature T-cell and NK-cell neoplasms	53%	42.9%	62.2%							41.3%	32%	50.4%				Wales
1.4	Unknown lymphoid neoplasms				56%	48.4%	62.9%	49.7%	41.9%	57%	45.5%	36.7%	53.9%	35.7%	27.2%	44.3%	Wales
2.5	Unknown myeloid neoplasms																Wales

S10a Table: 5-year age-standardised survival rates and 95% CI by ethnicity (6 groups) in England for Figure S3

HAEMACARE group number	Blood Cancer subtype	White			Mixed			Asian			Black			Others			Unknown		
		5-year-survival	Lower 95% CI	Upper 95% CI	5-year-survival	Lower 95% CI	Upper 95% CI	5-year-survival	Lower 95% CI	Upper 95% CI	5-year-survival	Lower 95% CI	Upper 95% CI	5-year-survival	Lower 95% CI	Upper 95% CI	5-year-survival	Lower 95% CI	Upper 95% CI
All	All blood cancer	62.1%	61.9%	62.3%	67.5%	63.9%	70.8%	62.8%	61.6%	64.0%	62.9%	61.3%	64.4%	65.1%	63.1%	67.1%	69.1%	67.9%	70.2%
1	Lymphoma, NOS	51.4%	49.6%	53.2%	57.1%	27.9%	78.2%	50.1%	41.3%	58.3%	52.3%	40.3%	63.0%				40.9%	29.7%	51.8%
2	NH lymphoma, NOS	63.1%	62.0%	64.3%	69.8%	45.4%	84.9%	63.0%	57.1%	68.3%	59.2%	49.3%	67.8%	56.4%	47.3%	64.5%	68.6%	62.5%	73.9%
3	Composite Hodgkin and non-HL	61.2%	51.2%	69.8%															
4	HL, nodular lymphocyte predominance	95.0%	92.9%	96.5%				96.9%	70.0%	99.7%	100.1%			91.6%	69.8%	97.9%			

5	Classical HL	84.2 %	83.6 %	84.8 %	92.9 %	82.2 %	97.3 %	84.0 %	81.5 %	86.3 %	85.8 %	81.1 %	89.4 %	88.4 %	83.1 %	92.1 %	88.5 %	85.5 %	90.9 %
6	Chronic lymphocytic leukaemia	81.8 %	81.2 %	82.3 %	74.9 %	63.1 %	83.4 %	84.0 %	79.7 %	87.5 %	79.2 %	72.4 %	84.5 %	84.0 %	77.2 %	89.0 %	89.1 %	85.9 %	91.6 %
7	Immunoproliferative diseases	82.0 %	80.7 %	83.3 %				87.9 %	75.4 %	94.3 %	81.0 %	62.5 %	91.0 %	89.2 %	67.6 %	96.7 %	88.5 %	80.1 %	93.5 %
8	Mantle cell/ centrocytic	58.1 %	56.5 %	59.6 %										63.3 %	46.2 %	76.2 %	60.3 %	51.1 %	68.3 %
9	Follicular lymphoma	84.1 %	83.3 %	84.8 %	96.2 %	44.7 %	99.8 %	87.8 %	81.7 %	91.9 %	89.4 %	76.1 %	95.5 %	88.2 %	77.5 %	94.0 %	84.4 %	79.3 %	88.4 %
10	Diffuse Large B-cell Lymphoma	61.3 %	60.8 %	61.9 %	60.0 %	50.4 %	68.3 %	55.2 %	52.4 %	58.0 %	60.3 %	54.9 %	65.2 %	64.4 %	58.8 %	69.5 %	61.8 %	58.5 %	65.0 %
11	Burkitt's	44.3 %	41.1 %	47.5 %				44.0 %	28.1 %	58.9 %							58.8 %	34.2 %	77.0 %
12	Marginal zone lymphoma	85.6 %	84.6 %	86.6 %	94.0 %	27.3 %	99.7 %	88.0 %	80.7 %	92.7 %	82.3 %	67.8 %	90.7 %	91.8 %	71.4 %	97.8 %	95.0 %	85.9 %	98.3 %
13	T lymphoma cutaneous	76.8 %	74.5 %	78.9 %				90.4 %	67.8 %	97.4 %	73.4 %	57.4 %	84.2 %				87.1 %	62.7 %	96.0 %
14	Other T cell lymphomas	41.1 %	39.7 %	42.6 %	38.5 %	19.4 %	57.3 %	47.7 %	39.5 %	55.5 %	20.8 %	16.3 %	25.7 %	36.7 %	24.7 %	48.7 %	49.8 %	41.5 %	57.5 %
15	Lymphoblastic lymphoma/Acute (precursor cell) lymphatic leukaemia	52.1 %	50.3 %	53.8 %															
16	Plasma cells neoplasms	55.4 %	54.8 %	55.9 %	59.2 %	51.6 %	66.0 %	59.6 %	56.7 %	62.3 %	66.1 %	63.5 %	68.5 %	61.5 %	56.7 %	65.9 %	57.5 %	54.1 %	60.7 %
17	Mature B cell leukaemia	60.4 %	45.3 %	72.6 %															
18	Mature B cell leukaemia, hairy cell	89.3 %	86.4 %	91.6 %				90.1 %	44.3 %	98.7 %							80.4 %	53.6 %	92.6 %
19	Lymphatic leukaemia,NOS	65.9 %	61.0 %	70.4 %															
20	Leukaemia,NOS	36.2 %	32.8 %	39.6 %															
21	Myeloid leukaemia, NOS	43.8 %	39.1 %	48.3 %				64.3 %	35.6 %	82.7 %									
22	Acute myeloid leukaemia	22.6 %	22.0 %	23.1 %	31.1 %	21.0 %	41.7 %	25.4 %	22.4 %	28.5 %	24.8 %	20.6 %	29.2 %	26.5 %	21.9 %	31.3 %			
23	Myeloproliferative neoplasms	77.2 %	76.6 %	77.9 %	87.8 %	64.5 %	96.2 %	85.8 %	81.6 %	89.1 %	86.4 %	79.9 %	90.9 %	80.0 %	72.3 %	85.8 %	88.6 %	84.4 %	91.7 %

24	Myelodysplastic syndrome	40.2%	39.3%	41.1%	57.9%	41.7%	71.1%	43.6%	39.0%	48.1%	47.2%	40.2%	53.8%	38.5%	30.2%	46.8%	52.3%	46.6%	57.7%
25	Myelodysplastic/Myeloproliferative neoplasms	42.7%	41.0%	44.4%				49.9%	40.9%	58.2%	55.1%	42.5%	66.0%	49.5%	34.6%	62.7%	56.0%	46.3%	64.6%
26	Others	65.9%	62.8%	68.8%				57.4%	41.2%	70.6%							74.5%	58.4%	85.1%
1	All lymphoid malignancies	67.7%	67.4%	67.9%	69.8%	65.7%	73.4%	66.2%	64.8%	67.6%	65.6%	63.8%	67.4%	70.0%	67.5%	72.3%	73.3%	71.9%	74.6%
2	All Myeloid malignancies	47.4%	47.0%	47.8%	59.2%	51.3%	66.3%	53.5%	51.2%	55.7%	54.2%	51.0%	57.3%	51.2%	47.4%	54.8%	57.4%	55.0%	59.6%
1.1	Hodgkin lymphoma (all)	85.1%	84.5%	85.6%	94.4%	83.7%	98.1%	85.8%	83.5%	87.9%	88.6%	84.2%	91.8%	88.8%	84.0%	92.3%	88.9%	86.2%	91.2%
1.2	Mature B-cell neoplasms	69.4%	69.1%	69.6%	70.0%	65.5%	74.0%	67.7%	66.1%	69.2%	68.8%	66.8%	70.7%	72.7%	70.0%	75.2%	75.1%	73.5%	76.5%
1.3	Mature T-cell and NK-cell neoplasms	53.1%	51.8%	54.3%	43.6%	26.5%	59.5%	59.6%	51.9%	66.5%	33.9%	28.7%	39.2%	44.3%	32.3%	55.5%	62.2%	55.0%	68.6%
1.4	Unknown lymphoid neoplasms	60.1%	59.2%	61.0%	66.1%	50.0%	78.1%	59.5%	54.7%	64.0%	57.7%	50.3%	64.3%	56.3%	48.8%	63.1%	61.8%	56.7%	66.5%
2.5	Unknown myeloid neoplasms	39.1%	36.4%	41.8%				42.0%	26.3%	57.0%									

S10b Table: 5-year age-standardised survival rates and 95% CI by ethnicity (4 groups) in England for Figure 6

HAEMACARE group number	Blood Cancer subtype	White				Non-White				Others				Unknow			
		n	5-year survival (%)	Lower 95% CI	Upper 95% CI	n	5-year survival (%)	Lower 95% CI	Upper 95% CI	n	5-year survival (%)	Lower 95% CI	Upper 95% CI	n	5-year survival (%)	Lower 95% CI	Upper 95% CI
All	All blood cancer	302,772	62.12%	61.91%	62.33%	20,645	63.2%	62.3%	64.1%	4,300	65.14%	63.10%	67.11%		69.11%	67.94%	70.25%
1	Lymphoma, NOS	4,314	51.4%	49.6%	53.2%	295	50.9%	44.0%	57.3%	47				112	40.94%	29.71%	51.84%
2	NH lymphoma, NOS	10,877	63.1%	62.0%	64.3%	710	62.6%	57.8%	67.0%	149	56.37%	47.32%	64.46%	381	68.58%	62.55%	73.85%
3	Composite Hodgkin and non-HL	175	61.2%	51.2%	69.8%	25				4				6			
4	HL, nodular lymphocyte predominance	1,282	95.0%	92.9%	96.5%	325	99.6%	0.0%	100.0%	39	91.59%	69.78%	97.88%	83			

5	Classical HL	13,408	84.2%	83.6%	84.8%	1709	84.9%	82.8%	86.8%	369	88.42%	83.13%	92.13%	600	88.47%	85.51%	90.86%
6	Chronic lymphocytic leukaemia	36,767	81.8%	81.2%	82.3%	1337	82.1%	78.7%	84.9%	408	84.03%	77.21%	88.95%	1,568	89.12%	85.92%	91.63%
7	Immunoproliferative diseases	6,779	82.0%	80.7%	83.3%	260	87.2%	78.3%	92.7%	61	89.21%	67.61%	96.73%	231	88.47%	80.05%	93.48%
8	Mantle cell/ centrocytic	5,139	58.1%	56.5%	59.6%	150	50.9%	40.9%	60.0%	63	63.25%	46.24%	76.18%	166	60.30%	51.14%	68.28%
9	Follicular lymphoma	21,040	84.1%	83.3%	84.8%	1106	88.9%	84.0%	92.4%	296	88.24%	77.55%	94.03%	691	84.43%	79.31%	88.38%
10	Diffuse Large B-cell Lymphoma	42,966	61.3%	60.8%	61.9%	2871	56.6%	54.2%	59.0%	605	64.41%	58.75%	69.51%	1,187	61.82%	58.49%	64.97%
11	Burkitt's	1,392	44.3%	41.1%	47.5%	162	43.4%	29.1%	56.9%	35				44	58.84%	34.15%	76.96%
12	Marginal zone lymphoma	10,982	85.6%	84.6%	86.6%	726	86.2%	79.9%	90.6%	165	91.76%	71.41%	97.83%	379	95.02%	85.90%	98.30%
13	T lymphoma cutaneous	3,079	76.8%	74.5%	78.9%	346	78.2%	66.5%	86.1%	45				118	87.06%	62.68%	95.97%
14	Other T cell lymphomas	6,091	41.1%	39.7%	42.6%	689	32.4%	28.0%	36.9%	114	36.66%	24.67%	48.70%	199	49.75%	41.50%	57.46%
15	Lymphoblastic lymphoma/Acute (precursor cell) lymphatic leukaemia	3,162	52.1%	50.3%	53.8%	417				84				73			
16	Plasma cells neoplasms	45,514	55.4%	54.8%	55.9%	4446	63.1%	61.3%	64.9%	642	61.47%	56.71%	65.87%	1,218	57.52%	54.15%	60.73%
17	Mature B cell leukaemia	63	60.4%	45.3%	72.6%	4				2				1			
18	Mature B cell leukaemia, hairy cell	1,923	89.3%	86.4%	91.6%	69	92.7%	50.2%	99.2%	37				85	80.39%	53.60%	92.65%
19	Lymphatic leukaemia,NOS	584	65.9%	61.0%	70.4%	21	69.3%	46.5%	83.9%	12				18			
20	Leukaemia,NOS	1,166	36.2%	32.8%	39.6%	70				10				30			
21	Myeloid leukaemia, NOS	786	43.8%	39.1%	48.3%	42	52.2%	34.3%	67.3%	9				23			
22	Acute myeloid leukaemia	25,030	22.6%	22.0%	23.1%	1543	25.6%	23.1%	28.1%	357	26.49%	21.86%	31.33%	696			
23	Myeloproliferative neoplasms	26,273	77.2%	76.6%	77.9%	1899	86.2%	82.9%	89.0%	459	79.99%	72.30%	85.75%	1,052	88.59%	84.38%	91.72%
24	Myelodysplastic syndrome	26,088	40.2%	39.3%	41.1%	1030	45.6%	41.9%	49.2%	207	38.53%	30.19%	46.78%	539	52.30%	46.64%	57.65%

25	Myelodysplastic/Myeloproliferative neoplasms	6,217	42.7%	41.0%	44.4%	270	53.6%	46.4%	60.3%	67	49.49%	34.61%	62.73%	166	55.97%	46.28%	64.59%
26	Others	1,675	65.9%	62.8%	68.8%	123	56.0%	43.4%	66.9%	14				55	74.46%	58.41%	85.06%
1	All lymphoid malignancies	215,362	67.7%	67.4%	67.9%	15,643	66%	65%	67%	3,173	70.0%	67.6%	72.3%	7,154	73.3%	71.9%	74.6%
2	All Myeloid malignancies	85,560	47.4%	47.0%	47.8%	4,854	54%	52%	56%	1,109	1	0	1	2,506	1	1	1
1.1	Hodgkin lymphoma (all)	14,690	85.1%	84.5%	85.6%	2034	87%	85%	89%	408	88.84%	83.98%	92.30%	683	88.94%	86.15%	91.20%
1.2	Mature B-cell neoplasms	172,565	69.4%	69.1%	69.6%	11131	68%	67%	70%	2,314	72.71%	69.97%	75.24%	5,570	75.05%	73.52%	76.51%
1.3	Mature T-cell and NK-cell neoplasms	9,170	53.1%	51.8%	54.3%	1035	45%	40%	49%	159	44.26%	32.33%	55.53%	317	62.23%	54.99%	68.65%
1.4	Unknown lymphoid neoplasms	15,775	60.1%	59.2%	61.0%	1026	59%	55%	63%	208	56.26%	48.79%	63.07%	511	61.79%	56.67%	66.48%
2.5	Unknown myeloid neoplasms	1,952	39.1%	36.4%	41.8%	112	34%	24%	45%	19				53			

S11 Table: 5-year age-standardised survival rates and 95% CI by rurality in Northern Ireland, Scotland and Wales for Figure S4

HAEMACARE group number	Blood Cancer subtype	Urban				Mixed				Rural				country
		n	5-year-survival	Lower 95% CI	Upper 95% CI	n	5-year-survival	Lower 95% CI	Upper 95% CI	n	5-year-survival	Lower 95% CI	Upper 95% CI	
All	All blood cancer	30,554	64.8%	64.1%	65.4%	4,869	65.6%	64.0%	67.2%	2,704	66.0%	63.8%	68.2%	Scotland
1	Lymphoma, NOS	380	23.9%	17.8%	30.5%									Scotland
2	NH lymphoma, NOS	578	59.2%	54.4%	63.7%	113	67.4%	56.3%	76.2%	60	59.9%	44.0%	72.7%	Scotland
3	Composite Hodgkin and non-HL													Scotland
4	HL, nodular lymphocyte predominance	137	99.0%	17.9%	100.0%									Scotland
5	Classical HL	1,378	83.2%	81.3%	84.9%	182	82.6%	76.6%	87.1%	98	76.0%	66.3%	83.3%	Scotland
6	Chronic lymphocytic leukaemia	2,874	84.8%	82.6%	86.7%	506	87.4%	82.2%	91.2%	261	80.8%	73.4%	86.4%	Scotland
7	Immunoproliferative diseases	563	77.8%	72.7%	82.0%									Scotland
8	Mantle cell/ centrocytic	424	58.1%	52.9%	63.0%	85	62.8%	47.3%	74.9%					Scotland

9	Follicular lymphoma	1,989	85.5%	82.7%	87.9%	302	84.7%	77.3%	89.8%	194	88.2%	78.8%	93.6%	Scotland
10	Diffuse Large B-cell Lymphoma	3,852	63.5%	61.6%	65.3%	645	61.8%	57.2%	66.1%	372	67.4%	60.8%	73.1%	Scotland
11	Burkitt's	171	49.7%	40.7%	58.1%	24	74.8%	36.6%	92.0%	13				Scotland
12	Marginal zone lymphoma	876	86.4%	82.6%	89.4%	137	91.2%	75.0%	97.1%	89	87.8%	74.2%	94.5%	Scotland
13	T lymphoma cutaneous	288	69.1%	60.2%	76.4%	55	74.6%	56.0%	86.2%	29	104.6%			Scotland
14	Other T cell lymphomas	666	39.3%	35.2%	43.4%	94	43.9%	32.7%	54.4%	57	36.9%	24.6%	49.2%	Scotland
15	Lymphoblastic lymphoma/Acute (precursor cell) lymphatic leukaemia	295	49.5%	43.9%	54.9%	37	50.2%	29.4%	67.8%					Scotland
16	Plasma cells neoplasms	3,890	54.3%	52.4%	56.2%	702	52.7%	47.9%	57.3%	389	52.8%	46.6%	58.6%	Scotland
17	Mature B cell leukaemia													Scotland
18	Mature B cell leukaemia, hairy cell													Scotland
19	Lymphatic leukaemia,NOS													Scotland
20	Leukaemia,NOS													Scotland
21	Myeloid leukaemia, NOS													Scotland
22	Acute myeloid leukaemia	1,950	21.0%	19.1%	22.9%	329	20.6%	16.4%	25.0%	171	31.8%	25.1%	38.6%	Scotland
23	Myeloproliferative neoplasms	3,896	82.6%	80.8%	84.3%	543	83.7%	78.4%	87.7%	340	79.2%	73.0%	84.1%	Scotland
24	Myelodysplastic syndrome	2,959	44.1%	41.2%	46.9%	442	47.6%	40.6%	54.2%	260	47.0%	37.6%	55.9%	Scotland
25	Myelodysplastic/Myeloproliferative neoplasms	728	46.4%	41.6%	51.2%									Scotland
26	Others	2,476	79.8%	77.4%	81.9%	374	79.1%	73.2%	83.8%	158	85.9%	75.8%	92.0%	Scotland
1	All lymphoid malignancies	18,490	67.08%	66.23%	67.91%	3,085	68.37%	66.30%	70.34%	1,717	67.46%	64.65%	70.10%	Scotland
2	All Myeloid malignancies	9,588	56.3%	55.0%	57.4%	1,410	55.6%	52.5%	58.7%	829	59.3%	55.2%	63.1%	Scotland
1.1	Hodgkin lymphoma (all)	1,515	84.47%	82.65%	86.11%	196	83.7%	78.0%	88.0%	111	77.7%	68.9%	84.2%	Scotland
1.2	Mature B-cell neoplasms	14,743	69.82%	68.89%	70.74%	2,523	70.4%	68.1%	72.6%	1,403	70.2%	67.1%	73.0%	Scotland
1.3	Mature T-cell and NK-cell neoplasms	954	48.35%	44.56%	52.03%	149	55.1%	45.3%	63.8%	86	59.1%	45.1%	70.6%	Scotland
1.4	Unknown lymphoid neoplasms	969	48.27%	44.44%	51.99%	179	55.9%	47.2%	63.7%	90	51.7%	39.0%	62.9%	Scotland
2.5	Unknown myeloid neoplasms													Scotland

**All**	**All blood cancer**	**6,063**	**64.70%**	**62.66%**	**66.66%**	**935**	**62.74%**	**61.17%**	**64.27%**	**3,441**	**64.80%**	**60.82%**	**68.48%**	**Northern Ireland**
**1**	**Lymphoma, NOS**	**161**				**29**	**47.72%**	**38.34%**	**56.51%**	**57**				**Northern Ireland**
**2**	**NH lymphoma, NOS**	**135**	**72.61%**	**56.50%**	**83.58%**	**24**				**58**				**Northern Ireland**
**3**	**Composite Hodgkin and non-HL**													**Northern Ireland**
**4**	**HL, nodular lymphocyte predominance**	**57**	**99.03%**	**0.00%**	**100.00%**	**6**	**92.58%**	**81.93%**	**97.06%**	**21**				**Northern Ireland**
**5**	**Classical HL**	**313**	**85.78%**	**79.58%**	**90.21%**	**42**	**83.77%**	**79.26%**	**87.37%**	**189**				**Northern Ireland**
**6**	**Chronic lymphocytic leukaemia**	**688**	**86.89%**	**79.75%**	**91.65%**	**106**	**87.81%**	**82.99%**	**91.33%**	**432**	**89.53%**	**73.61%**	**96.09%**	**Northern Ireland**
**7**	**Immunoproliferative diseases**	**98**				**17**				**47**				**Northern Ireland**
**8**	**Mantle cell/ centrocytic**	**79**	**47.42%**	**32.32%**	**61.09%**	**8**				**56**				**Northern Ireland**
**9**	**Follicular lymphoma**	**462**	**83.27%**	**74.74%**	**89.12%**	**76**	**83.93%**	**78.11%**	**88.31%**	**305**	**91.00%**	**64.12%**	**98.02%**	**Northern Ireland**
**10**	**Diffuse Large B-cell Lymphoma**	**823**	**64.10%**	**58.65%**	**69.02%**	**124**	**64.06%**	**59.79%**	**68.00%**	**447**	**70.58%**	**58.48%**	**79.74%**	**Northern Ireland**
**11**	**Burkitt's**	**24**								**7**				**Northern Ireland**
**12**	**Marginal zone lymphoma**	**156**	**94.00%**	**64.96%**	**99.12%**	**25**	**91.17%**	**78.07%**	**96.60%**	**79**				**Northern Ireland**
**13**	**T lymphoma cutaneous**	**40**	**90.58%**	**2.26%**	**99.74%**	**10**	**67.55%**	**44.59%**	**82.65%**	**21**				**Northern Ireland**
**14**	**Other T cell lymphomas**	**163**	**31.91%**	**20.10%**	**44.34%**	**20**	**38.62%**	**29.90%**	**47.25%**	**66**				**Northern Ireland**
**15**	**Lymphoblastic lymphoma/Acute (precursor cell) lymphatic leukaemia**	**133**				**23**				**96**				**Northern Ireland**
**16**	**Plasma cells neoplasms**	**862**	**58.25%**	**52.74%**	**63.34%**	**149**	**56.66%**	**52.26%**	**60.82%**	**523**				**Northern Ireland**
**17**	**Mature B cell leukaemia**													**Northern Ireland**
**18**	**Mature B cell leukaemia, hairy cell**	**35**	**120.88%**							**17**				**Northern Ireland**
**19**	**Lymphatic leukaemia,NOS**						**74.02%**	**8.47%**	**96.40%**		**117.91%**			**Northern Ireland**

20	Leukaemia,NOS	64								24				Northern Ireland
21	Myeloid leukaemia, NOS	20								10				Northern Ireland
22	Acute myeloid leukaemia	423	25.86%	20.47%	31.57%	88	21.72%	17.52%	26.21%	264				Northern Ireland
23	Myeloproliferative neoplasms	582	75.62%	68.06%	81.63%	76	73.93%	68.69%	78.44%	326	79.48%	64.42%	88.70%	Northern Ireland
24	Myelodysplastic syndrome	580	44.33%	36.67%	51.70%	82	37.50%	31.26%	43.73%	318				Northern Ireland
25	Myelodysplastic/Myeloproliferative neoplasms	73				6				29				Northern Ireland
26	Others	81	82.07%	60.63%	92.49%	11	62.94%	47.52%	74.96%	48				Northern Ireland
1	All lymphoid malignancies	4,240	70.2%	67.7%	72.5%	669	69.3%	67.4%	71.1%	2,422	72.8%	67.8%	77.2%	Northern Ireland
2	All Myeloid malignancies	1,742	50.3%	46.5%	54.0%	255	47.0%	44.1%	49.9%	971	43.5%	36.5%	50.3%	Northern Ireland
1.1	Hodgkin lymphoma (all)	370	87.0%	81.2%	91.1%	48	85.0%	81.0%	88.2%	210	87.7%	76.7%	93.8%	Northern Ireland
1.2	Mature B-cell neoplasms	3,230	72.3%	69.5%	74.8%	512	72.2%	70.1%	74.2%	1,913	75.3%	69.7%	80.1%	Northern Ireland
1.3	Mature T-cell and NK-cell neoplasms	203	42.2%	30.0%	53.9%	30	43.9%	35.7%	51.8%	87	51.0%	31.5%	67.6%	Northern Ireland
1.4	Unknown lymphoid neoplasms	299	57.3%	46.3%	66.9%	55	59.9%	52.9%	66.1%	116				Northern Ireland
2.5	Unknown myeloid neoplasms	84	67.3%	53.2%	78.0%					34				Northern Ireland
All	All blood cancer	14,580	59.00%	58.00%	60.00%	4,130	59.00%	57.10%	60.80%	3,850	65.70%	63.80%	67.50%	Wales
1	Lymphoma, NOS	220				60	40.10%	24.00%	55.70%	50	31.70%	17.10%	47.30%	Wales
2	NH lymphoma, NOS	470	52.10%	46.60%	57.30%	130	60.70%	48.40%	70.90%	130	60.30%	50.90%	68.50%	Wales
3	Composite Hodgkin and non-HL	<20				<20				<20				Wales
4	HL, nodular lymphocyte predominance	60				<20				<20				Wales
5	Classical HL	580	81.50%	78.50%	84.00%	150	78.80%	72.30%	84.00%	150				Wales
6	Chronic lymphocytic leukaemia	1,660	81.80%	78.80%	84.40%	470	79.80%	73.50%	84.70%	480				Wales

7	Immunoproliferative diseases	240	72.70%	64.80%	79.10%	70	75.30%	59.70%	85.60%	70				Wales
8	Mantle cell/ centrocytic	210	56.50%	48.90%	63.30%	70				80				Wales
9	Follicular lymphoma	740	80.90%	76.20%	84.80%	240	75.20%	66.60%	81.90%	260	83.60%	74.30%	89.80%	Wales
10	Diffuse Large B-cell Lymphoma	1,840	57.50%	54.80%	60.10%	510				500	63.40%	57.90%	68.40%	Wales
11	Burkitt's	60				<20				<20				Wales
12	Marginal zone lymphoma	410	83.00%	77.20%	87.40%	100	73.30%	57.50%	84.10%	90				Wales
13	T lymphoma cutaneous	110	74.40%	62.20%	83.20%	30				30				Wales
14	Other T cell lymphomas	230	39.60%	32.50%	46.70%	60	35.40%	22.60%	48.50%	50				Wales
15	Lymphoblastic lymphoma/Acute (precursor cell) lymphatic leukaemia	140				40				40				Wales
16	Plasma cells neoplasms	1,840	54.20%	51.50%	56.80%	570	54.00%	49.00%	58.80%	510	58.70%	53.70%	63.30%	Wales
17	Mature B cell leukaemia	<20				<20				<20				Wales
18	Mature B cell leukaemia, hairy cell	70	76.40%	58.50%	87.30%	30				<20				Wales
19	Lymphatic leukaemia,NOS	40	64.10%	44.80%	78.20%	<20				<20				Wales
20	Leukaemia,NOS	60				<20				<20				Wales
21	Myeloid leukaemia, NOS	<20				<20				<20				Wales
22	Acute myeloid leukaemia	1,260	21.60%	19.20%	24.10%	340	24.10%	19.40%	29.20%	320	29.50%	24.20%	35.00%	Wales
23	Myeloproliferative neoplasms	2,260	72.20%	69.50%	74.70%	630	69.00%	64.00%	73.40%	500	83.80%	78.00%	88.20%	Wales
24	Myelodysplastic syndrome	1,580	37.70%	33.50%	41.80%	440	36.00%	29.00%	43.00%	420	39.90%	32.00%	47.60%	Wales
25	Myelodysplastic/Myeloproliferative neoplasms	220	23.60%	16.40%	31.60%	90				60				Wales
26	Others	270	66.90%	59.40%	73.40%	70				50				Wales
1	All lymphoid malignancies	8,920	64.30%	63.10%	65.50%	2,530	64.80%	62.40%	67.10%	2,480	69.70%	67.30%	72.00%	Wales

2	All Myeloid malignancies	5,390	49.50%	47.90%	51.10%	1,530	48.30%	45.30%	51.30%	1,330	57.10%	53.80%	60.30%	Wales
1.1	Hodgkin lymphoma (all)	640	82.40%	79.60%	84.80%	170	79.60%	73.60%	84.40%	170	87.00%	81.60%	91.00%	Wales
1.2	Mature B-cell neoplasms	7,060	66.60%	65.20%	67.90%	2,050	67.30%	64.70%	69.80%	2,010	71.80%	69.10%	74.30%	Wales
1.3	Mature T-cell and NK-cell neoplasms	480	47.80%	42.30%	53.10%	120	40.70%	30.70%	50.50%	110				Wales
1.4	Unknown lymphoid neoplasms	740	49.60%	45.00%	54.10%	200	54.10%	44.20%	62.90%	180				Wales
2.5	Unknown myeloid neoplasms	80				20				30				Wales

Figure S1: 5-year net survival (%) for adults diagnosed in the period 2009 to 2019 in the UK, and the site-specific variation in survival difference by age groups

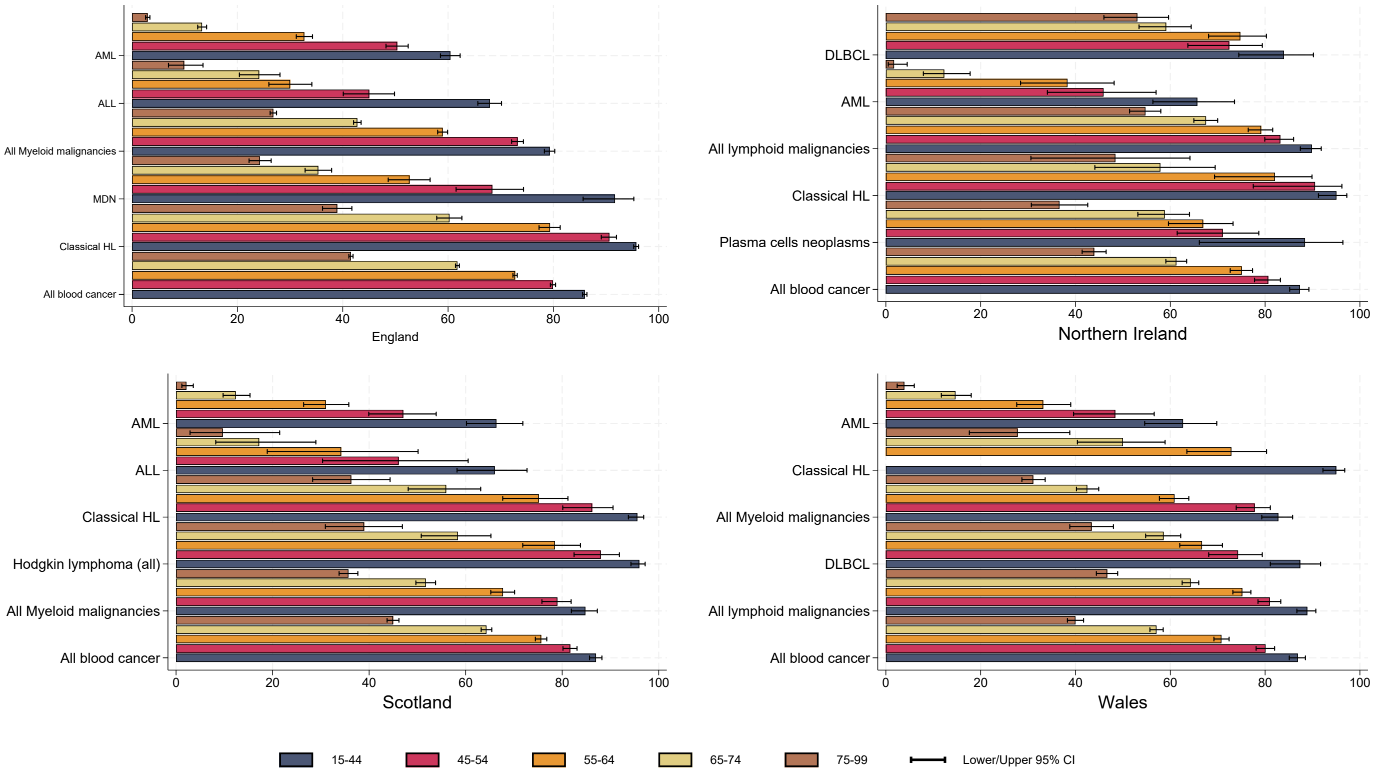

Figure S2: Age-standardised 5-year net survival (%) for adults diagnosed in the period 2009 to 2019 in the UK, and the site-specific variation in survival difference by sex

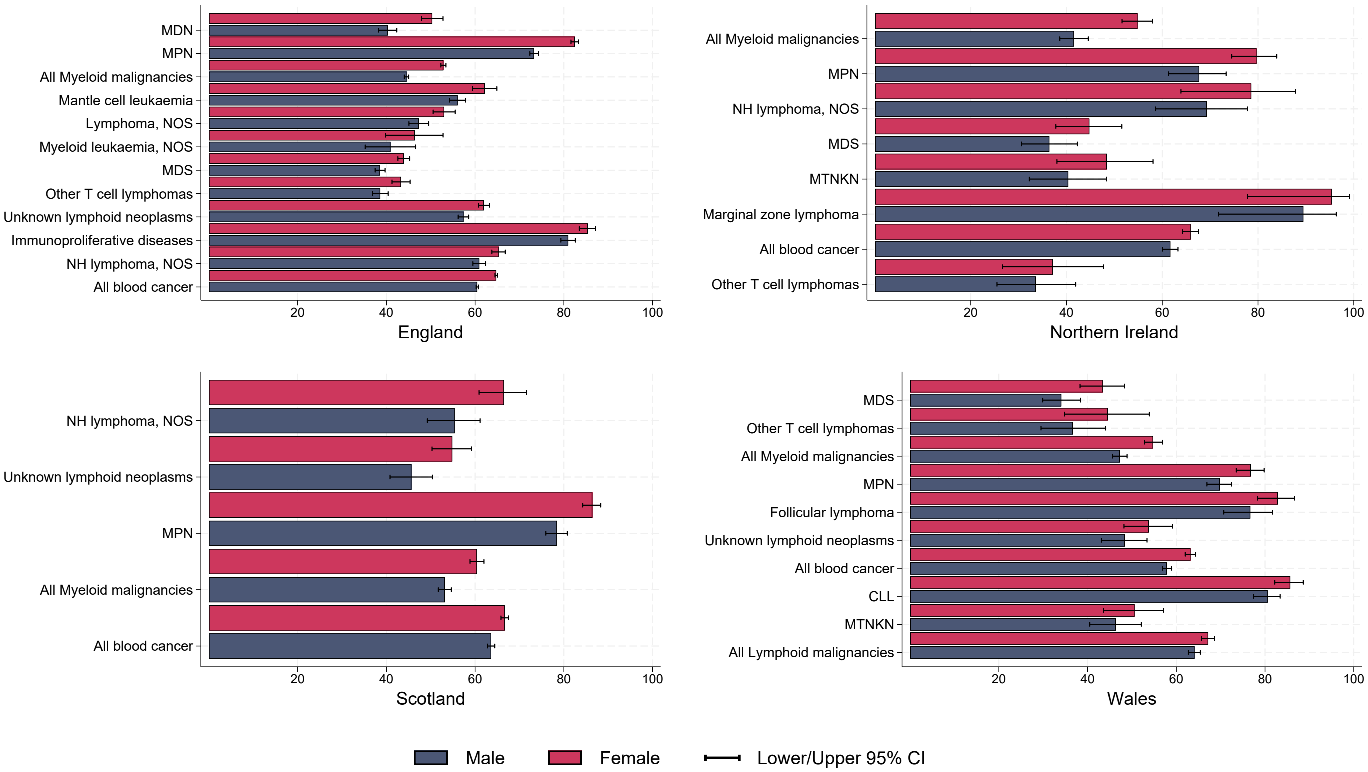

Figure S3: 5-year net survival (%) for adults diagnosed in the period 2009 to 2019 in England and the site-specific variation in survival difference by ethnicity

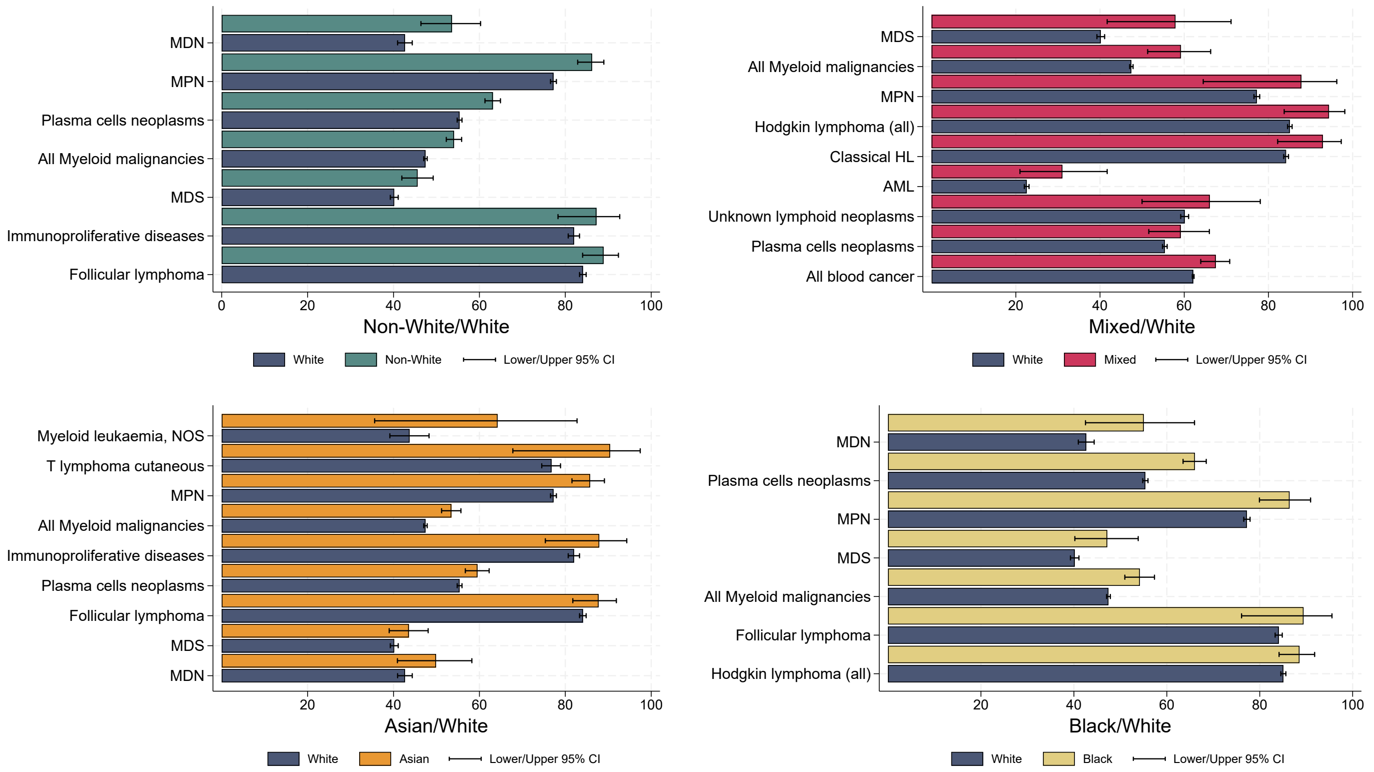

Figure S4: 5-year net survival (%) for adults diagnosed in the period 2009 to 2019 in Wales, and the site-specific variation in survival by rurality

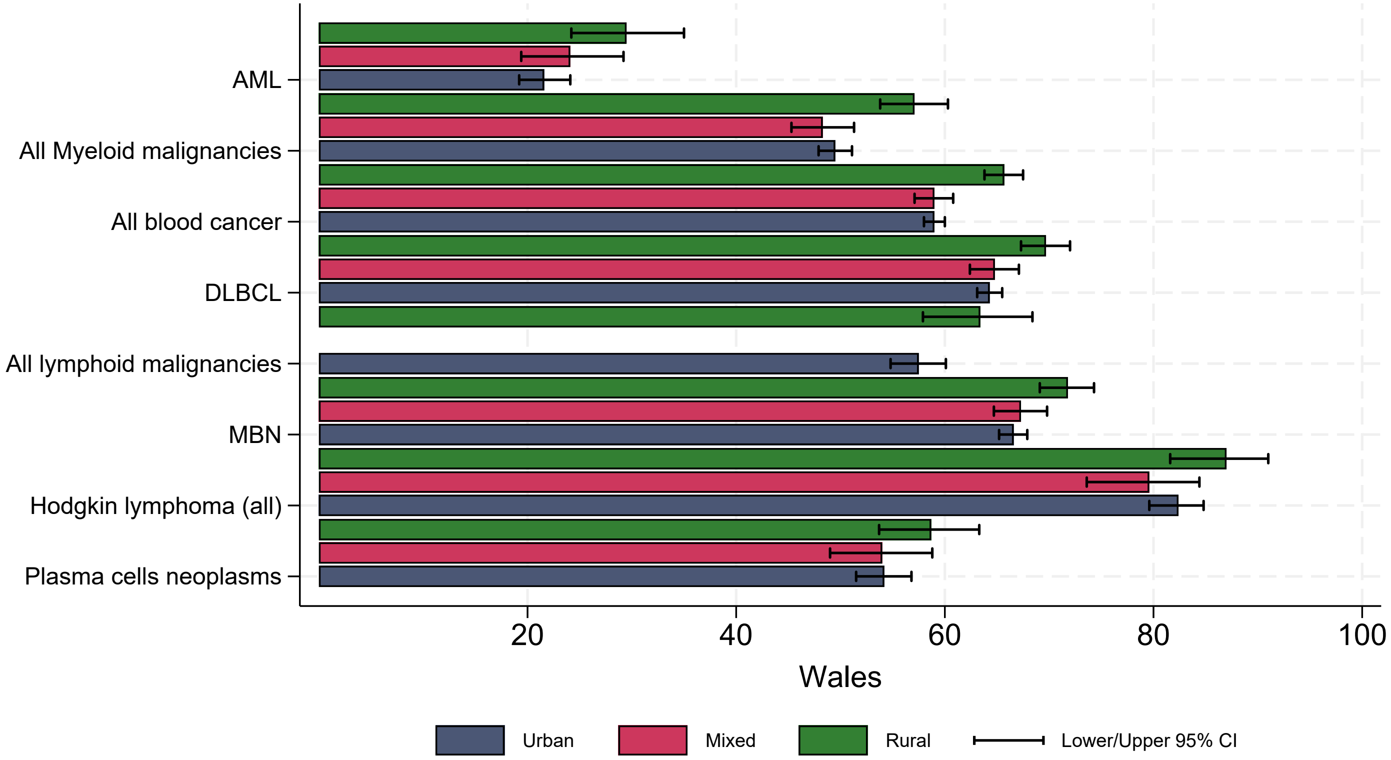

Supplement: Supplementary file 1 — Supplementary information [file 44276_2026_222_MOESM1_ESM.pdf]
